# Integrative analysis for identification of key miRNA-mRNA regulatory axes in esophageal cancer and preliminary validation of the regulatory role of miR-15b-5p/*BTG2* therein

**DOI:** 10.7717/peerj.20538

**Published:** 2026-01-28

**Authors:** Wenyuan Hong, Chaoyang Xia, Gao Li

**Affiliations:** 1 Department of Thoracic Surgery, Hainan General Hospital (Affiliated Hainan Hospital of Hainan Medical University), Haikou, China; 2 Hainan Medical University, Haikou, China

**Keywords:** Esophageal cancer, miRNA-mRNA network, miR-15b-5p/BTG2, Prognostic biomarker, Functional genomics

## Abstract

**Background:**

Esophageal cancer (ESCA), a leading cause of cancer-related mortality, lacks reliable biomarkers for early detection and prognosis. Dysregulated microRNAs (miRNAs) have emerged as pivotal regulators of tumor progression, yet their context-specific roles and interactions with target genes in ESCA remain underexplored.

**Methods:**

Multi-omics data from The Cancer Genome Atlas-esophageal cancer (TCGA-ESCA) and Gene Expression Omnibus (GEO) datasets were integrated to identify differentially expressed miRNAs and mRNAs. A miRNA-mRNA regulatory network was constructed using FunRich and validated through functional assays, including dual-luciferase reporter, quantitative reverse transcription polymerase chain reaction (qRT-PCR) and *in vitro* proliferation/migration/invasion experiments. Prognostic signatures were developed using Cox regression, least absolute shrinkage and selection operator (LASSO)-Cox and nomogram analysis.

**Results:**

We identified 1,131 differentially expressed mRNAs and 69 miRNAs in ESCA. The miR-15b-5p/*BTG2* axis emerged as a central regulatory hub. miR-15b-5p was significantly upregulated in ESCA tissues and showed an inverse correlation with B-cell translocation gene 2 (*BTG2*) expression. Survival analyses established both molecules as independent prognostic factors. Mechanistically, miR-15b-5p directly targeted *BTG2* 3′UTR, suppressing its expression. Functional studies demonstrated that miR-15b-5p overexpression promoted proliferation, migration and invasion in ESCA cells, whereas *BTG2* restoration reversed these effects. A prognostic nomogram integrating miR-15b-5p, *BTG2* and clinical parameters demonstrated robust predictive accuracy (C-index: 0.78).

**Conclusions:**

The miR-15b-5p/*BTG2* axis represents a novel regulatory mechanism in ESCA progression with significant potential as both a prognostic biomarker and therapeutic target.

## Introduction

Esophageal cancer (ESCA), ranking as the sixth leading cause of cancer-related deaths globally, poses significant clinical challenge due to its aggressive behavior and dismal 5-year survival rate (<20%) (Peng et al., 2024; Christodoulidis et al., 2024). The two predominant histological subtypes—esophageal squamous cell carcinoma (ESCC) and esophageal adenocarcinoma (EAC)—exhibit distinct molecular profiles yet share characteristics of late-stage diagnosis, high recurrence and therapeutic resistance (Chen et al., 2023; Wang et al., 2023). Current screening strategies primarily rely on invasive gastrointestinal endoscopy, which faces limitations including suboptimal sensitivity for early-stage lesions and patient discomfort (Yang et al., 2023). Consequently, identifying non-invasive biomarkers for early detection and prognosis prediction is imperative to improve clinical outcomes.

MicroRNAs (miRNAs), small non-coding RNAs approximately 22 nucleotides in length, have emerged as master regulators of tumorigenesis through post- transcriptional modulating of target gene networks (Yu et al., 2024; Smolarz et al., 2022). Notably, circulating miRNAs in serum, plasma and other biofluids offer a minimally invasive window into disease progression, with growing evidence supporting their utility as diagnostic and prognostic biomarkers across cancers (Mestry, Ashavaid & Shah, 2023; Raczkowska et al., 2023; Felekkis & Papaneophytou, 2024). In ESCA, dysregulated miRNAs such as miR-21 and miR-25 have been implicated in metastasis and chemoresistance (Zheng et al., 2023; Guo & Tian, 2023), while miR-29b suppresses tumor growth through Wnt/*β*-catenin signaling (Zang et al., 2015). Despite these advances, systematic exploration of miRNA-mRNA regulatory networks in ESCA, particularly using clinical samples and multi-omics integration, remains limited.

Emerging evidence underscores the tissue-specific duality of miRNAs functions. For instance, miR-15b-5p (a key focus of this study) acts as an oncogene in breast cancer by promoting proliferation, yet functions as a tumor suppressor in thyroid malignancies through apoptosis induction (Wu et al., 2020; Zou et al., 2020). This context-dependent behavior highlights the critical need for disease-specific mechanistic investigations. Here, we bridge this gap by integrating transcriptomic data from The Cancer Genome Atlas (TCGA) with functional validation to construct a comprehensive miRNA-mRNA regulatory network in ESCA. Our study identifies the miR-15b-5p/*BTG2* axis as a novel driver of ESCA progression and elucidates its prognostic significance through advanced bioinformatics modeling.

By leveraging multi-omics approaches, we establish a translational framework for converting miRNA-mRNA interactions into clinically actionable biomarkers. This strategy addresses the urgent need for precision medicine in ESCA management, potentially enabling non-invasive diagnostic and targeted therapies. The complete analysis workflow is depicted in Fig. S1.

## Materials and Methods

### Data acquisition

All datasets were sourced from public repositories with explicit accession codes: miRNA/mRNA expression and clinical data from The Cancer Genome Atlas Esophageal Carcinoma cohort (Project ID: TCGA-ESCA; https://portal.gdc.cancer.gov/). Meanwhile, we also downloaded the corresponding clinicopathological data (Materials S1). miRNA expression dataset from the Gene Expression Omnibus (GEO) (accession: GSE13937; PMID: 19789312). mRNA expression dataset from GEO (accession: GSE45670; PMID: 24769072).

### Screening of differentially expressed miRNAs and mRNAs in TCGA-ESCA

Differentially expressed miRNAs and mRNAs between ESCA tumors and adjacent normal tissues were identified using Sangerbox 3.0 (http://sangerbox.com/home.html). First, missing values in the expression profiles were imputed using the k-Nearest Neighbor (KNN) method (*k* = 10) (Siddalingappa & Kanagaraj, 2023). Subsequently, differential expression analysis was performed using the edgeR package (v3.40.2) with the following thresholds: |log2 fold change (FC)| ≥ 1, Benjamini–Hochberg adjusted *p*-value <0.05 and false discovery rate (FDR) < 0.05 (Pei et al., 2023). For the TCGA-ESCA mRNAs, protein-coding gene expression profiles were extracted using Sangerbox 3.0 based on GENCODE annotation files (gencode.v22.annotation.gtf) prior to differential expression screening.

### Construction of miRNA-mRNA regulatory network

The miRNA-mRNA regulatory networks were constructed using FunRich (v3.1.3), an independent software tool for functional enrichment and interaction network analysis of genes and proteins (http://www.funrich.org) (Sultana et al., 2023). Initially, target genes of differentially expressed miRNAs were predicted using FunRich with high-stringency thresholds: context score >0.4 and conservation across ≥3 species. Only targets with experimental validation (miRTarBase) or computational prediction (TargetScan) were retained. Subsequently, miRNA-mRNA expression correlations were analyzed in the TCGA-ESCA dataset, and significant regulatory pairs (Pearson —r—>0.6, *p* < 0.05) were selected for network construction. Finally, the regulatory networks were visualized using Cytoscape (v3.8.0).

### Gene Ontology (GO) function and Kyoto Encyclopedia of Genes Genomes (KEGG) pathway enrichment analysis

Functional enrichment analysis of mRNAs within the miRNA-mRNA regulatory network was performed using the online Sangerbox 3.0 platform. The analysis utilized the Molecular Signatures Database (MSigDB) subset (c5.go.mf.v7.4.symbols.gmt; available at http://www.gsea-msigdb.org/gsea/downloads.jsp) as the background gene set, Genes were mapped to the background set, and enrichment analysis was conducted using the cluster Profiler R package (v 3.14.3) to identify significantly enriched gene sets. The analysis employed the following parameters: gene set size limits of 5-5000 genes, significance thresholds of *p* < 0.05 and FDR < 0.25, and the Molecular Signatures Database (MSigDB c5.go.mf.v7.4) as background (Pei et al., 2024).

### Survival signatures and survival analysis

Firstly, the R (v4.2.1; R Core Team, 2022) software package “survival (v3.3.1)” was used to integrate the survival time, survival status of patients in TCGA-ESCA and all the data of miRNA expression in miRNA-mRNA regulatory network for Kaplan–Meier survival analysis with log-rank *p* < 0.05 significance threshold. Subsequently, Univariate Cox regression using *p* < 0.05 as inclusion criterion for prognostic miRNA selection was performed using the coxph function of R package “survival (v3.3.1)”. The selected miRNAs were analyzed by stepwise Multivariate Cox regression analysis using R packet “survminer (v0.4.9)” to screen out the key miRNAs with *p* < 0.05 significance threshold. Finally, the miRNAs screened by Multiple Cox regression was used to construct the signatures related to the survival and prognosis of ESCA.

According to the median risk value, patients with ESCA were divided into high-risk group and low-risk group. Time-dependent receiver operator characteristic (ROC) analysis used survivalROC v1.0.3.1 with Kaplan–Meier estimation. Survival analyses employed survival v3.3.1 and survminer v 0.5.0 with log-rank significance threshold of *p* < 0.05. “ggplot2(v3.5.2)” was used to draw the relationship between risk score and gene expression.

### Least absolute shrinkage and selection operator-Cox regression analysis and multivariate survival regression alignment diagram

The R software package “glmnet (v4.1.6)” was used to integrate the survival time, survival status and gene expression data of patients in TCGA-ESCA, and least absolute shrinkage and selection operator (LASSO)-Cox regression was implemented *via* glmnet v4.1.6 with L1 penalty (*α* = 1). The optimal *λ* value (*λ* = 0.05, 1 SE criterion) was selected through 10-fold cross-validation minimizing partial likelihood deviance. LASSO-Cox regression was applied to address high-dimensionality (271 mRNAs) and multicollinearity, utilizing L1-penalization to shrink coefficients of non-informative variables to zero, thereby selecting robust predictors while minimizing overfitting. The optimal penalty parameter (*λ*) was determined through 10-fold cross-validation, ensuring model generalizability by minimizing partial likelihood deviance.

The R software package “rms (v8.0.0)” was used to integrate the survival time, survival status and characteristic data of TCGA-ESCA patients. and the nomogram was established by Cox method to evaluate the prognostic significance of these characteristics in all samples.

### Cell culture and cell transfection

Human esophageal cancer cell lines EC109 were procured from BeNa Culture Collection (BNCC342591; BNCC), which were authenticated by the vendor using short tandem repeat (STR) profiling prior to distribution (Report ID: BNCC342591). Upon receipt, cells were expanded and cryopreserved within 10 passages. For all experiments, cells were thawed and cultured for ≤6 weeks. During this period, cells were routinely monitored and remained free of mycoplasma contamination. No further ethics approval was required as per institutional guidelines for commercially available cell lines. EC109 was cultivated in Roswell Park Memorial Institute-1640 (RPMI-1640; Gibco, Waltham, MA, USA) with 10% fetal bovine serum (FBS; Gibco) in 5% CO_2_ at 37 °C.

Cell transfection (miR-15b-5p mimics or inhibitor as well as the *BTG2* overexpression vector or *BTG2* siRNA into EC109 cell) was performed with Lipofectamine 3000 (Invitrogen, Waltham, MA, USA) according to the manufacturer’s instructions. miR-15b-5p mimics, miR-15b-5p mimic NC, miR-15b-5p inhibitor, miR-15b-5p inhibitor NC, *BTG2* overexpression vector and *BTG2* siRNA were purchased from Ribobio (China). NC groups received scrambled oligonucleotides at identical concentrations (50 nM) and transfection conditions (Lipofectamine 3000) as experimental groups.

### Quantitative real time-polymerase chain reaction

Total RNA of cell was extracted from cells using RNA Easy Fast Animal Tissue/Cell Total RNA Extraction Kit (DP451; Tiangen). RNA concentration and purity were quantified using a Nanodrop 2000 (Thermo Fisher Scientific, Waltham, MA, USA). Reverse transcription and quantitative real-time polymerase chain reaction (qRT-PCR) were performed using the FastKing One-Step Reverse Transcription Fluorescence Quantification Kit (SYBR Green) (FP313, Tiangen, China) according to the manufacturer’s protocol. The miR-15b-5p primers (MQPS0000690-1-100) and U6 primers (MQPS0000002-1-200) were purchased from Ribobio (China). *BTG2* primers were: forward primer (5′–3′)- ACGGGAAGGGAACCGACAT, Reverse primer (5′–3′)-CAGTGGTGTTTGTAGTGCTCTG. *β*-*actin*: forward primer (5′–3′)- CACTCTTCCAGCCTTCCTTC, Reverse primer (5′–3′)- GTACAGGTCTTTGCGGATGT). All mRNA primers were synthesized by Sangon (China). *β*-actin served as the endogenous control for mRNA normalization, while U6 snRNA was used for miRNA normalization. Reactions were performed in triplicate for each sample group. Relative gene expression was calculated using the 2^−ΔΔCt^ method.

### Cell proliferation assay

EC109 cells in logarithmic growth phase were harvested, quantified and seeded into 96-well plates at 5,000 cells per well. After 24 h incubated, cells were transfected with plasmid or miRNA mimic and cultured for 48 h, Subsequently, 10 µl CCK-8 solution (C0038, Beyotime, China) was added to each well followed by 3 h incubation. Absorbance at OD450 (OD_4__5__0_) was measured using an automatic microplate reader. All experiments included three technical replicates per condition.

### Wound-healing assay

EC109 cells in logarithmic growth phase were harvested, quantified and seeded into 24-well plates with 5 ×10^5^ cells per well. Following 24 h incubation and transfection with plasmid or miRNA mimic, t cells were cultured for 6 h. The monolayer was then scratched using a sterile 200 µl pipette tip to create uniform wounds. After washing with PBS to remove detached cells, serum-free medium was added. Wound closure was monitored over 48 h with migration areas photographed at 0 h and 48 h using phase-contrast microscopy. Migration rates were quantified using ImageJ software.

### Cell invasion assay

Cell invasion was assessed using Corning Transwell chambers (8-µm pore size). After transfection of plasmid and miRNA mimic, the EC109 cells were cultured for 6 h. EC109 cells were harvested and resuspended in serum-free medium. Cell suspensions (1 × 10^4^ cells/well) were plated in Matrigel (BD, Franklin Lakes, NJ, USA) upper chamber (Corning, Corning, NY, USA). The lower chambers contained 500 µl RPMI-1640 with 20% FBS as chemoattractant. Following 48 h incubation, non-invading cells were removed from the upper chamber. Invaded cells on the membrane underside were fixed with 4% paraformaldehyde (Sigma-Aldrich, St. Louis, MO, USA), stained with 0.1% crystal violet (Sigma-Aldrich, St. Louis, MO, USA) and counted in five random fields per well using an Olympus microscope. Experiments included three biological replicates.

### Dual-luciferase assay

Reporter plasmids pmiR-RB-Report™-*BTG2*-wt and pmiR-RB-Report™-*BTG2*-mut were purchased from Ribobio (China). EC109 cells were co-transfected with reporter plasmids and miRNA mimics using Lipofectamine 3000. After 48 h, luciferase activity was measured using the Dual-luciferase reporting kit (Promega, Madison, WI, USA) according to manufacturer’s protocol. Experiments included three biological replicates with triplicate technical replicates per condition.

### Statistical analysis

All data are presented as mean ± standard deviation (SD) from three biological replicates. Intergroup differences were analyzed using two-tailed student’s *t*-test. Statistical significance was determined at *p* < 0.05. All analyses were performed using GraphPad Prism 8.

## Results

### Identification of differential expressed miRNAs and mRNAs in esophageal carcinoma (ESCA)

**Figure 1:**
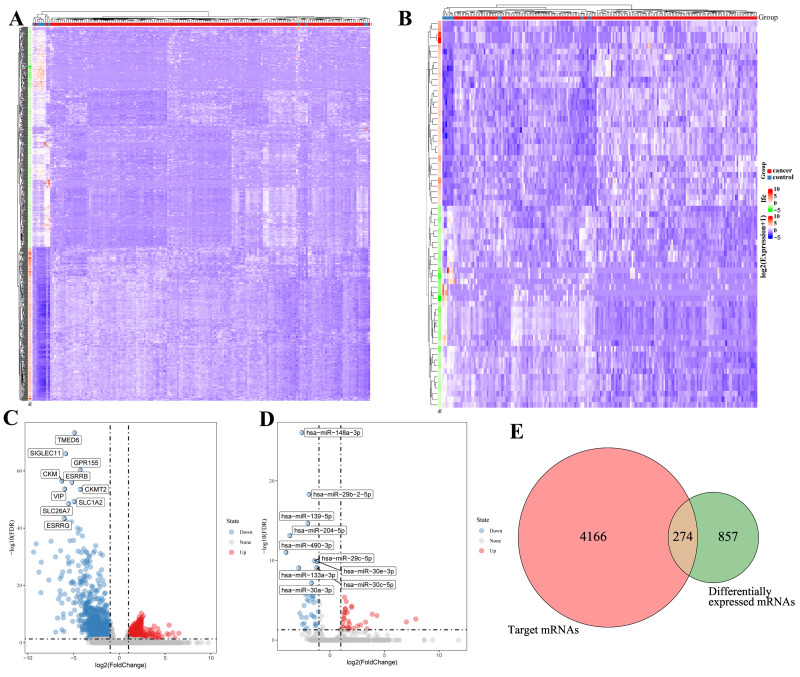
Integrated profiling of differentially expressed mRNAs and miRNAs in TCGA-ESCA cohort. (A) Heatmap for the differentially expressed mRNAs in the TCGA-ESCA. (B) Heatmap for the differentially expressed miRNAs in the TCGA-ESCA. (C) Volcano plot for the differentially expressed mRNAs in the TCGA-ESCA. Red dots represent up regulation, blue dots represent down regulation, and gray dots represent non differentially expressed mRNAs. (D) Volcano plot for the differentially expressed miRNAs in the TCGA-ESCA. Red dots represent up regulation, blue dots represent down regulation, and gray dots represent non differentially expressed miRNAs. (E) Venn diagram shows the overlapping mRNAs between the target mRNAs of miRNAs and differentially expressed mRNAs.

Systematic analysis of TCGA-ESCA data identified 1,131 differentially expressed mRNAs (348 upregulated and 783 downregulated) and 69 dysregulated miRNAs (33 upregulated and 36 downregulated). Among the top altered molecules, miR-15b-5p (upregulated) and *BTG2* (downregulated) exhibited the most pronounced changes (Table 1). Unsupervised clustering confirmed distinct expression patterns between tumor and normal tissues (Figs. 1A– 1D), establishing a transcriptional landscape unique to ESCA.

**Table 1:** Top 10 DEmiRNAs and DEmRNAs with the lowest *p* value in normal tissues and ESCA tissues.

**RNA**	**logFC**	**logCPM**	***P* value**	**FDR**
**miRNAs**				
hsa-miR-148a-3p	−2.58112908	14.94973001	4.01E−30	8.36E−27
hsa-miR-29b-2-5p	−1.931342903	4.526950297	2.30E−22	4.79E−19
hsa-miR-139-5p	−2.032347645	5.721099461	9.93E−19	2.07E−15
hsa-miR-204-5p	−3.686493848	2.859398255	3.65E−17	7.59E−14
hsa-miR-490-3p	−4.035923121	2.18755313	4.55E−15	9.44E−12
hsa-miR-29c-5p	−1.440348273	3.986952717	5.33E−14	1.11E−10
hsa-miR-30e−3p	−1.166856295	11.65884406	6.30E−14	1.31E−10
hsa-miR-30c-5p	−1.252360189	9.438317197	3.77E−13	7.83E−10
hsa-miR-133a-3p	−2.867995943	5.841720026	4.08E−13	8.46E−10
hsa-miR-30a-3p	−1.713562425	11.14459633	3.23E−11	6.69E−08
**mRNAs**				
TMED6	−4.927101363	1.428339342	2.42E−78	4.77E−74
SIGLEC11	−5.879408387	0.850452201	4.94E−71	9.74E−67
GPR155	−4.287065463	5.512104629	2.33E−65	4.60E−61
CKM	−6.298631101	1.738414017	1.71E−61	3.36E−57
ESRRB	−5.21003031	0.461441712	5.14E−61	1.01E−56
VIP	−5.982353796	0.854028703	1.03E−58	2.03E−54
CKMT2	−4.288308886	1.284184513	1.32E−58	2.59E−54
SLC1A2	−4.964086633	2.045235746	2.80E−54	5.53E−50
SLC26A7	−5.579974162	1.34642446	1.53E−53	3.02E−49
ESRRG	−6.039890292	3.422635895	1.93E−48	3.80E−44

### Construction of miRNA-mRNA regulatory network

**Figure 2:**
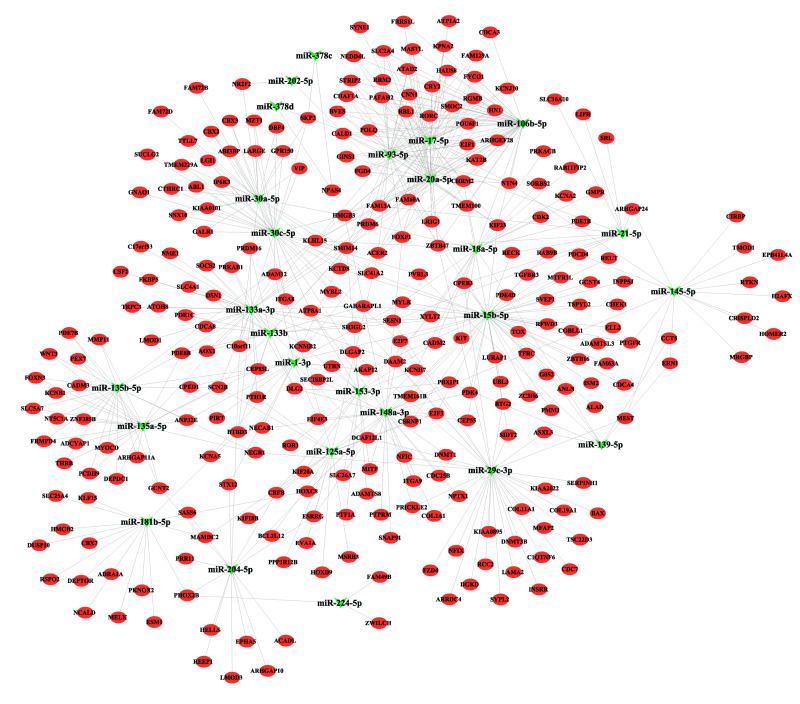
MiRNA-mRNA regulatory network. miRNA-mRNA regulatory network. The green invaginated triangle represents miRNAs and the red oval represents mRNAs.

To elucidate potential functional interactions, we predicted targets of the 69 dysregulated miRNAs using FunRich, identifying 4,440 potential targets. (Material S2). Intersection with differentially expressed mRNAs identified 274 overlapping mRNAs (Fig. 1D), suggesting miRNA-mediated regulation. The resultant regulatory network comprised 26 miRNAs and 271 mRNAs connected by 576 edges (Fig. 2). Topological analysis highlighted miR-15b-5p as a central hub node directly targeting 18 mRNAs, including *BTG2*, positioning it as a key regulator in ESCA pathogenesis.

### Functional enrichment implicates proliferation and metabolic pathways

**Figure 3:**
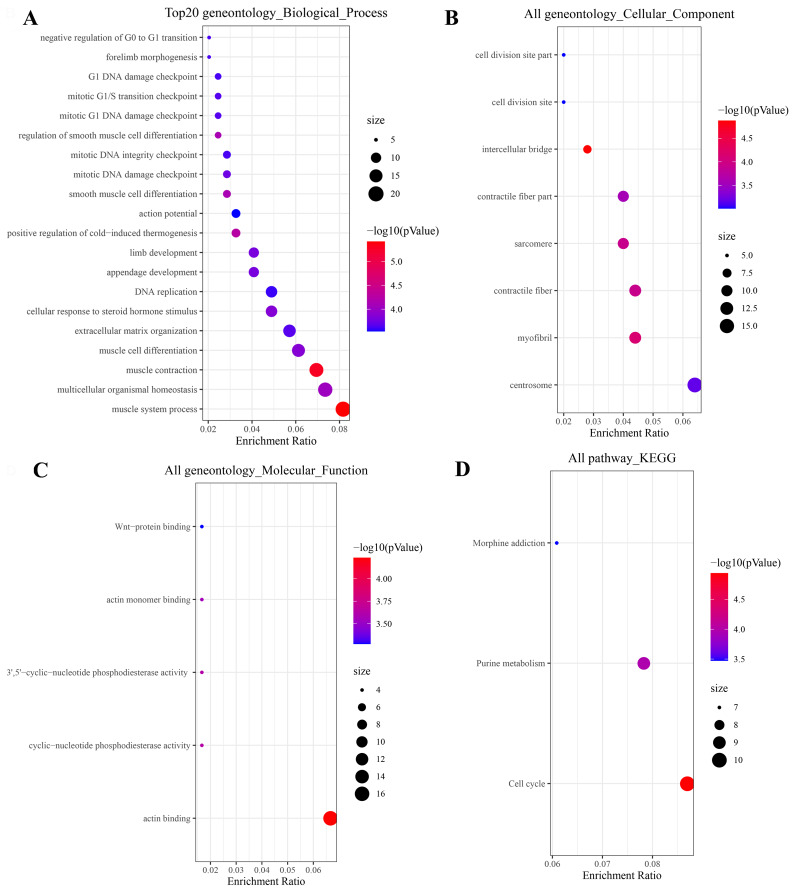
Functional enrichment analysis of miRNA-mRNA network genes. (A–C) Enriched GO clusters for the mRNAs in miRNA-mRNA regulatory network. BP, biological processes; CC, cell composition; MF, molecular functions. (D) Enriched KEGG clusters for the mRNAs in miRNA-mRNA regulatory network. KEGG, Kyoto Encyclopedia of Genes and Genomes.

Gene Ontology (GO) and Kyoto Encyclopedia of Genes and Genomes (KEGG) analyses of network genes revealed significant enrichment in biologically coherent processes. Biological process (BP) terms were significantly enriched in myocyte differentiation, muscle contraction, DNA replication, and mitotic regulation (Fig. 3A). Molecular function (MF) analysis highlighted associations with actin binding, WNT protein binding and phosphodiesterase activity (Fig. 3B). Cellular component (CC) terms revealed enrichment in myofibrils, intercellular bridges, and centrosomes (Fig. 3C). KEGG pathway analysis further identified key oncogenic pathways, including cell cycle, purine metabolism, and morphine addiction (Fig. 3D). These findings collectively suggest the miRNA-mRNA network orchestrates cell proliferation, cytoskeletal dynamics and metabolic reprogramming during ESCA progression.

### Prognostic miRNA signature construction and survival analysis

**Figure 4:**
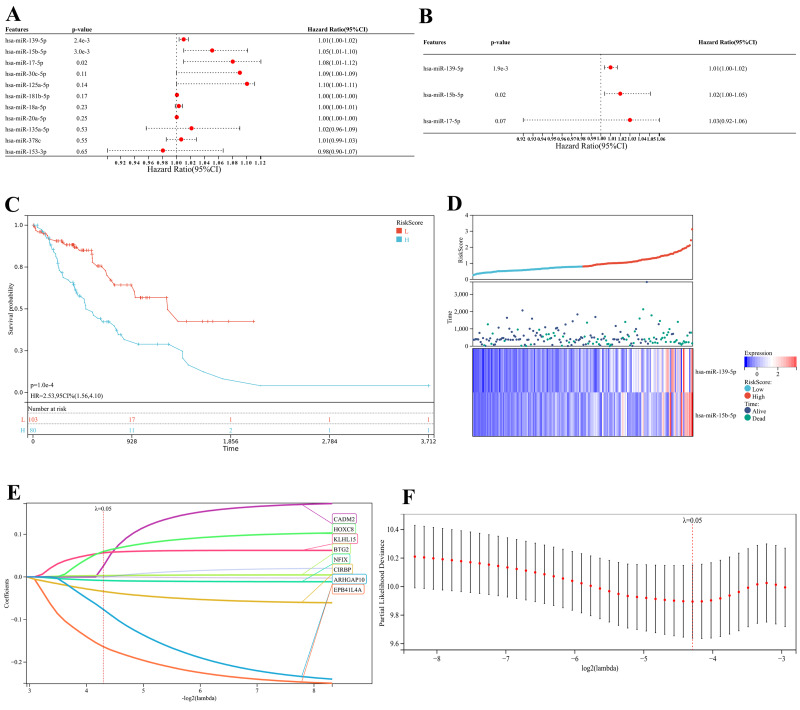
Construction and validation of prognostic risk models for ESCA. (A) Forest map: univariate COX regression analysis result, high risk miRNAs: HR>1; Low risk miRNAs: HR<1. (B) Forest map: Multivariate COX regression analysis result, high risk miRNAs: HR>1; Low risk miRNAs: HR<1; (C) Kaplan–Meier curves of overall survival in the high and low risk groups of ESCA patients in the TCGA-ESCA. L, low risk; H, high risk. (D) Heatmap of 2-miRNAs expression patterns and distribution of risk scores in the TCGA-ESCA. (E) LASSO logistic regression analysis to screen prognostic mRNAs. Each color represents a mRNA. (F) Plot of partial likelihood deviance of LASSO logistic regression analysis.

Kaplan–Meier analysis of the 26 network miRNAs identified 11 significantly associated with poor overall survival (log-rank *p* < 0.05), which were significantly associated with patient prognosis, underscoring their potential clinical relevance in ESCA (Fig. S2). Univariate Cox regression of these 11 miRNAs revealed three candidates—miR-139-5p, miR-15b-5p and miR-17-5p—as significant predictors of OS (Fig. 4A). Subsequent Multivariate Cox regression refined a robust 2-miRNA signature (miR-139-5p and miR-15b-5p) that stratified patients into high/low-risk groups (HR = 2.84, 95%CI [1.72–4.68]; Fig. 4B). Patients in the high-risk group exhibited significantly reduced OS (log-rank *p* < 0.001) (Fig. 4C), with risk scores strongly correlating with miRNA expression and mortality (Fig. 4D). The risk score demonstrated strong prognostic independence in both univariate and multivariate analyses. Given prior studies on miR-139-5p in ESCA, miR-15b-5p was selected for mechanistic exploration.

### Independent validation prioritizes the miR-15b-5p/*BTG2* axis

**Figure 5:**
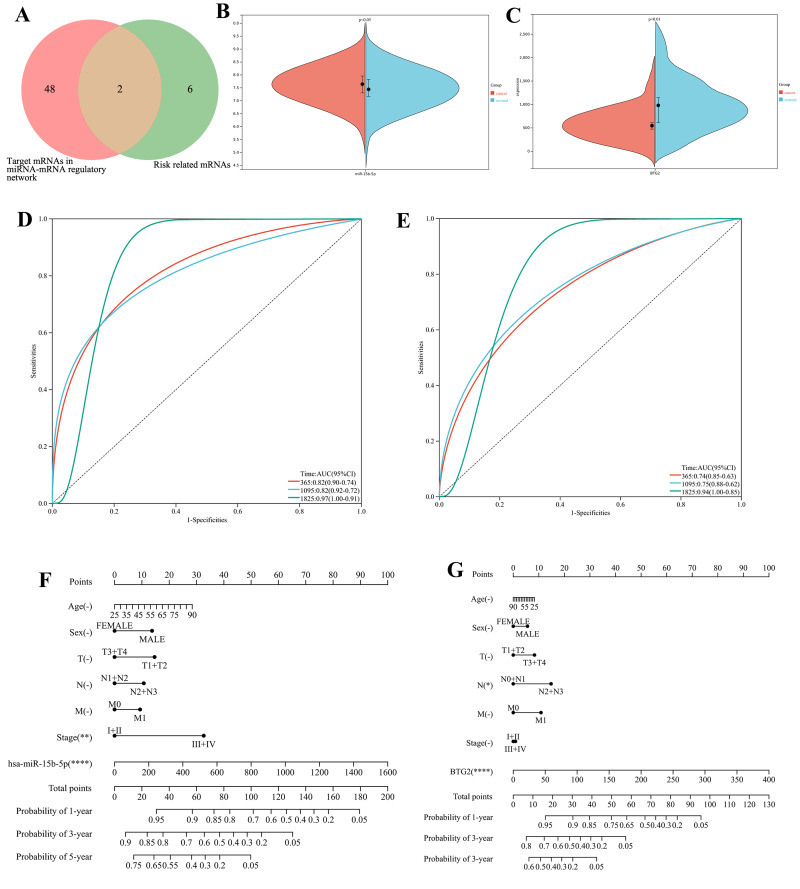
Clinical significance of miR-15b-5p and BTG2 in ESCA prognosis. (A) Venn diagram shows the overlapping mRNAs between the mRNAs in miRNA-mRNA regulatory network and prognostic mRNAs by LASSO logistic regression analysis. (B) The expression levels of miR-15b-5p in ESCA and adjacent noncancer tissues of GSE13937. (C) The expression levels of BTG2 in ESCA and adjacent noncancer tissues of GSE13937. (D) ROC curves of miR-15b-5p for overall survival of ESCA patients at 1- year, 3- years, and 5-years. (E) ROC curves of BTG2 for overall survival of ESCA patients at1- year, 3- years, and 5-years. (F) Nomogram to predict the probability of overall survival at 1- year, 3- year, and 5-year for the prognosis of ESCA patients with clinical parameters and miR-15b-5p. ^∗∗∗∗^*p* < 0.0001. ^∗∗^ *p* < 0.01. (G) Nomogram to predict the probability of overall survival at 1- year, 3- year, and 5-year for the prognosis of ESCA patients with clinical parameters and BTG2. ^∗∗∗∗^*p* < 0.0001.

LASSO-Cox regression analysis of 271 network mRNAs identified eight prognostic genes (*CADM2, KLHL15, EPB41L4A, CIRBP, BTG2, HOXC8, NFIX* and *ARHGAP10*) with 10-fold cross-validation selecting the optimal penalty parameter (*λ* = 0.05) (Fig. S3). Intersection with miR-15b-5p targets confirmed *BTG2* as a key downstream effector (Fig. 5A). External validation using GEO datasets (GSE13937 for miR-15b-5p; GSE45670 for *BTG2*) confirmed miR-15b-5p upregulation and *BTG2* downregulation (Figs. 5B and 5C) in ESCA. Subtype-specific re-analysis further validated miR-15b-5p dysregulation in both major histological subtypes: ESCC (GSE13937, *n* = 88, *p* < 0.05) and EAC (GSE13937, *n* = 64, *p* < 0.01) *versus* matched normal tissues (Figs. S4A and 4B). This consolidated its pan-subtype relevance in ESCA pathogenesis. Time-dependent ROC analysis demonstrated robust prognostic performance for miR-15b-5p (AUC = 0.82, 0.82 and 0.97 for 1-, 3- and 5-year survival) and *BTG2* (area under curve (AUC) = 0.74, 0.75 and 0.94) (Figs. 5D and 5E). A clinically applicable nomogram integrating miR-15b-5p, *BTG2* and key clinical variables (TNM stage, age and gender) showed high predictive accuracy (C-index of miR-15b-5p = 0.78, 95%CI (0.67−0.79), *p* = 3.70 ×10^−^^15^; C-index of *BTG2* = 0.72, 95%CI [0.77–0.89], *p* = 6.07 ×10^−^^28^; Figs 5F, 5G, Figs. S4C and S4D).

### Functional validation of the miR-15b-5p/*BTG2* axis

**Figure 6:**
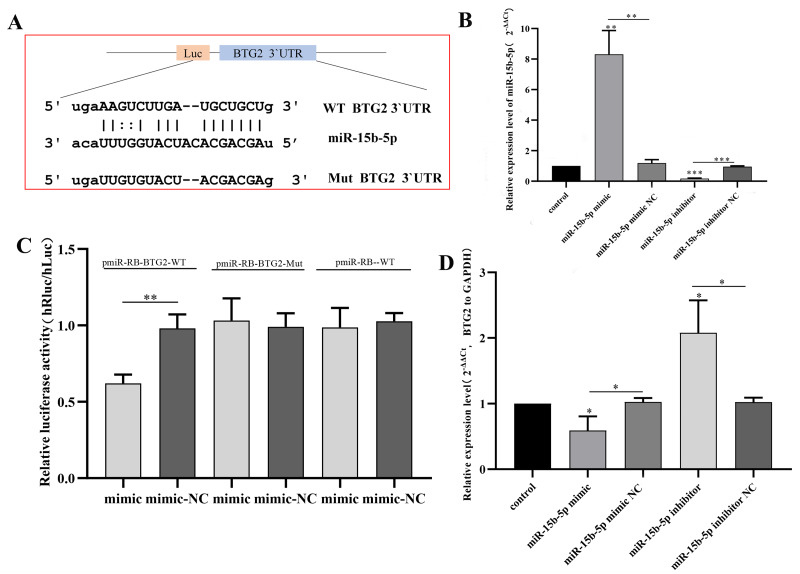
Functional validation of miR-15b-5p targeting *BTG2*. For all panels, NC (negative control) groups received scrambled oligonucleotides under identical transfection conditions (50 nM, Lipofectamine 3000) as experimental groups. (A) Schematic of the predicted binding sites between miR-15b-5p and the wild-type (wt) or mutant (mut) *BTG2* 3′ UTR. (B) Transfection efficiency of miR-15b-5p mimics and inhibitor in EC109 cells, verified by RT-qPCR. Data were compared to the mimic NC or inhibitor NC group, respectively. Data are presented as mean ± SD. ^∗∗∗^*p* < 0.001, ^∗∗^*p* < 0.01. (C) Dual-luciferase reporter assay confirming the direct binding of miR-15b-5p to the *BTG2* 3′ UTR. Luciferase activity was compared between the indicated groups. Data are presented as mean ± SD. ^∗∗^*p* < 0.01. (D) *BTG2* mRNA expression levels following modulation of miR-15b-5p. Data were compared to the mimic NC or inhibitor NC group, respectively. Data are presented as mean ± SD. ^∗^*p* < 0.05, ^∗∗∗^*p* < 0.001.

**Figure 7:**
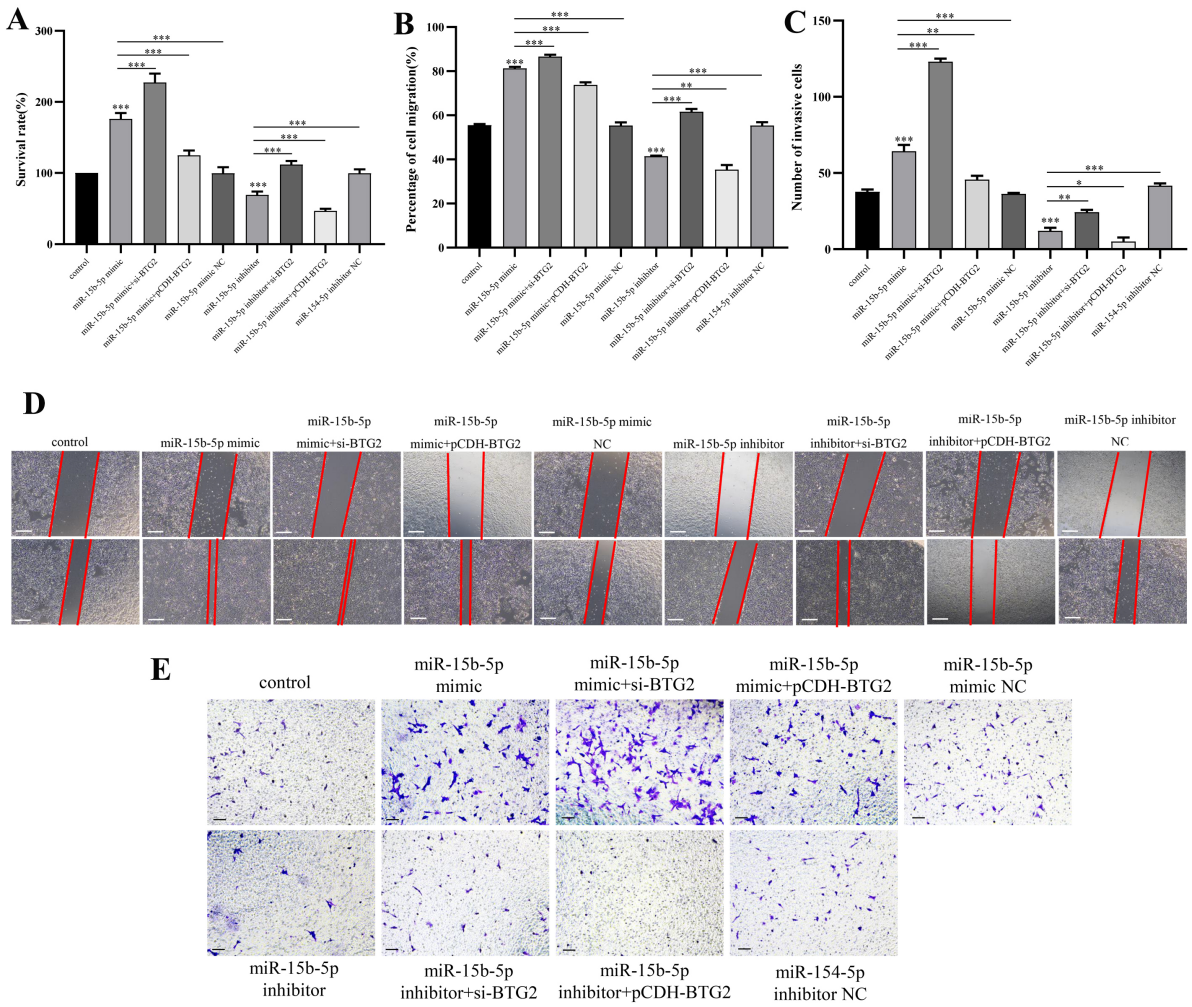
Functional impacts of the miR-15b-5p/*BTG2* axis on EC109 cell malignancy. For all panels, NC (negative control) groups received scrambled oligonucleotides or empty vectors under identical transfection conditions as experimental groups. (A) Cell viability measured by CCK-8 assay under different perturbations of miR-15b-5p and *BTG2*. Data were compared to the mimic NC + Vector group. Data are presented as mean ± SD (*n* = 3 biological replicates). ^∗∗∗^*p* < 0.001. (B) Representative images from the wound-healing assay showing cell migration capabilities (scale bar: 100 µm, ×40 magnification). (C) Representative images from the Transwell invasion assay (scale bar: 100 µm, ×40 magnification). (D) Quantification of the migrated area from the wound-healing assay. Data were compared as indicated. Data are presented as mean ± SD. ^∗∗∗^*p* < 0.001, ^∗∗^*p* < 0.01. (E) Quantification of invaded cells per field from the Transwell assay. Data were compared as indicated. Data are presented as mean ± SD (*n* = 3 biological replicates). ^∗∗∗^*p* < 0.001, ^∗∗^*p* < 0.01, ^∗^*p* < 0.05.

Dual-luciferase assays confirmed direct binding of miR-15b-5p to the *BTG2* 3′ UTR (*p* < 0.01; Figs. 6A–6C). Modulating miR-15b-5p expression inversely regulated *BTG2* mRNA levels (*p* < 0.05; Fig. 6D). Functionally, miR-15b-5p overexpression enhanced EC109 cell proliferation, migration and invasion (*p* <  0.001), while its inhibition suppressed these phenotypes. Critically, *BTG2* knockdown amplified oncogenic effects, whereas *BTG2* overexpression reversed miR-15b-5p-driven malignancy (Figs. 7A–7E), confirming a causal regulatory axis.

## Discussion

MicroRNAs (miRNAs) represent a critical layer of post-transcriptional regulation in cancer biology, yet their context-specific roles in ESCA remain underexplored (Yu et al., 2024; Smolarz et al., 2022). Our integrated analysis identifies miR-15b-5p/*BTG2* as a novel regulatory axis driving ESCA progression. Unlike its tumor-suppressive role in thyroid cancer (Zou et al., 2020), miR-15b-5p exhibits context-dependent oncogenicity in ESCA by directly suppressing *BTG2* to promote proliferation and invasion. These resolves contradictions regarding miR-15b-5p functional duality and addresses a critical knowledge gap in ESCA pathogenesis, where research has predominantly focused on miR-25 and miR-29b (Guo & Tian, 2023; Zang et al., 2015).

The tissue-specific behavior of miR-15b-5p (encoded by MIR15B at 3q25.33) highlights its complex role across malignancies (Karam-Palos et al., 2023). A pan-cancer analysis revealed that promoter hypomethylation drives miR-15b-5p overexpression in gastrointestinal cancers (Wu et al., 2020), aligning with our observation in ESCA. This epigenetic dysregulation is well-established in gastrointestinal malignancies. For instance, Wu et al. (2020) demonstrated that hypomethylation of the *MIR15B* promoter in breast and gastric cancers directly correlated with elevated miR-15b-5p levels, promoting metastasis through target gene suppression. Similarly, Karam-Palos et al. (2023) highlighted that oxidative stress-induced epigenetic modifications (*e.g.*, DNA demethylation, histone acetylation) can dysregulate miRNAs across malignancies. In ESCA, hypermethylation of tumor-suppressor genes (*e.g.*, CDKN2A) is common (Chen et al., 2023), while oncogenic miRNAs like miR-25 are activated *via* promoter demethylation (Guo & Tian, 2023). Although further validation is needed, these findings suggest that epigenetic therapies targeting DNA methyltransferases (*e.g.*, DNMT inhibitors) could potentially restore miR-15b-5p/*BTG2* axis homeostasis in ESCA.

The B-cell translocation gene 2 (*BTG2*) is a key gene in the BTG/TOB family, which is involved in various life processes of the body, including cancer (Luo et al., 2025). It has been reported that *BTG2* is under expressed in many human cancers and acts as a tumor suppressor (Song et al., 2023). In ESCA, the role of *BTG2* in ESCA has been little studied. Wang et al. (2021) showed that expression level of *BTG2* was an independent risk factor affecting the prognosis of ESCC patients through a series of biosynthesis methods, and could be used as a molecular marker to identify and predict the progression of ESCC, which was consistent with our nomogram multivariate regression analysis. The consistent dysregulation of the miR-15b-5p/*BTG2* axis across both major ESCA subtypes is biologically and clinically significant. ESCC and EAC originate from distinct lineages and have divergent causes. The convergence of this regulatory axis therefore suggests it represents a common fundamental mechanism driving ESCA progression. This pan-subtype role significantly enhances the potential translational utility of our findings, as it positions the miR-15b-5p/*BTG2* axis as a promising prognostic biomarker applicable to a broader patient population, irrespective of histological classification. From a therapeutic standpoint, this implies that strategies aimed at targeting this pathway—for instance, inhibiting miR-15b-5p or restoring *BTG2* function—could hold promise for a wider spectrum of ESCA patients. Future work should determine if the upstream drivers of miR-15b-5p overexpression are shared between subtypes or are distinct. Additionally, single-cell RNA-seq studies in ESCC have recently uncovered *BTG2* role in suppressing cancer stemness *via* inhibition (Wen et al., 2024), explaining the aggressive phenotypes observed in our functional assays. Clinically, our integrative study delivers significant advances for ESCA management. The nomogram achieves superior prognostic discrimination, outperforming conventional TNM staging and offering refined risk stratification. Beyond prognostic value, this axis demonstrates translational potential as a liquid biopsy target: circulating miR-15b-5p levels correlate with chemotherapy resistance in ESCC patients (Li et al., 2017), suggesting utility for monitoring therapeutic response. Mechanistically, targeting this pathway holds therapeutic promise given *BTG2*’s dual role in stabilizing p53 tumor-suppressor activity and antagonizing oncogenic Wnt/*β*-catenin signaling (Tsui et al., 2018; Gao, Yang & Wang, 2016), which may sensitize tumors to existing targeted therapies.

Our study has several limitations. The reliance on TCGA data may underrepresent the molecular heterogeneity between ESCC and EAC subtypes, which could influence the context-specific roles of this axis. Although the miR-15b-5p/*BTG2* axis was dysregulated in both subtypes, future studies with balanced cohorts are needed to confirm its broad relevance. Furthermore, our functional validation was confined to *in vitro* models. Assessing the therapeutic potential of this axis therefore requires future *in vivo* studies. Finally, while our prognostic nomogram showed promising accuracy, its clinical utility needs validation in prospective, multi-center trials using standardized assays, such as liquid biopsy, before clinical application can be considered. Until then, the nomogram should serve as a conceptual framework for risk stratification rather than a definitive clinical tool. Moreover, the detailed mechanisms of *BTG2* in ESCA also warrant further exploration. Recent work in lung adenocarcinoma revealed *BTG2* interaction with HIF-1*α* to regulate glycolysis (Zhang et al., 2020), suggesting a conserved metabolic role worth exploring in ESCA. Furthermore, exploring *BTG2* interplay with epigenetic modifiers such as *DNMT3A*, recently implicated in silencing tumor suppressors in ESCC (Xiao et al., 2024), could uncover novel mechanisms of ESCA progression.

Collectively, our study provides robust evidence that the miR-15b-5p/*BTG2* regulatory axis functions as a key driver of esophageal cancer progression through epigenetic dysregulation and tumor-suppressor silencing. The integration of multi-omics profiling with functional validation establishes this axis not only as a promising prognostic biomarker—surpassing conventional staging systems in predictive accuracy-but also unveils its therapeutic potential for targeted intervention. While further validation in diverse clinical cohorts and experimental models is warranted, these findings fundamentally advance our understanding of miRNA-mediated oncogenesis in ESCA and lay a concrete foundation for developing miRNA-based liquid biopsies and combination therapies targeting this novel pathway.

##  Supplemental Information

10.7717/peerj.20538/supp-1 Supplemental Information 1 Raw data and R code All the raw data, result images and running codes in this paper, including qRT-PCR data and cell behavior measurements.

10.7717/peerj.20538/supp-2 Supplemental Information 2 Codebook

10.7717/peerj.20538/supp-3 Supplemental Information 3 The complete analysis workflow

10.7717/peerj.20538/supp-4 Supplemental Information 4 Kaplan-Meier curve of 11 prognostic miRNAs in ESCA

10.7717/peerj.20538/supp-5 Supplemental Information 5 LASSO-Cox regression analysis of 271 candidate mRNAs

10.7717/peerj.20538/supp-6 Supplemental Information 6 (A) miR-15b-5p is significantly upregulated in ESCC samples from the GSE13937 dataset (B) miR-15b-5p is significantly upregulated in EAC samples from the GSE13937 dataset. (C) Calibration curve for the miR-15b-5p nomogram, illustrating the concordance between predicted and actual 1-, 3- and 5-year overall survival. (D) Calibration curve for the *BTG2* nomogram, demonstrating the model’s predictive accuracy.

10.7717/peerj.20538/supp-7 Supplemental Information 7 Clinicopathological information of TCGA-ESCA datasets

10.7717/peerj.20538/supp-8 Supplemental Information 8 Target genes prediction of differentially expressed miRNAs by FunRich

10.7717/peerj.20538/supp-9 Supplemental Information 9 MIQE checklist

## Abbreviations

ESCA: Esophageal cancer
LASSO: Least absolute shrinkage and selection operator
WGCNA: weighted gene co-expression network analysis
RT-PCR: quantitative real-time polymerase chain reaction
OS: overall survival
ROC: receiver operator characteristic curve
AUC: Area Under Curve
KNN: K-Nearest Neighbor
GEO: Gene Expression Omnibus
GO: Gene Ontology
KEGG: Kyoto Encyclopedia of Genes and Genomes
FC: fold change
FDR: False Discovery Rate
BP: biological process
MF: molecular function
CC: cellular component
DEGs: differential expression genes
TCGA: The Cancer Genome Atlas
miRNAs: MicroRNAs
ESCC: Esophageal squamous cell carcinoma
EAC: Esophageal adenocarcinoma
SD: standard deviation
*BTG2*: B-cell translocation gene 2
PDX: patient-derived xenograft

## Additional Information and Declarations

### Data Availability

The following information was supplied regarding data availability: The raw measurements are available in the Supplemental Files.
