# Supplementary material for: Integrative analysis for identification of key miRNA-mRNA regulatory axes in esophageal cancer and preliminary validation of the regulatory role of miR-15b-5p/BTG2 therein
Source: PeerJ. 2026 Jan 28;14:e20538. doi: 10.7717/peerj.20538 (PMC12860276; doi:10.7717/peerj.20538)
Supplement: Supplemental Information 1 — All the raw data, result images and running codes in this paper, including qRT-PCR data and cell behavior measurements. [file peerj-14-20538-s001.zip › Supplementary files 1/result 3/EnrichGOBPCircleplot.pdf]

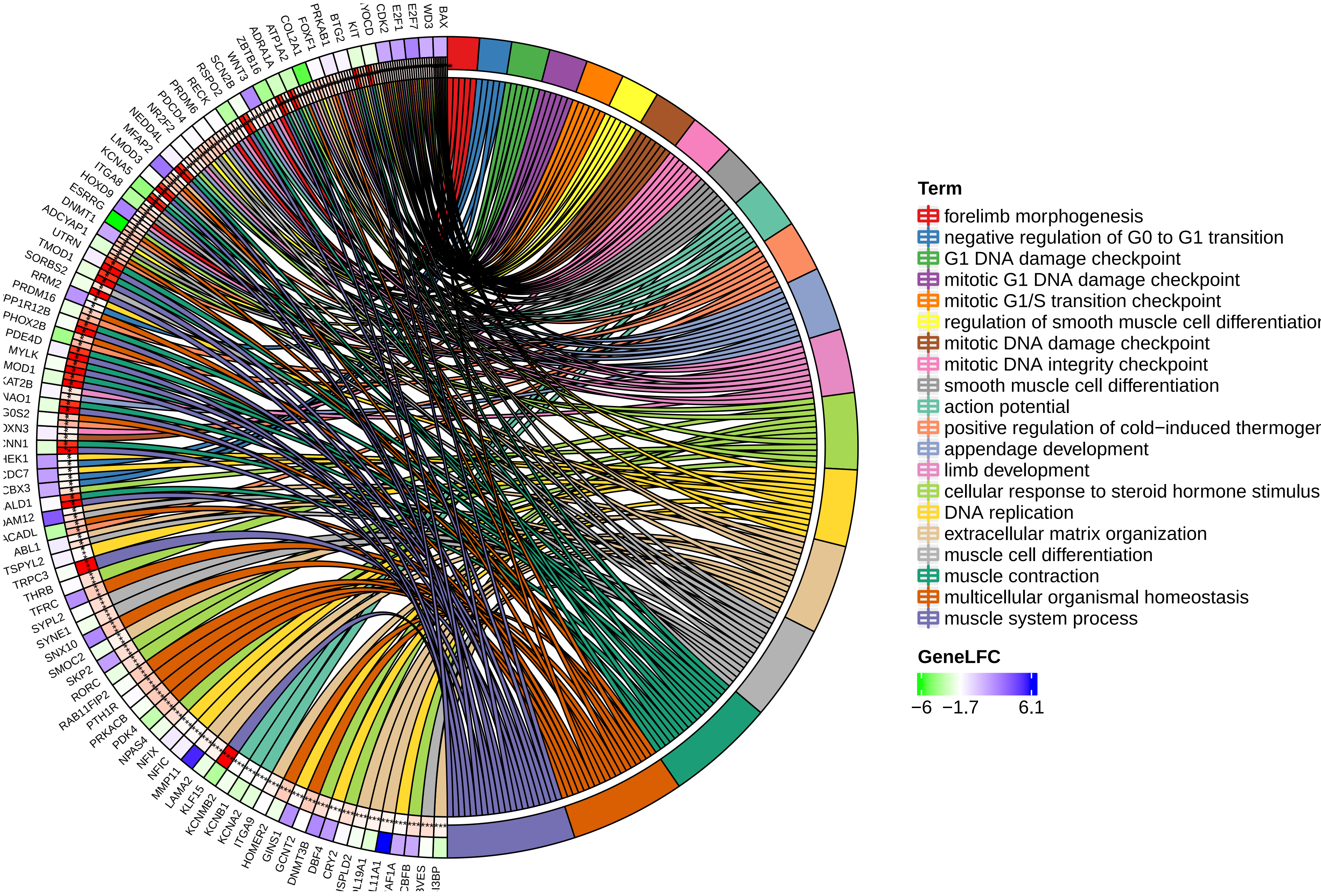
Term
forelimb morphogenesis
negative regulation of G0 to G1 transition
G1 DNA damage checkpoint
mitotic G1 DNA damage checkpoint
mitotic G1/S transition checkpoint
regulation of smooth muscle cell
mitotic DNA damage checkpoint
mitotic DNA integrity checkpoint
smooth muscle cell differentiation
action potential
positive regulation of cold−induced
appendage development
limb development
cellular response to steroid hormone stimulus
DNA replication
extracellular matrix organization
muscle cell differentiation
muscle contraction
multicellular organismal homeostasis
muscle system process
GeneLFC
−6 −1.7 6.1
BAX
E2F7
E2F1
CDK2
KIT
BTG2
PRKAB1
FOXF1
COL2A1
ATP1A2
ADRA1A
ZBTB16
WNT3
SCN2B
RSPO2
RECK
PRDM6
PDCD4
NR2F2
NEDD4L
MFAP2
LMOD3
KCNA5
ITGA8
HOXD9
ESRRG
DNMT1
ADCYAP1
UTRN
TMOD1
SORBS2
RRM2
PRDM16
PHOX2B
PDE4D
MYLK
ABL1
TSPYL2
TRPC3
THRB
TFRC
SYPL2
SYNE1
SNX10
SMOC2
SKP2
RORC
RAB11FIP2
PTH1R
PRKACB
PDK4
NPAS4
NFIX
NFIC
MMP11
LAMA2
KLF15
KCNMB2
KCNB1
KCNA2
ITGA9
HOMER2
GINS1
GCNT2
DNMT3B
DBF4
CRY2
