# Supplementary material for: Integrative analysis for identification of key miRNA-mRNA regulatory axes in esophageal cancer and preliminary validation of the regulatory role of miR-15b-5p/BTG2 therein
Source: PeerJ. 2026 Jan 28;14:e20538. doi: 10.7717/peerj.20538 (PMC12860276; doi:10.7717/peerj.20538)
Supplement: Supplemental Information 1 — All the raw data, result images and running codes in this paper, including qRT-PCR data and cell behavior measurements. [file peerj-14-20538-s001.zip › Supplementary files 1/result 7/qinxi2.pdf]

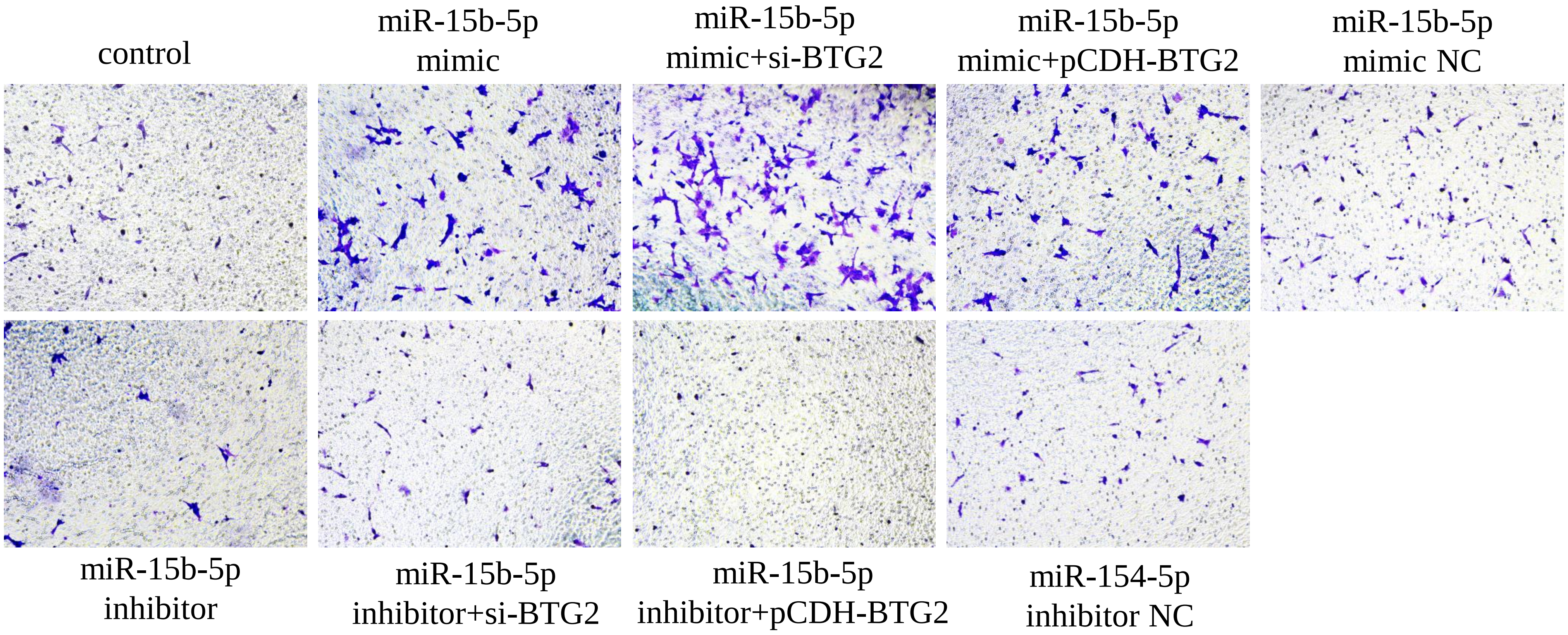
control
miR-15b-5p mimic
miR-15b-5p mimic+si-BTG2
miR-15b-5p mimic+pCDH-BTG2
miR-15b-5p mimic NC
miR-15b-5p inhibitor
miR-15b-5p inhibitor+si-BTG2
miR-15b-5p inhibitor+pCDH-BTG2
miR-154-5p inhibitor NC
