# Supplementary figures and images for: Integrative analysis for identification of key miRNA-mRNA regulatory axes in esophageal cancer and preliminary validation of the regulatory role of miR-15b-5p/BTG2 therein
Source: PeerJ. 2026 Jan 28;14:e20538. doi: 10.7717/peerj.20538 (PMC12860276; doi:10.7717/peerj.20538)

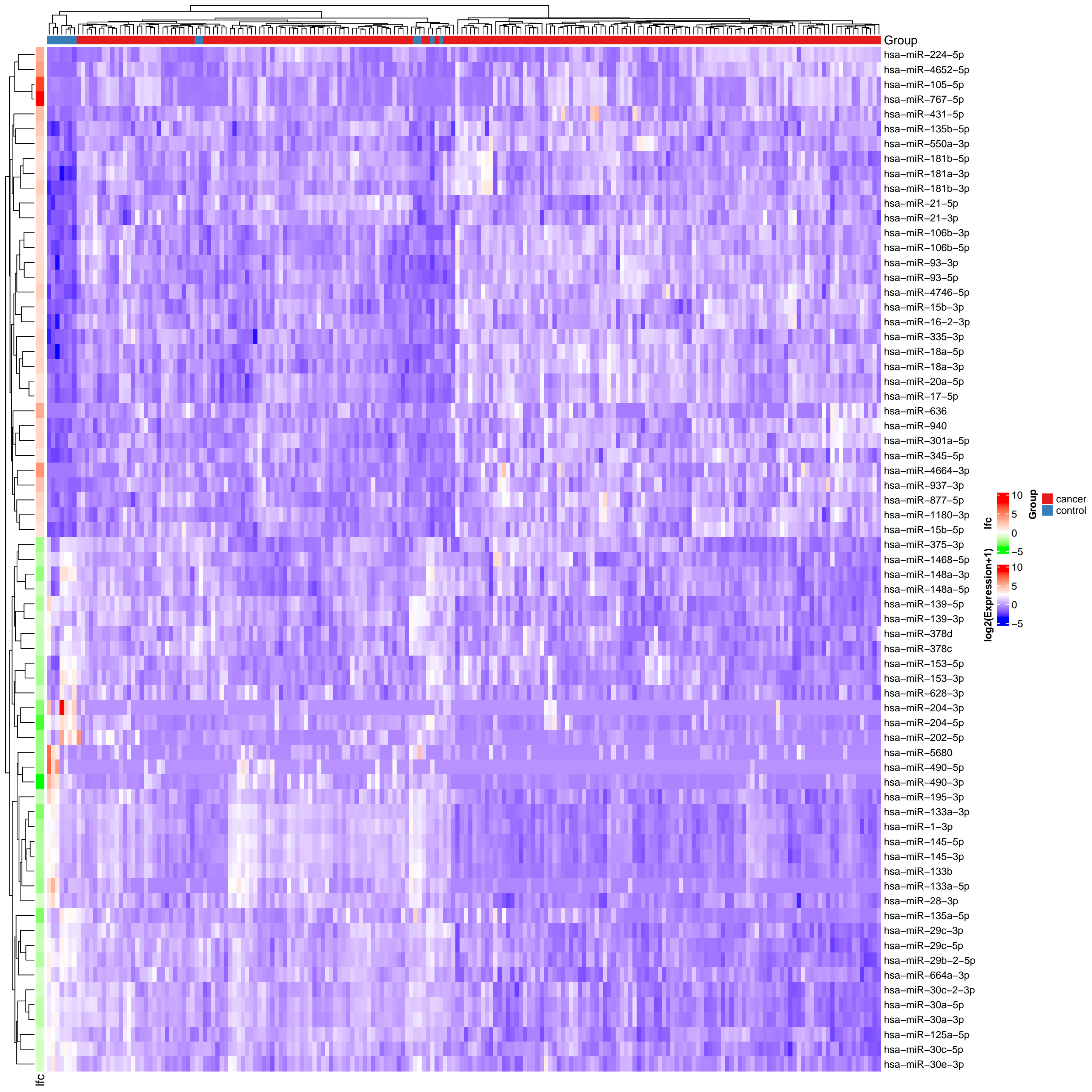

Supplement: Supplemental Information 1 — All the raw data, result images and running codes in this paper, including qRT-PCR data and cell behavior measurements. [file peerj-14-20538-s001.zip › Supplementary files 1/result 1/miRNA_hetmap_cancer-vs-control.pdf]

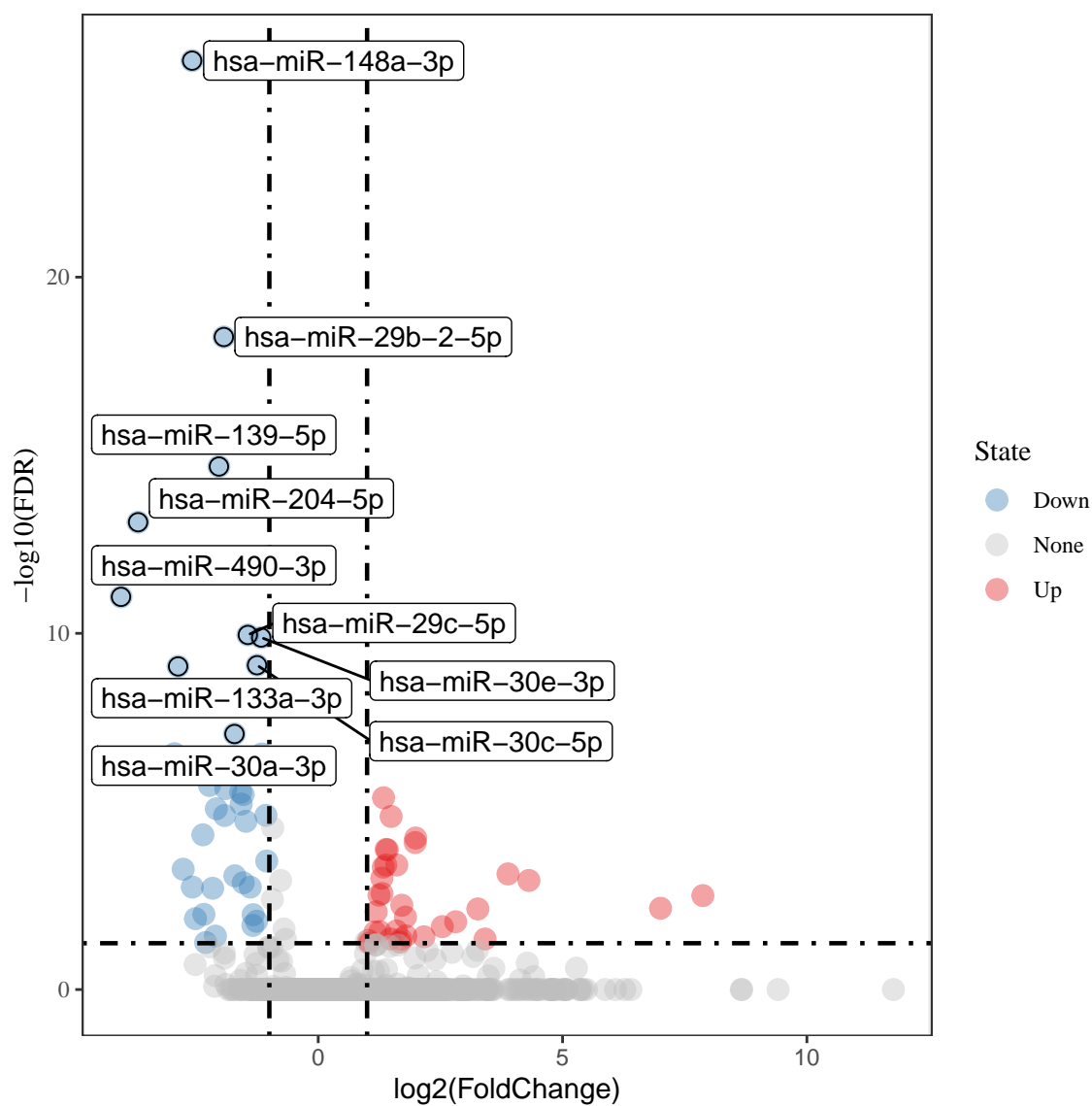

Supplement: Supplemental Information 1 — All the raw data, result images and running codes in this paper, including qRT-PCR data and cell behavior measurements. [file peerj-14-20538-s001.zip › Supplementary files 1/result 1/miRNA_volcano_cancer-vs-control.pdf]

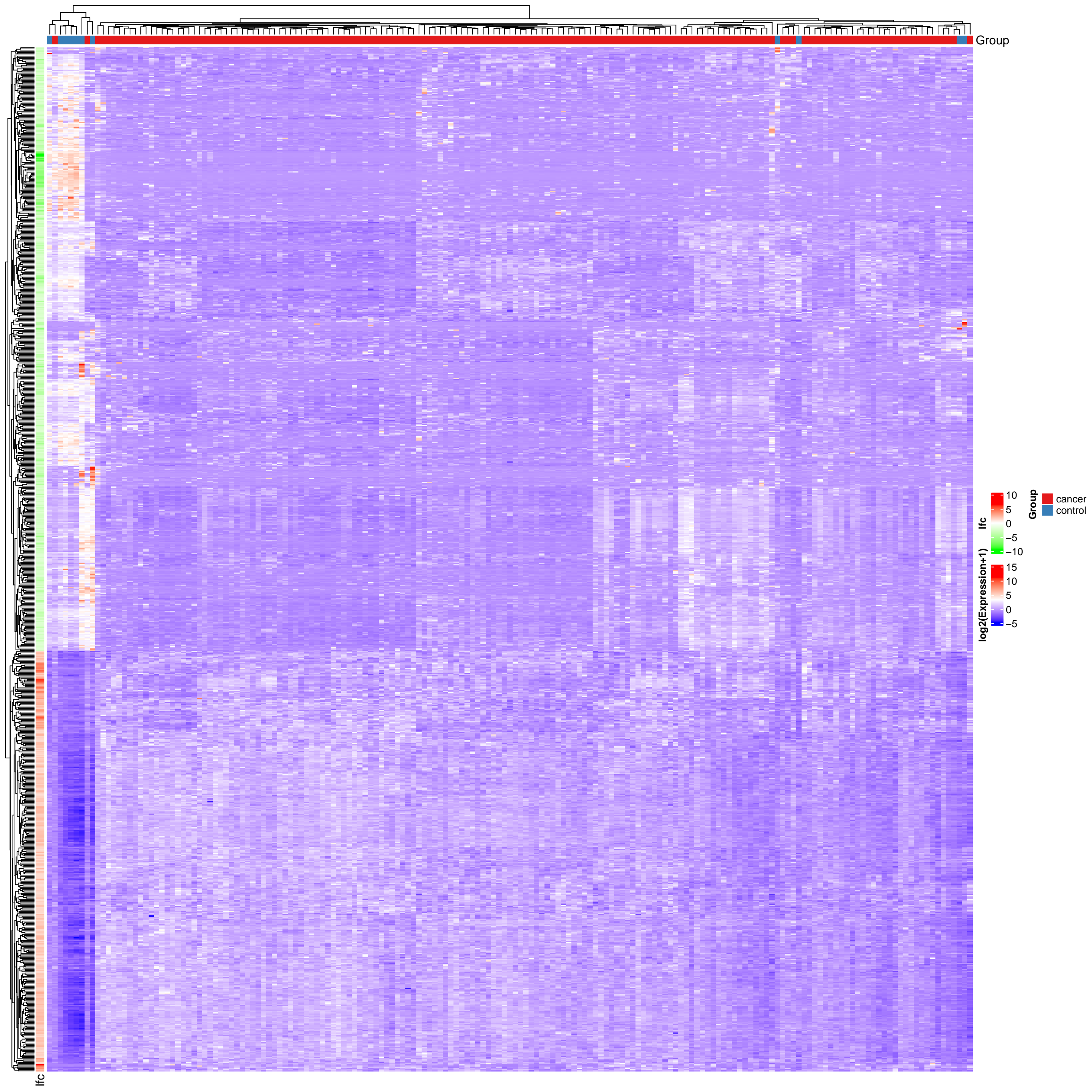

Supplement: Supplemental Information 1 — All the raw data, result images and running codes in this paper, including qRT-PCR data and cell behavior measurements. [file peerj-14-20538-s001.zip › Supplementary files 1/result 1/mRNA_hetmap_cancer-vs-control.pdf]

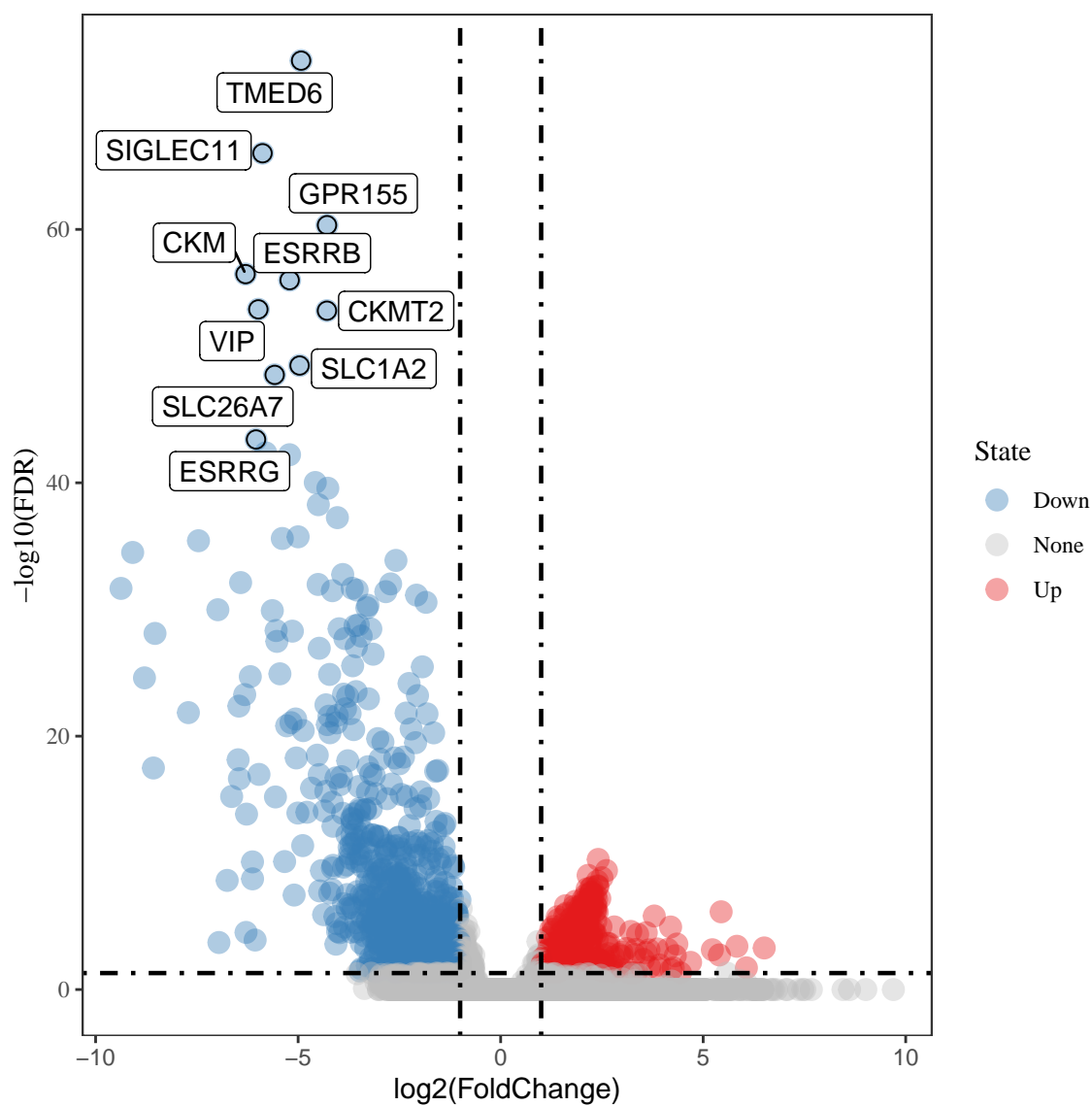

Supplement: Supplemental Information 1 — All the raw data, result images and running codes in this paper, including qRT-PCR data and cell behavior measurements. [file peerj-14-20538-s001.zip › Supplementary files 1/result 1/mRNA_volcano_cancer-vs-control.pdf]

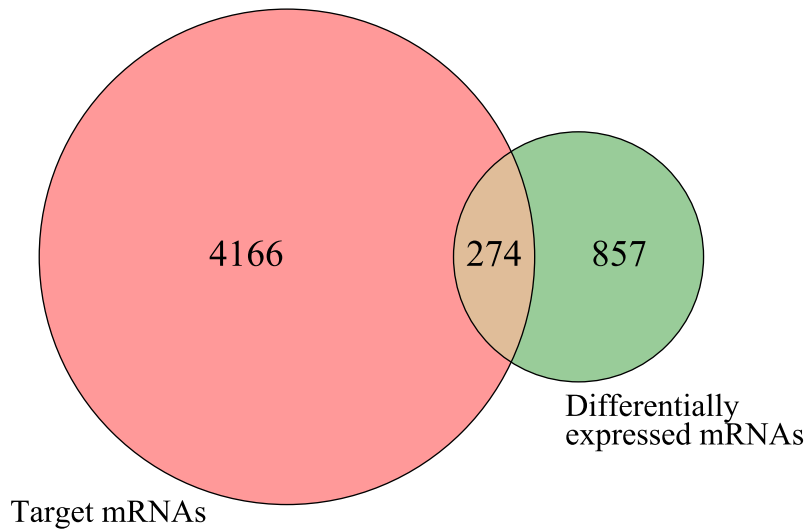

Supplement: Supplemental Information 1 — All the raw data, result images and running codes in this paper, including qRT-PCR data and cell behavior measurements. [file peerj-14-20538-s001.zip › Supplementary files 1/result 2/common mRNAs.pdf]

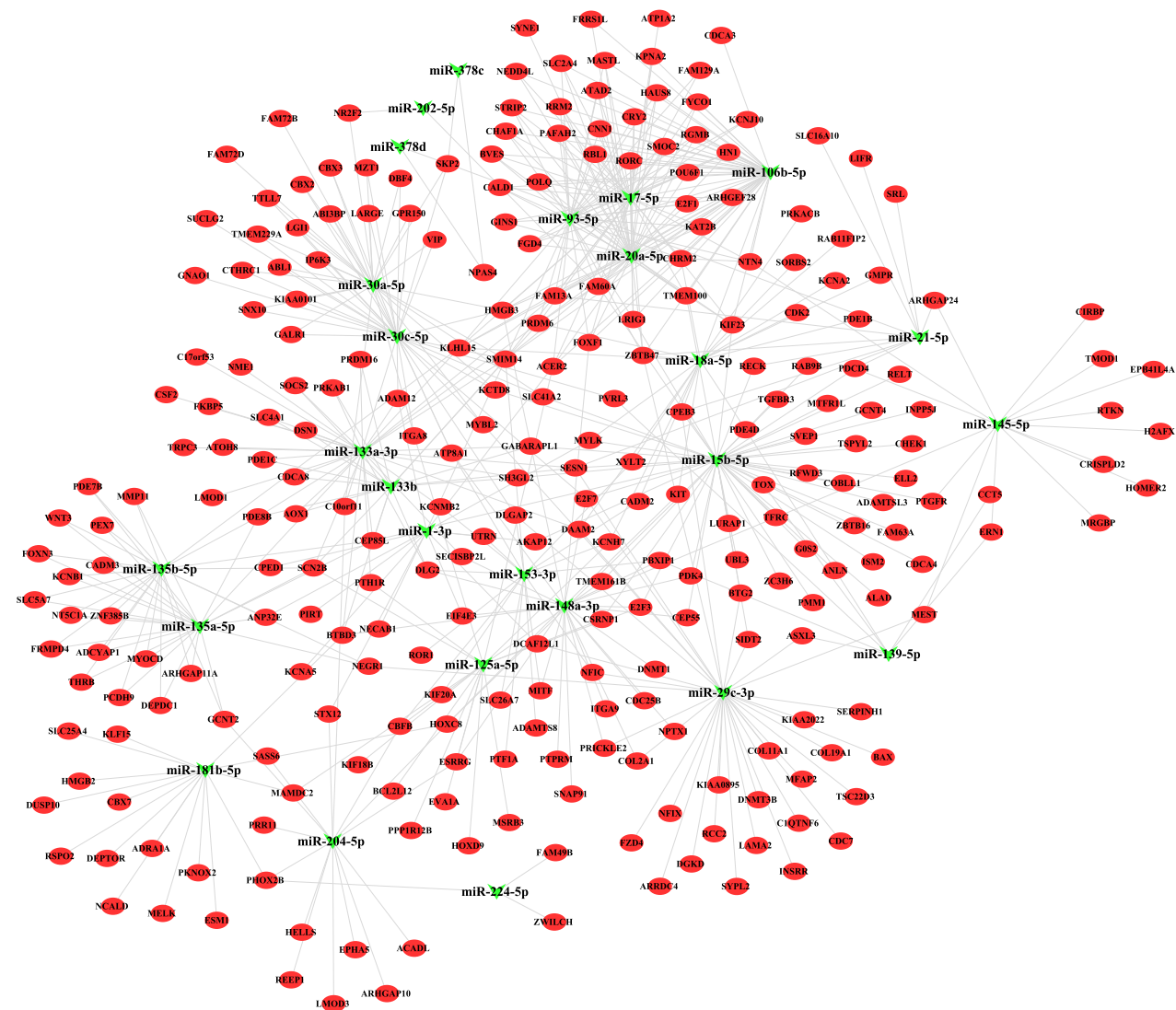

Supplement: Supplemental Information 1 — All the raw data, result images and running codes in this paper, including qRT-PCR data and cell behavior measurements. [file peerj-14-20538-s001.zip › Supplementary files 1/result 2/edges.txt.pdf]

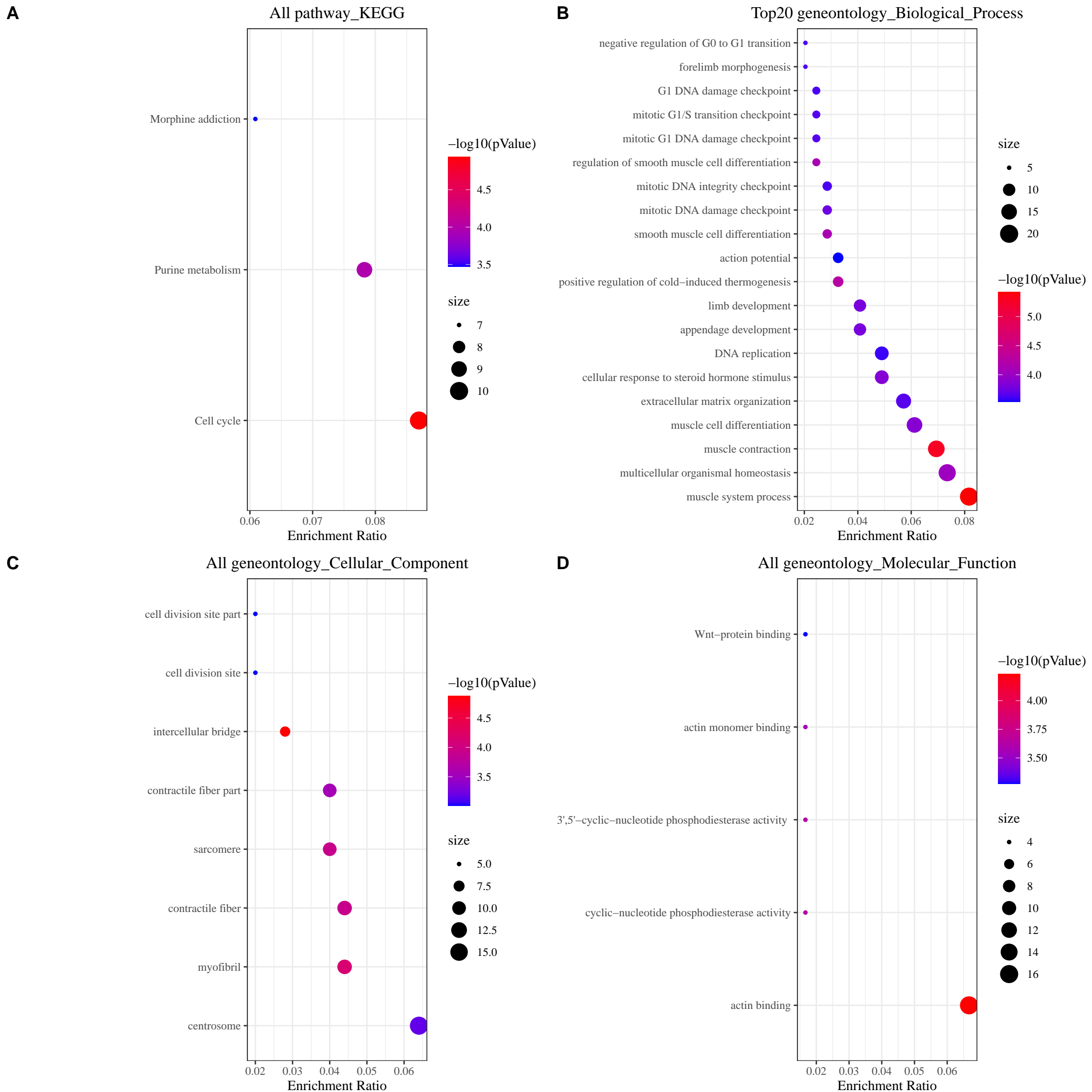

Supplement: Supplemental Information 1 — All the raw data, result images and running codes in this paper, including qRT-PCR data and cell behavior measurements. [file peerj-14-20538-s001.zip › Supplementary files 1/result 3/EnrichDotplot.pdf]

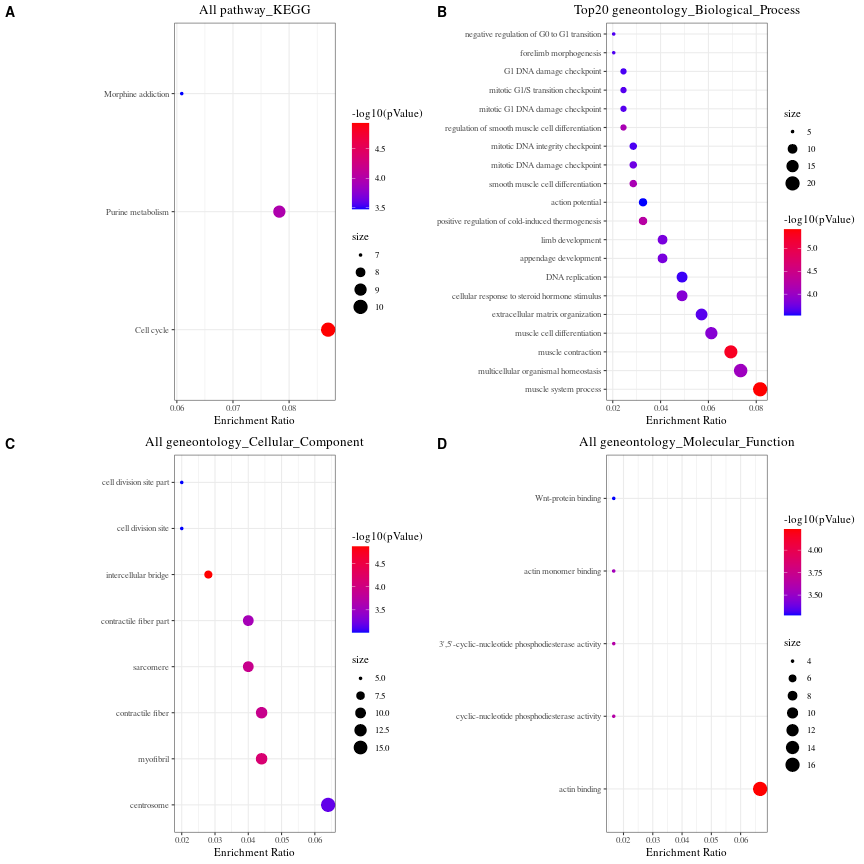

Supplement: Supplemental Information 1 — All the raw data, result images and running codes in this paper, including qRT-PCR data and cell behavior measurements. [file peerj-14-20538-s001.zip › Supplementary files 1/result 3/EnrichDotplot.png]

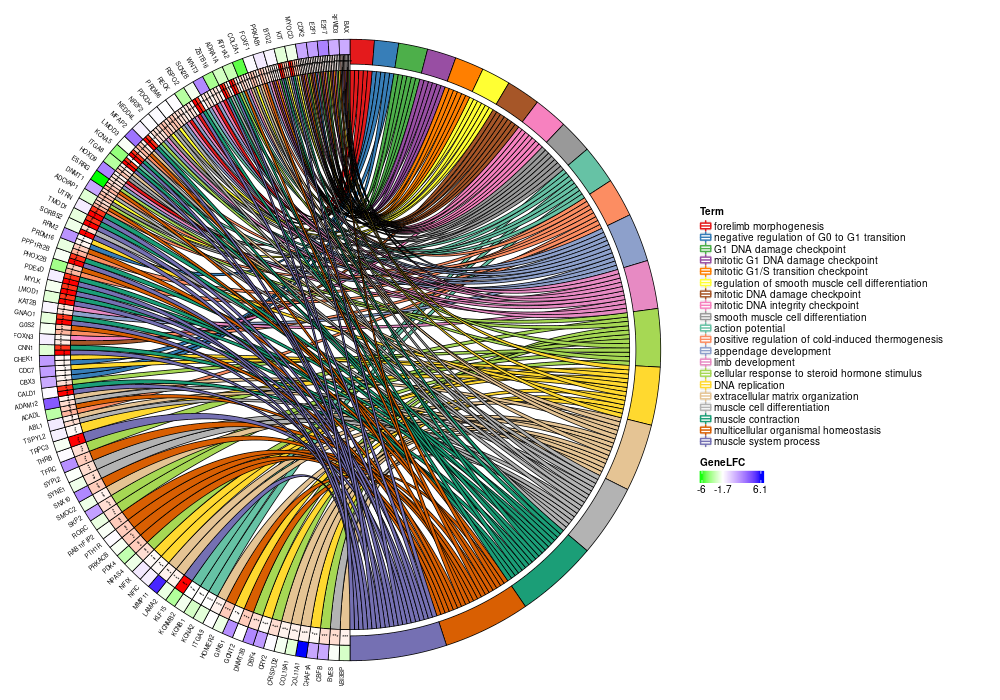

Supplement: Supplemental Information 1 — All the raw data, result images and running codes in this paper, including qRT-PCR data and cell behavior measurements. [file peerj-14-20538-s001.zip › Supplementary files 1/result 3/EnrichGOBPCircleplot.png]

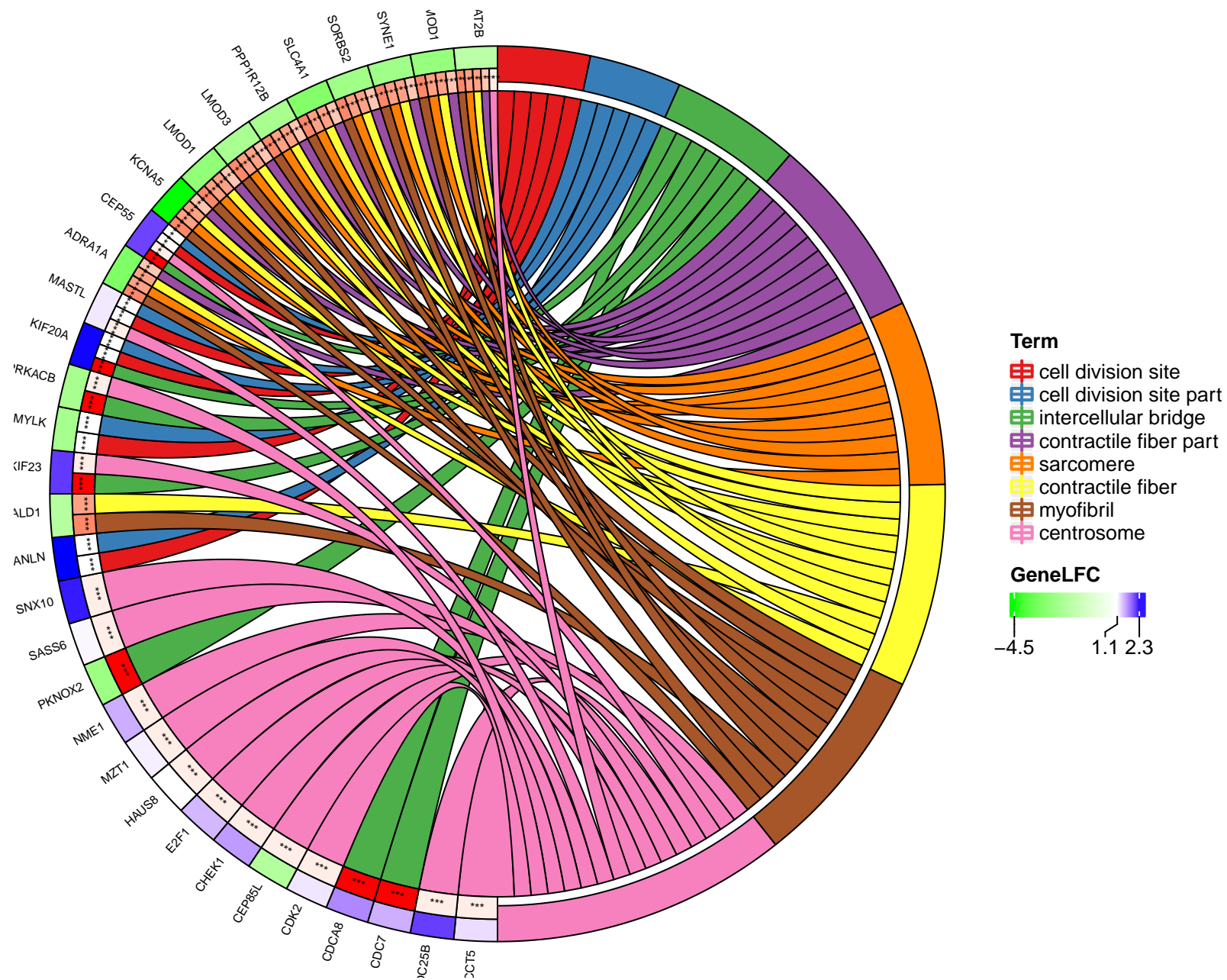

Supplement: Supplemental Information 1 — All the raw data, result images and running codes in this paper, including qRT-PCR data and cell behavior measurements. [file peerj-14-20538-s001.zip › Supplementary files 1/result 3/EnrichGOCCCircleplot.pdf]

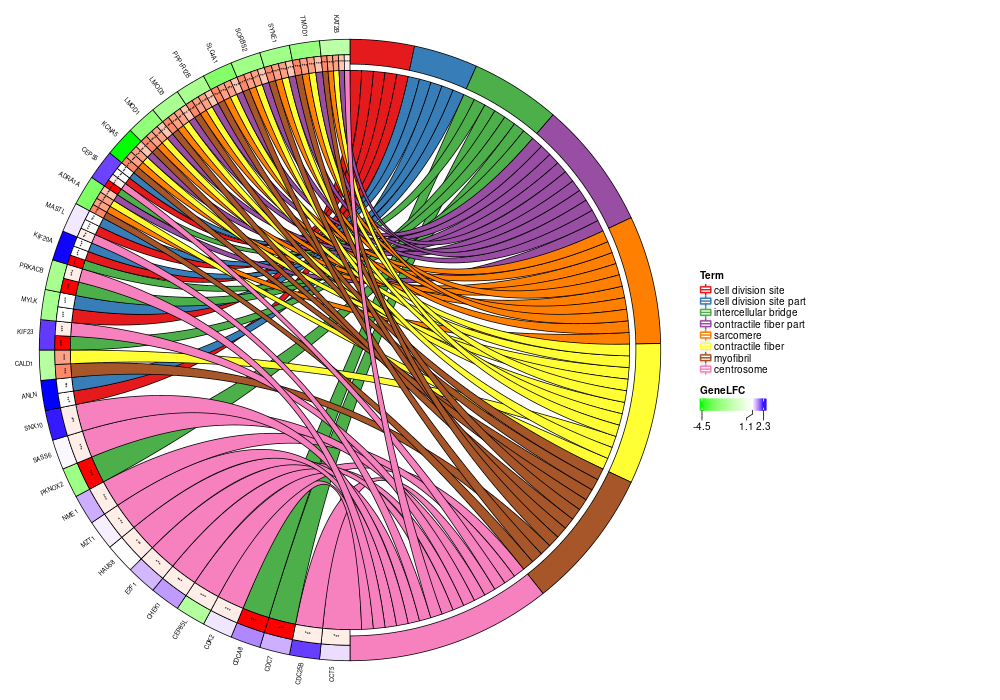

Supplement: Supplemental Information 1 — All the raw data, result images and running codes in this paper, including qRT-PCR data and cell behavior measurements. [file peerj-14-20538-s001.zip › Supplementary files 1/result 3/EnrichGOCCCircleplot.png]

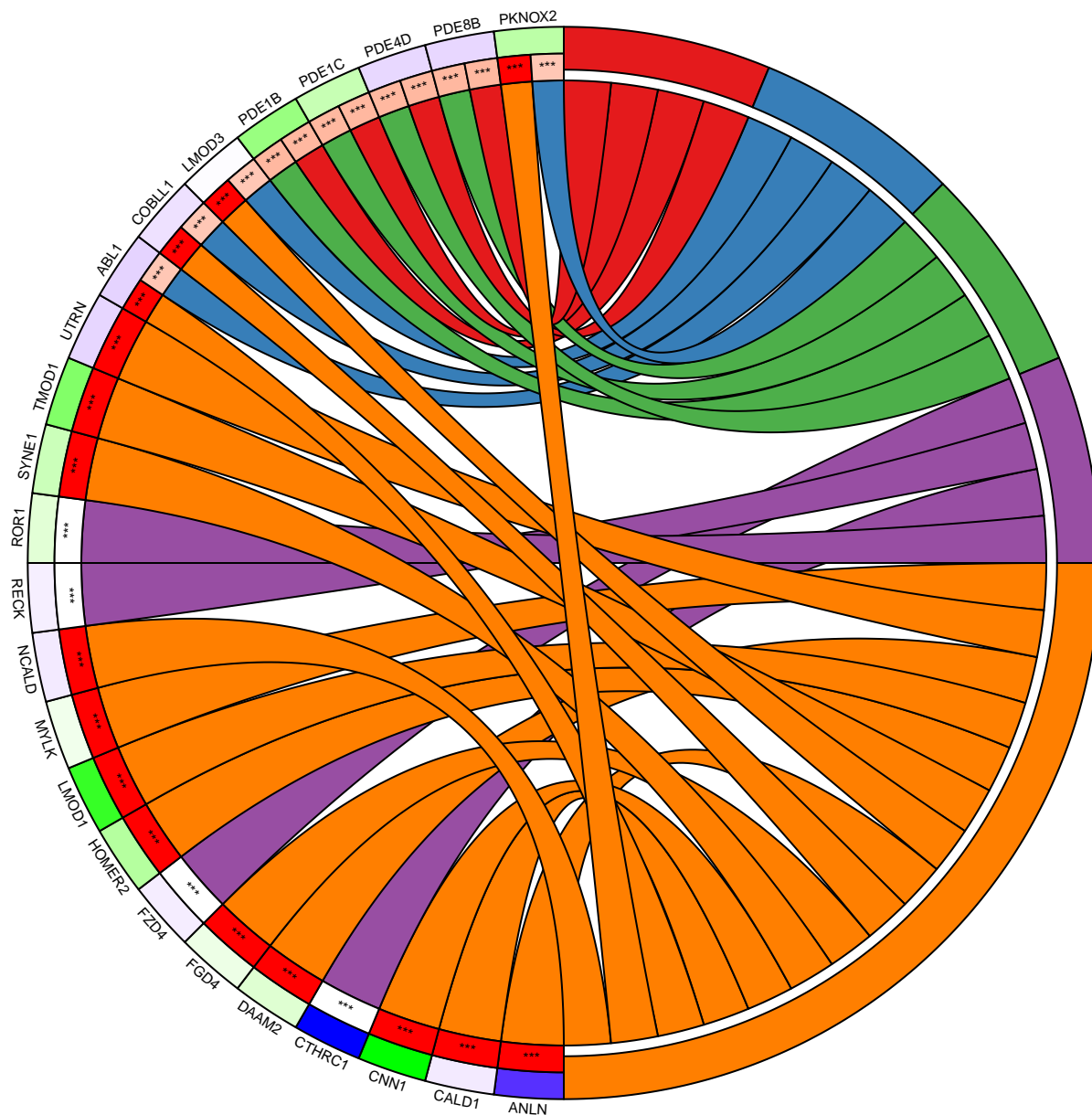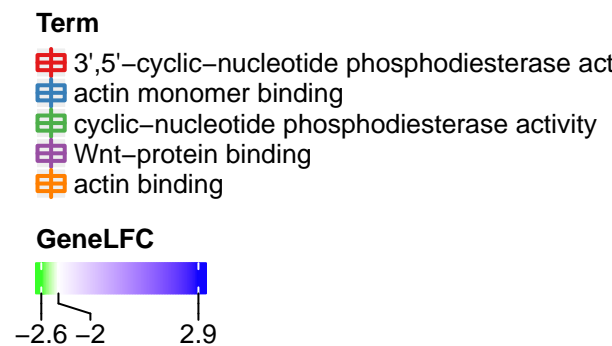

Supplement: Supplemental Information 1 — All the raw data, result images and running codes in this paper, including qRT-PCR data and cell behavior measurements. [file peerj-14-20538-s001.zip › Supplementary files 1/result 3/EnrichGOMFCircleplot.pdf]

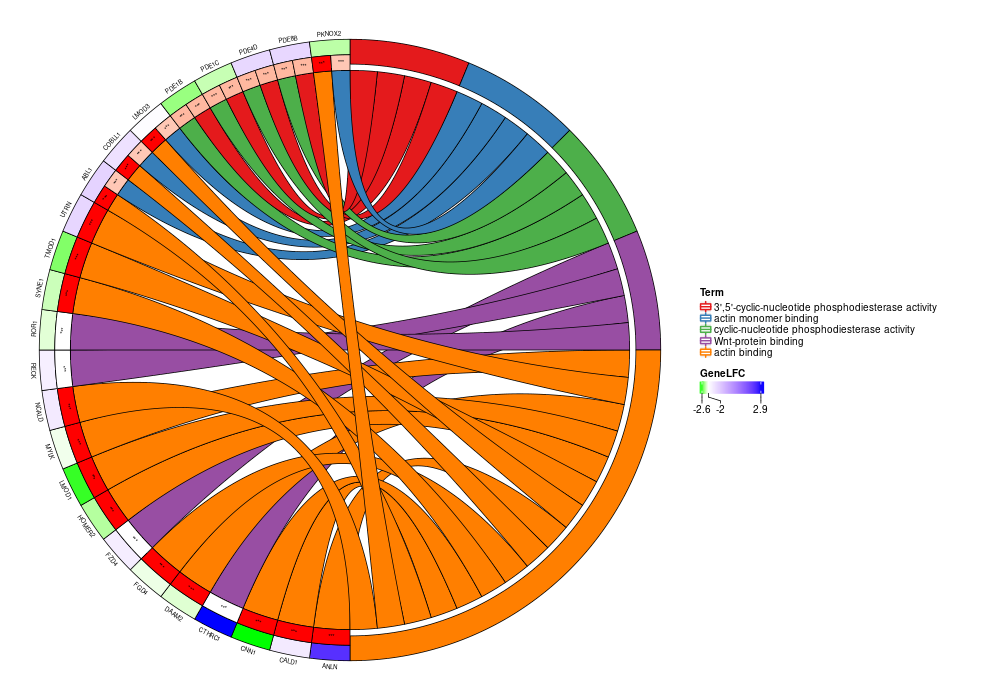

Supplement: Supplemental Information 1 — All the raw data, result images and running codes in this paper, including qRT-PCR data and cell behavior measurements. [file peerj-14-20538-s001.zip › Supplementary files 1/result 3/EnrichGOMFCircleplot.png]

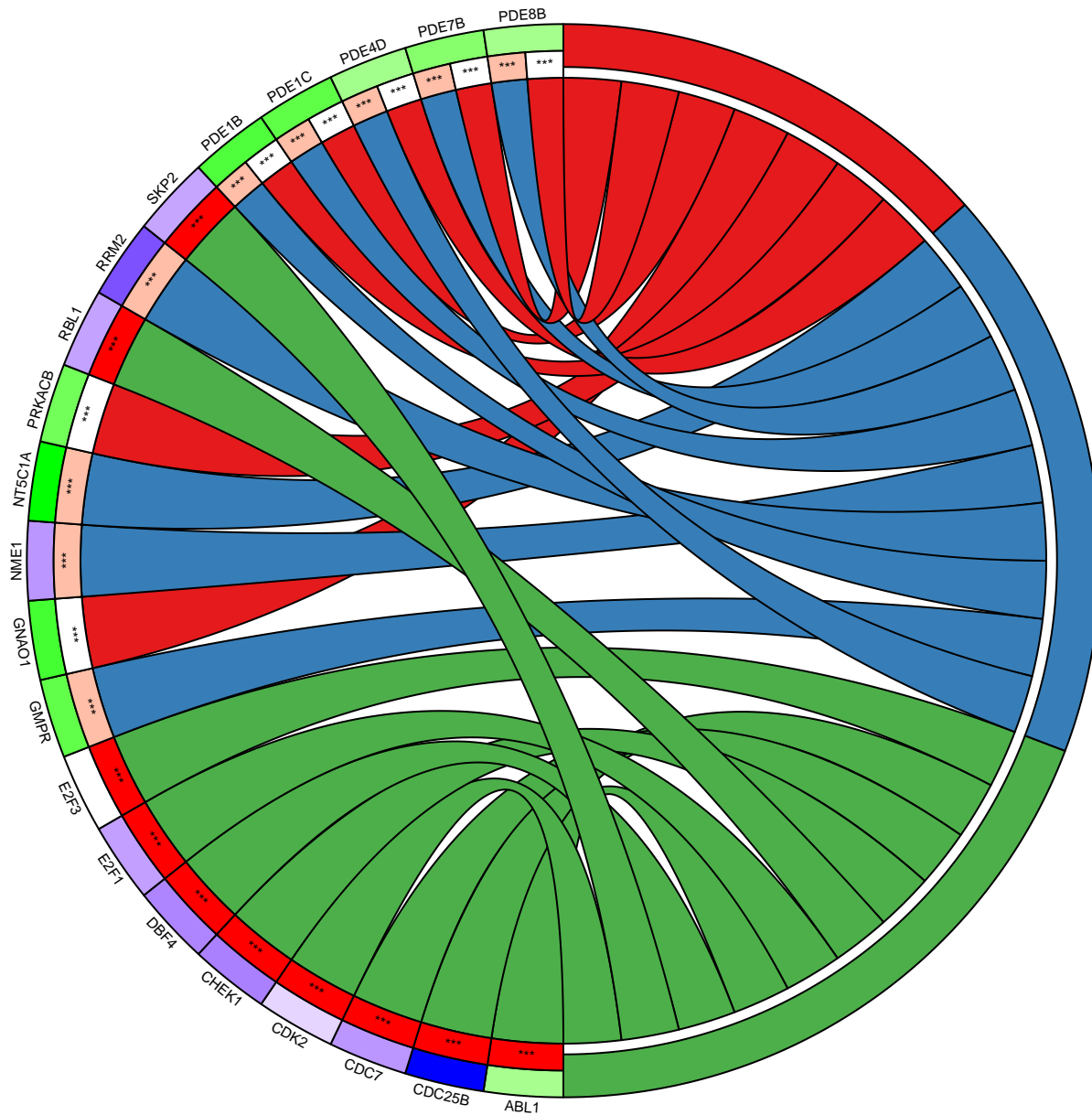

Supplement: Supplemental Information 1 — All the raw data, result images and running codes in this paper, including qRT-PCR data and cell behavior measurements. [file peerj-14-20538-s001.zip › Supplementary files 1/result 3/EnrichKEGGCircleplot.pdf]

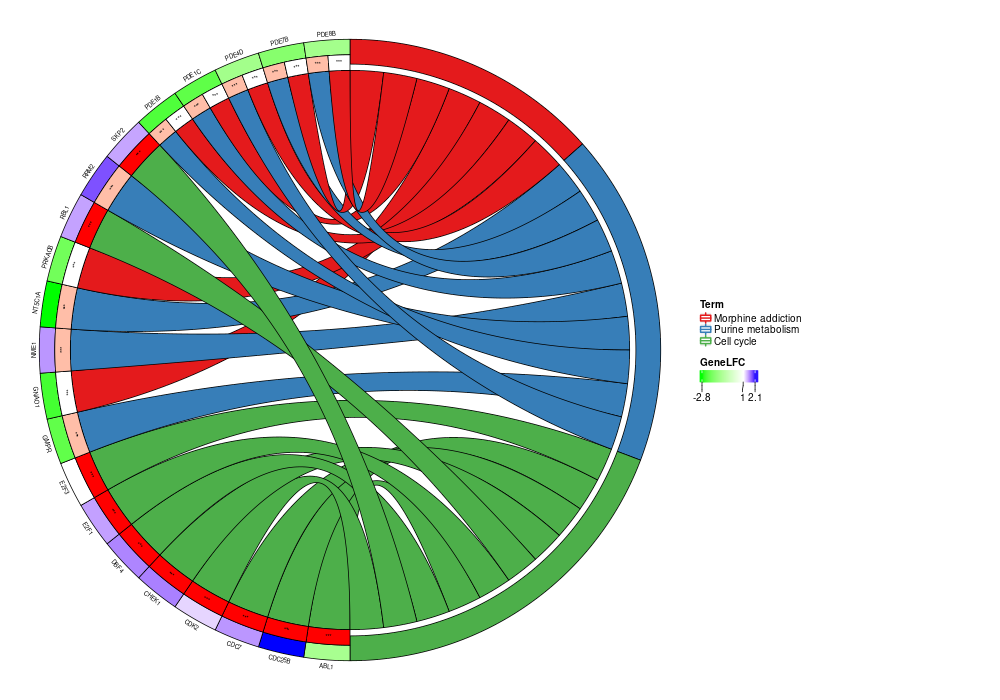

Supplement: Supplemental Information 1 — All the raw data, result images and running codes in this paper, including qRT-PCR data and cell behavior measurements. [file peerj-14-20538-s001.zip › Supplementary files 1/result 3/EnrichKEGGCircleplot.png]

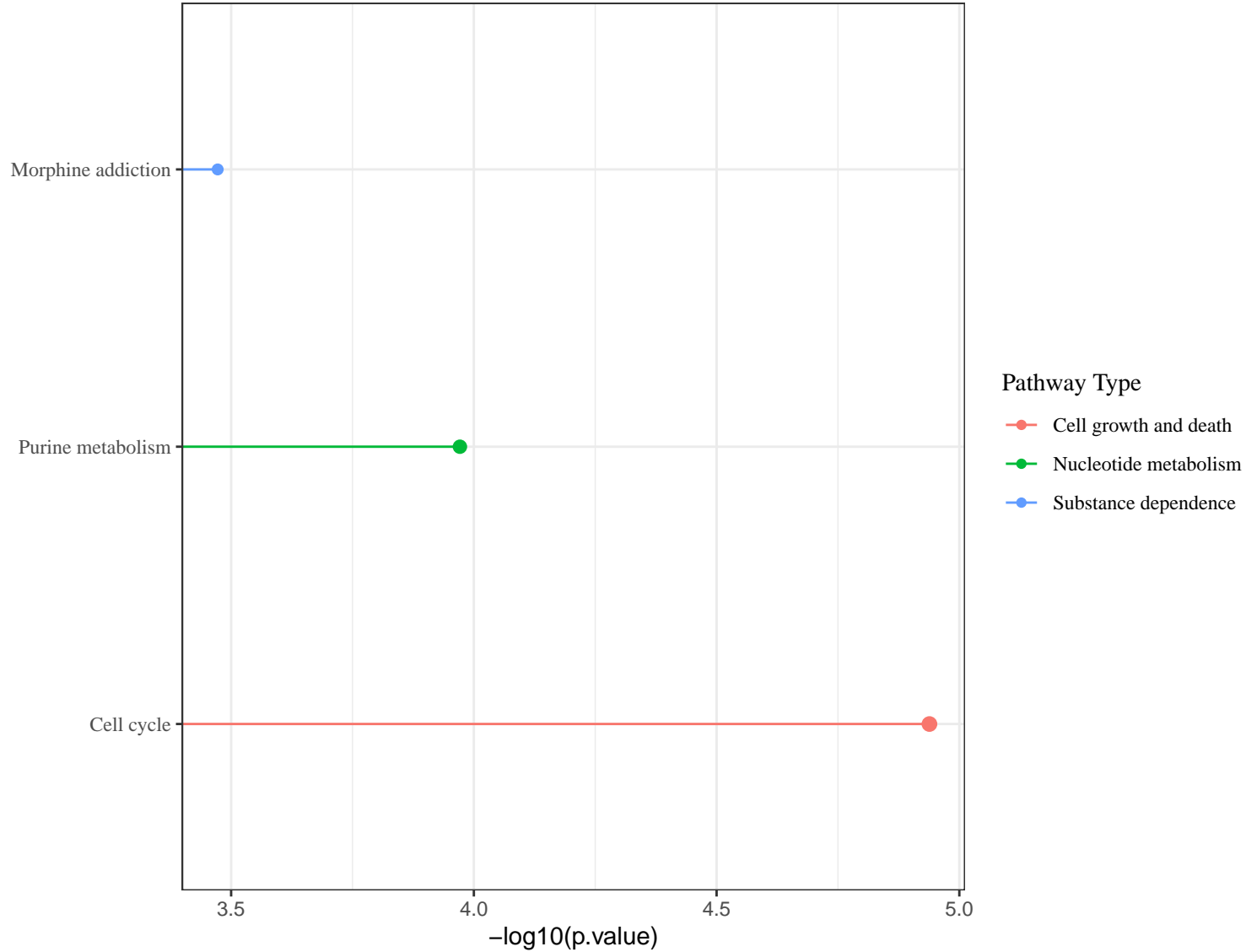

Supplement: Supplemental Information 1 — All the raw data, result images and running codes in this paper, including qRT-PCR data and cell behavior measurements. [file peerj-14-20538-s001.zip › Supplementary files 1/result 3/EnrichKEGGClassplot.pdf]

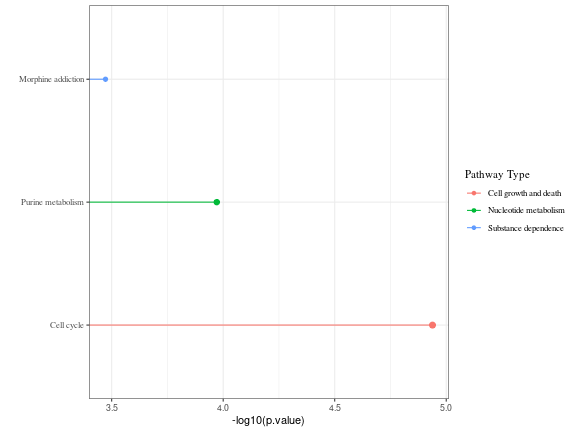

Supplement: Supplemental Information 1 — All the raw data, result images and running codes in this paper, including qRT-PCR data and cell behavior measurements. [file peerj-14-20538-s001.zip › Supplementary files 1/result 3/EnrichKEGGClassplot.png]

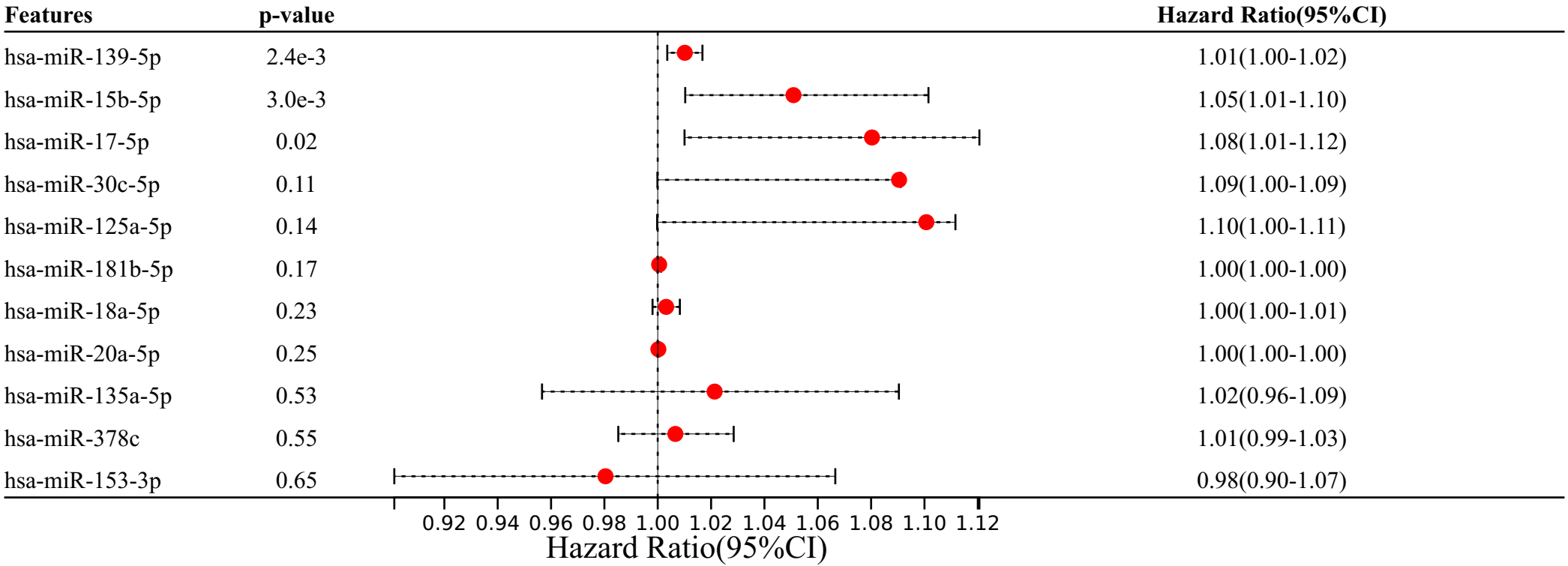

Supplement: Supplemental Information 1 — All the raw data, result images and running codes in this paper, including qRT-PCR data and cell behavior measurements. [file peerj-14-20538-s001.zip › Supplementary files 1/result 4/COX-senlintu.pdf]

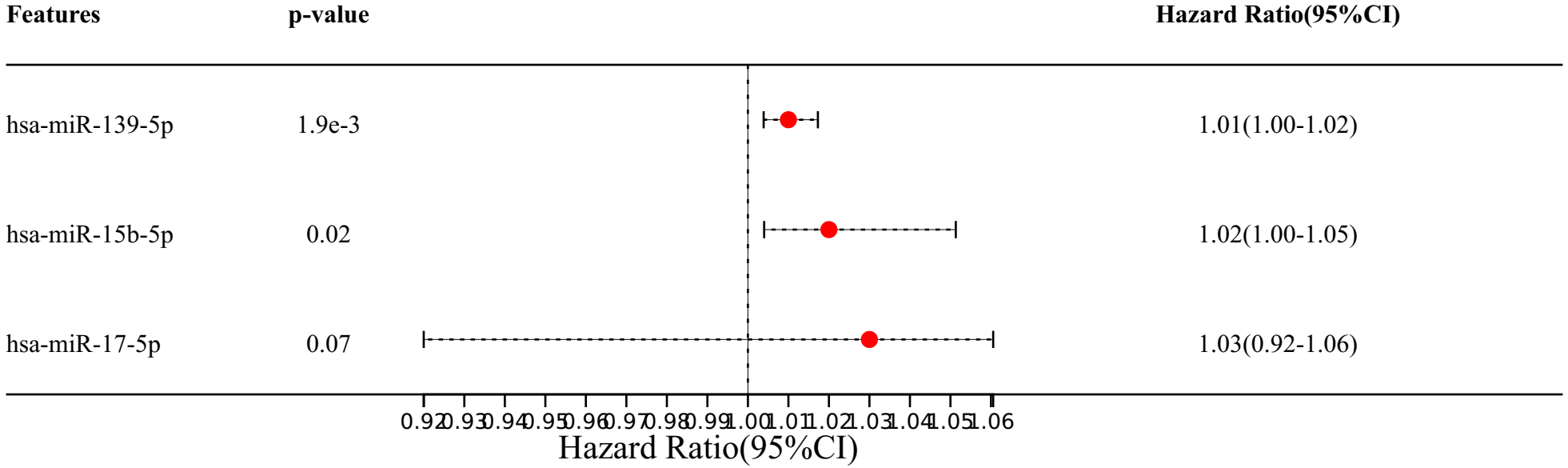

Supplement: Supplemental Information 1 — All the raw data, result images and running codes in this paper, including qRT-PCR data and cell behavior measurements. [file peerj-14-20538-s001.zip › Supplementary files 1/result 4/MUcox-miRNA.pdf]

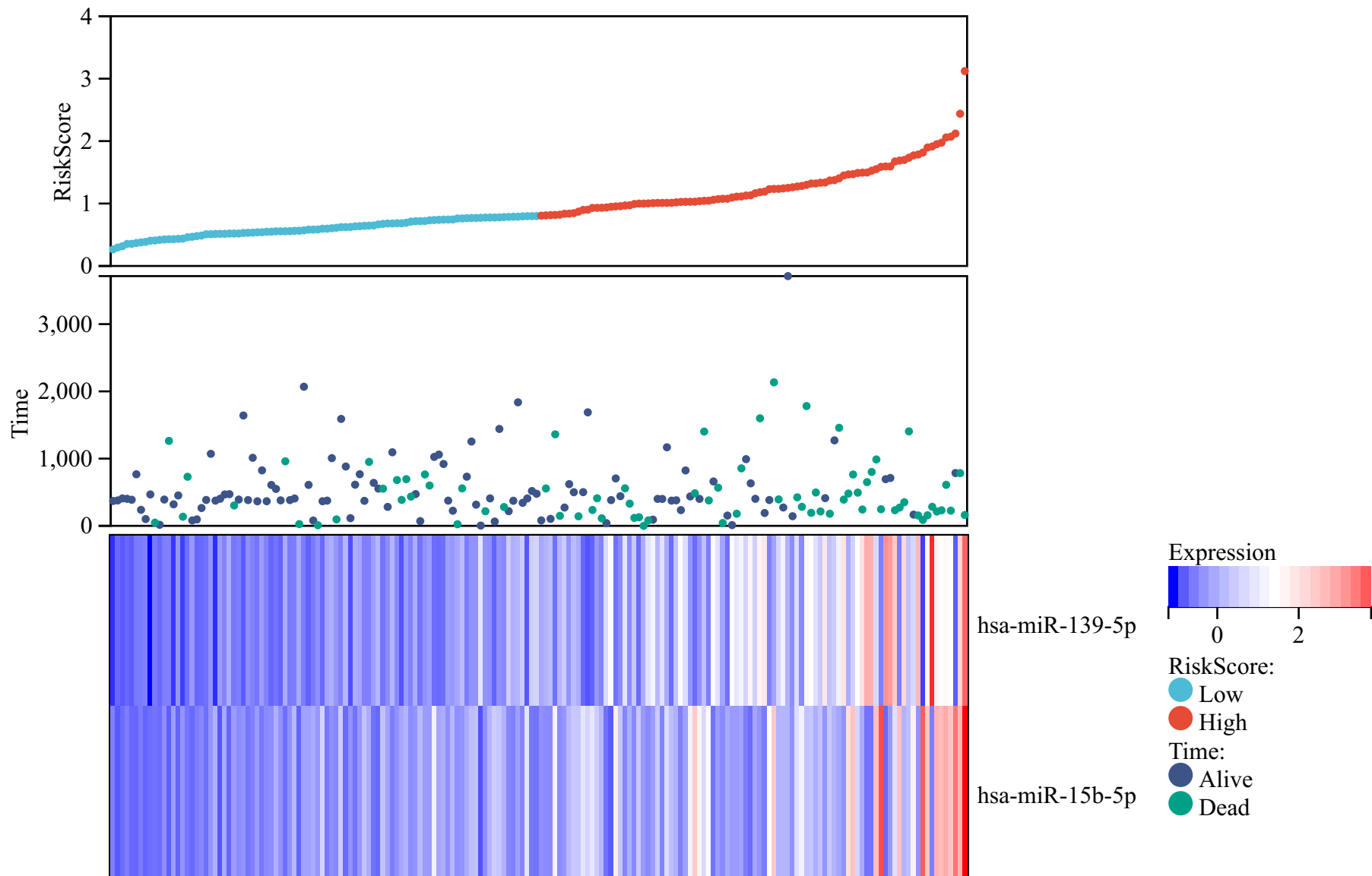

Supplement: Supplemental Information 1 — All the raw data, result images and running codes in this paper, including qRT-PCR data and cell behavior measurements. [file peerj-14-20538-s001.zip › Supplementary files 1/result 4/risk-expression-miRNA.pdf]

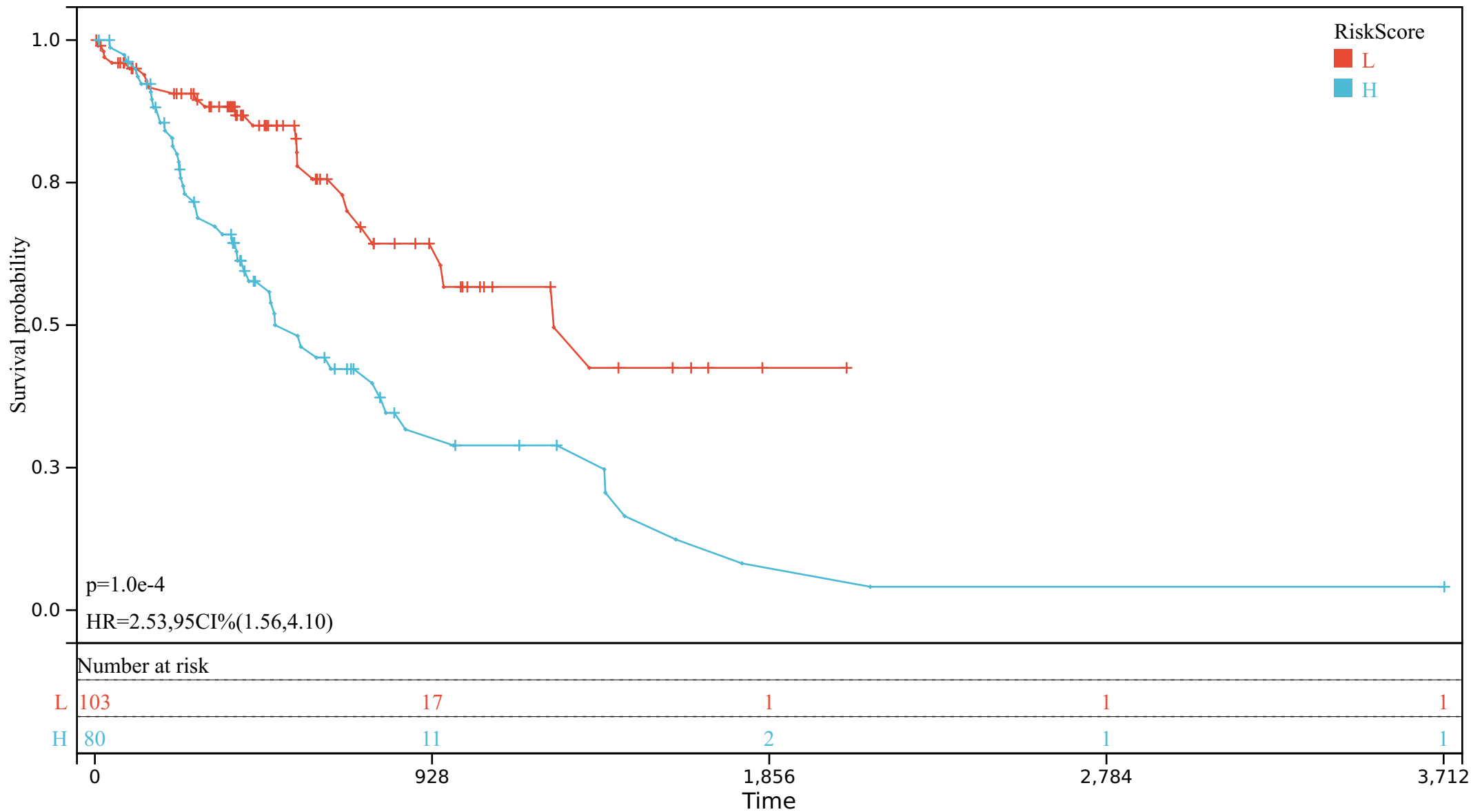

Supplement: Supplemental Information 1 — All the raw data, result images and running codes in this paper, including qRT-PCR data and cell behavior measurements. [file peerj-14-20538-s001.zip › Supplementary files 1/result 4/risk-KM.pdf]

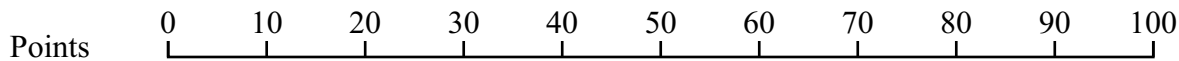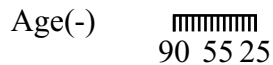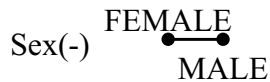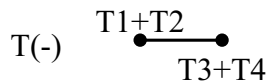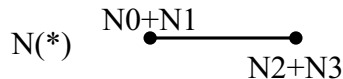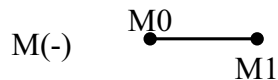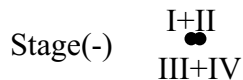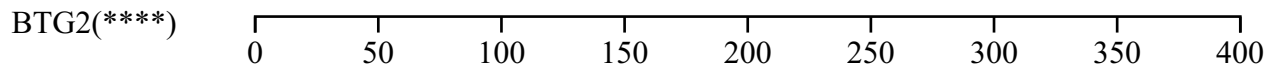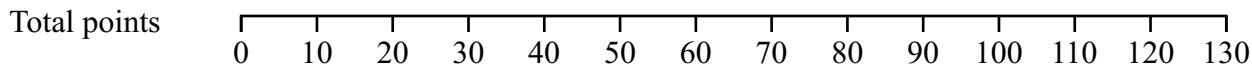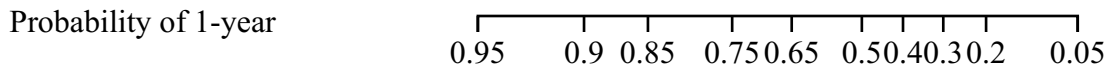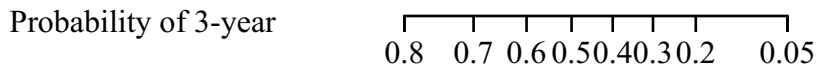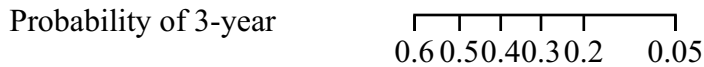

Supplement: Supplemental Information 1 — All the raw data, result images and running codes in this paper, including qRT-PCR data and cell behavior measurements. [file peerj-14-20538-s001.zip › Supplementary files 1/result 5/BTG2-nomogram.pdf]

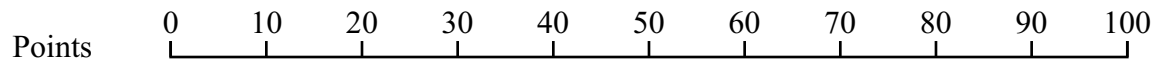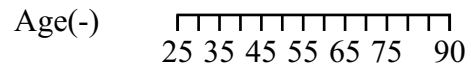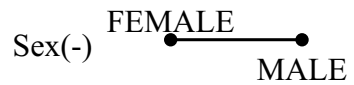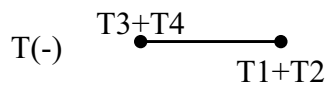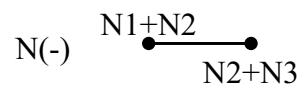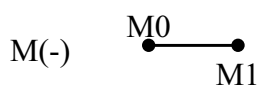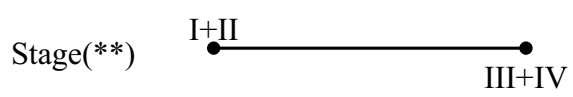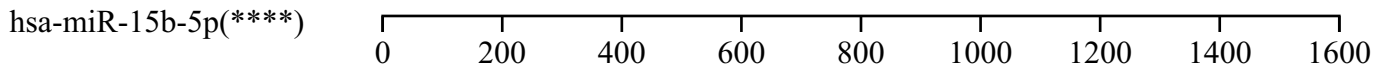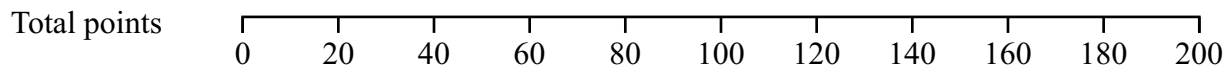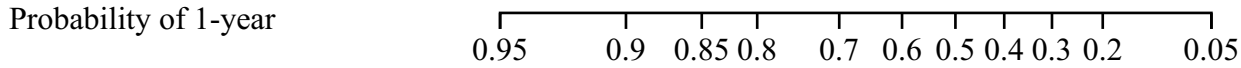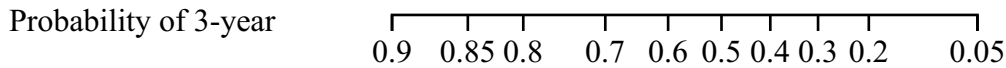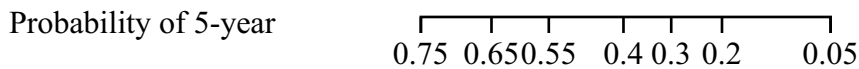

Supplement: Supplemental Information 1 — All the raw data, result images and running codes in this paper, including qRT-PCR data and cell behavior measurements. [file peerj-14-20538-s001.zip › Supplementary files 1/result 5/miR-15b-5p-nomogram.pdf]

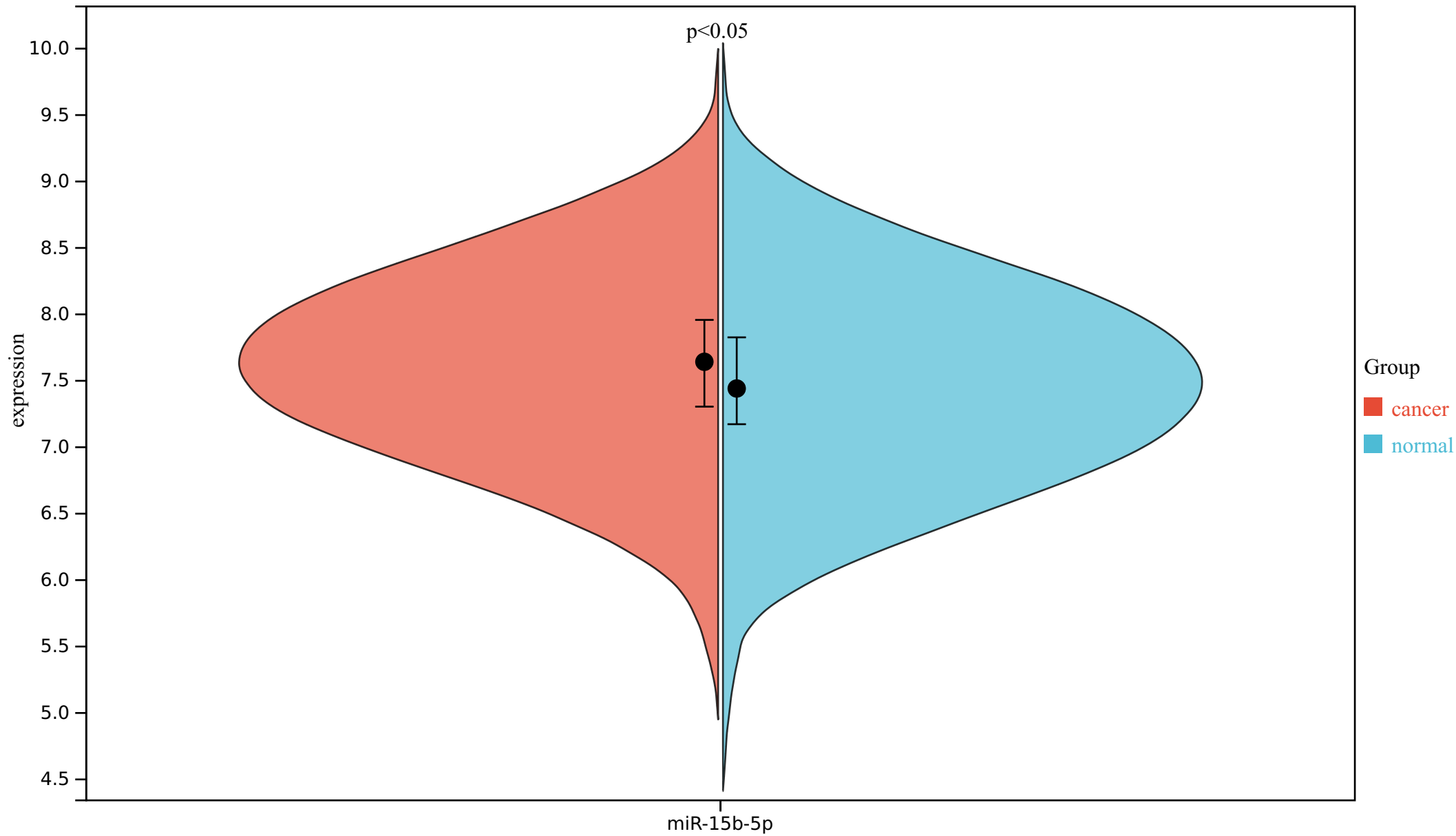

Supplement: Supplemental Information 1 — All the raw data, result images and running codes in this paper, including qRT-PCR data and cell behavior measurements. [file peerj-14-20538-s001.zip › Supplementary files 1/result 5/miRNA expression validation.pdf]

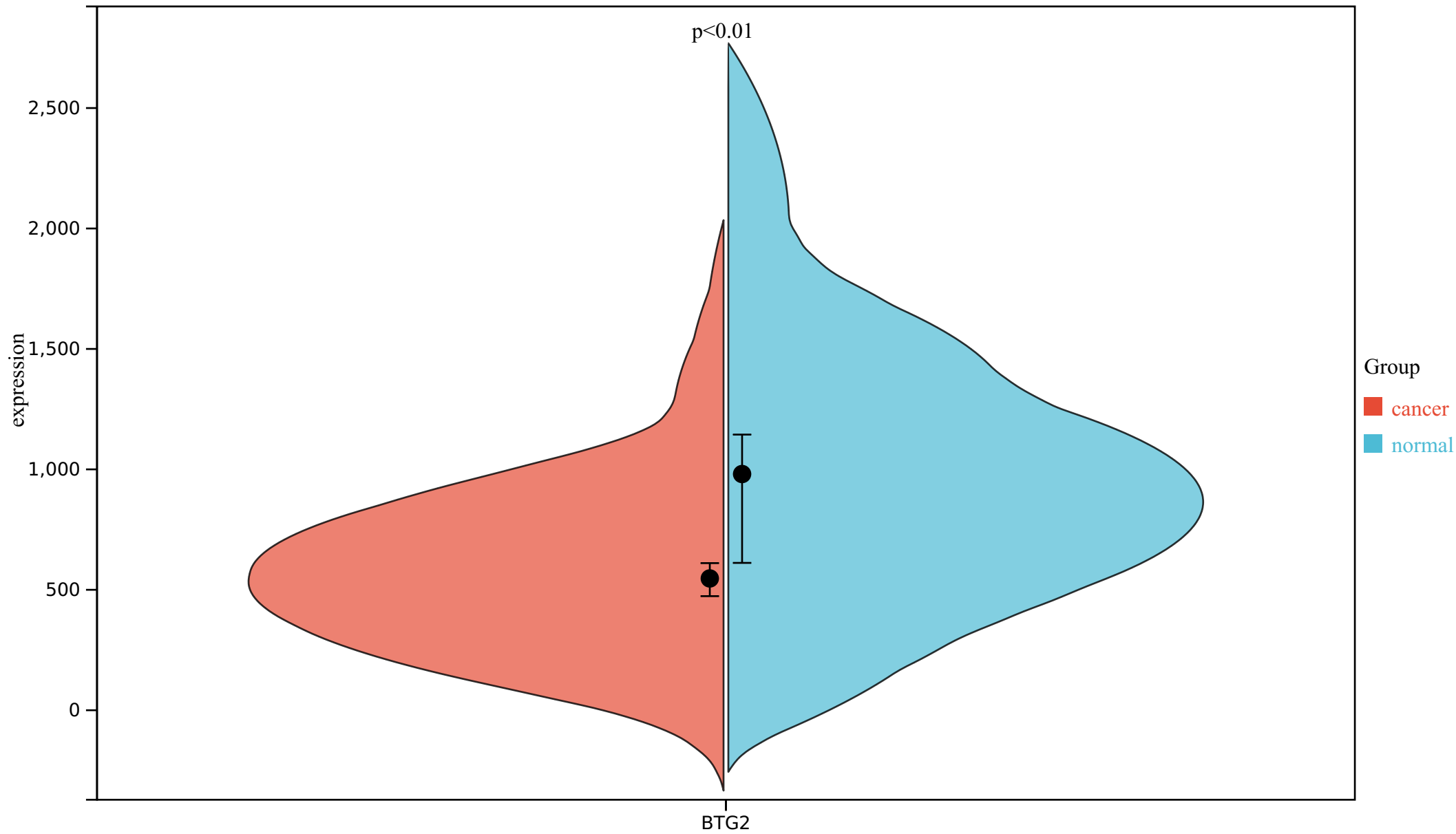

Supplement: Supplemental Information 1 — All the raw data, result images and running codes in this paper, including qRT-PCR data and cell behavior measurements. [file peerj-14-20538-s001.zip › Supplementary files 1/result 5/mRNA expression validation.pdf]

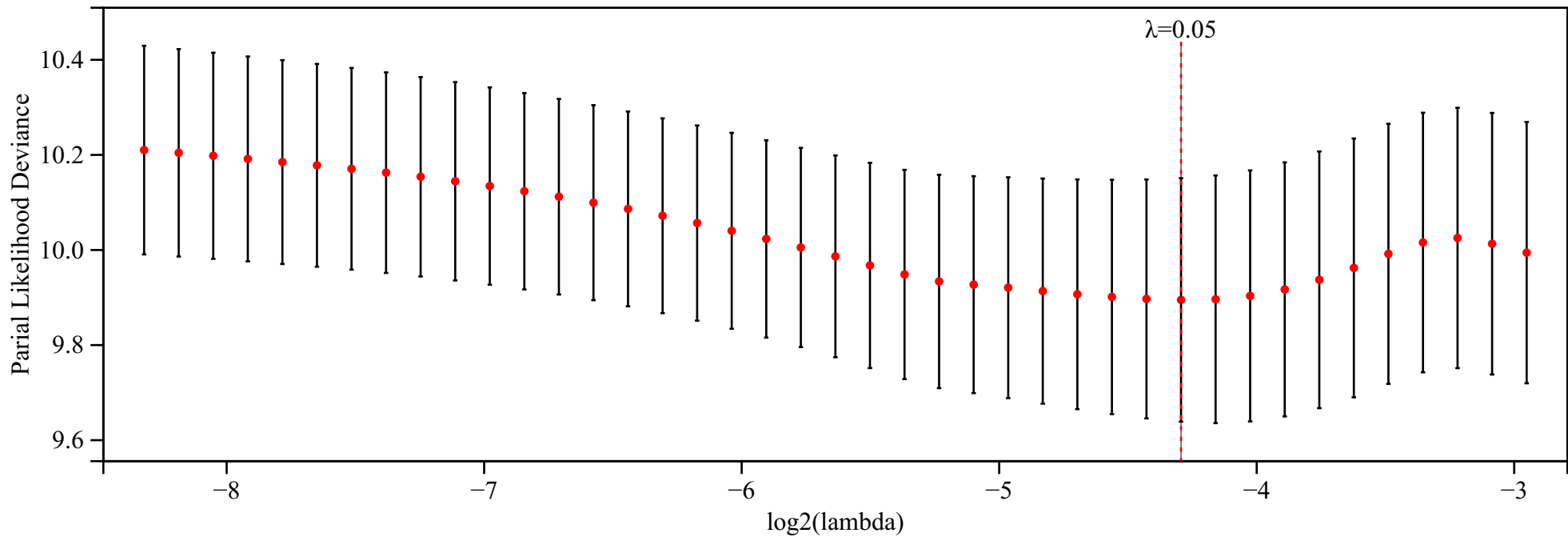

Supplement: Supplemental Information 1 — All the raw data, result images and running codes in this paper, including qRT-PCR data and cell behavior measurements. [file peerj-14-20538-s001.zip › Supplementary files 1/result 5/mRNA-LASSO-10 fold.pdf]

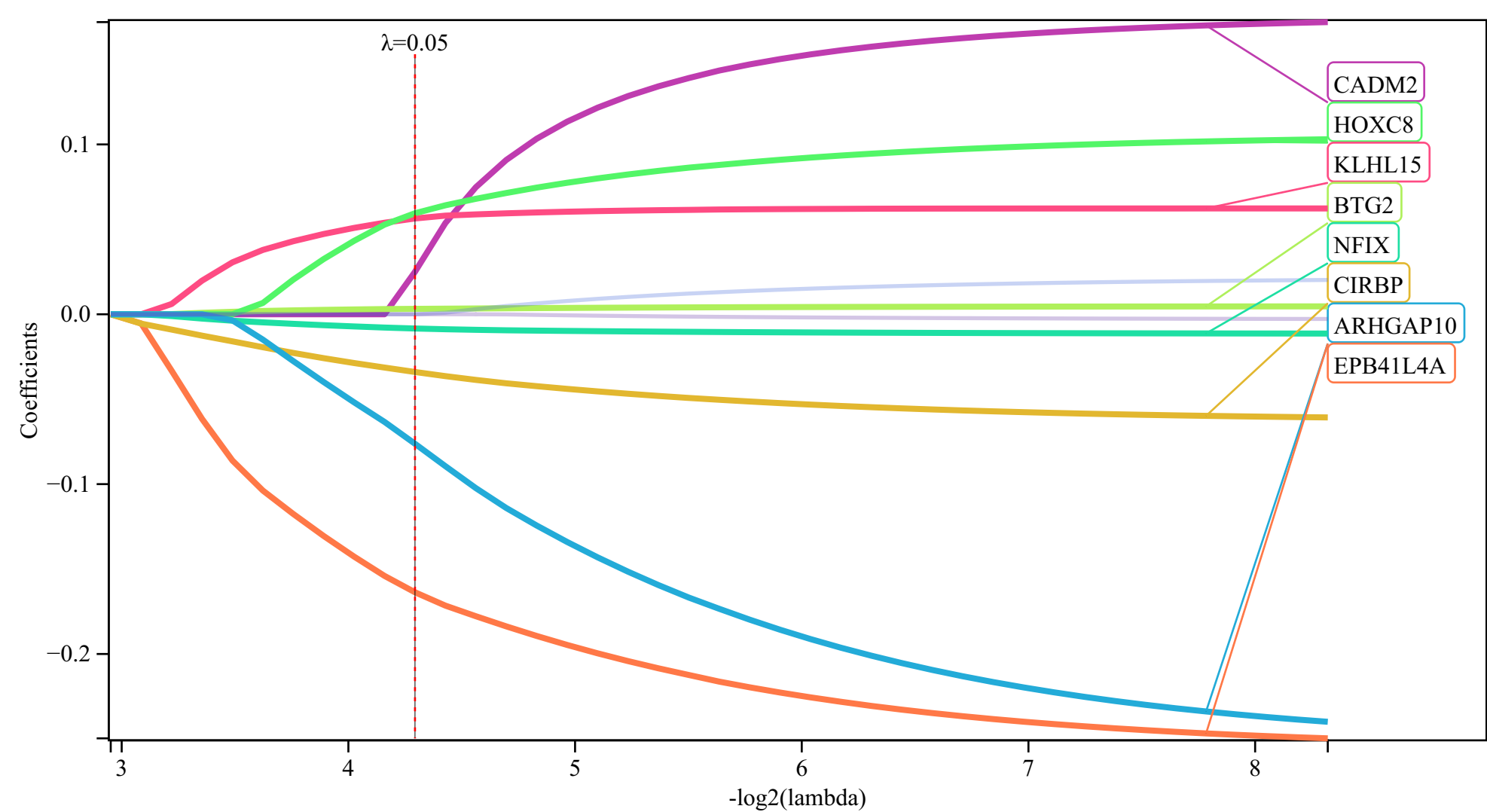

Supplement: Supplemental Information 1 — All the raw data, result images and running codes in this paper, including qRT-PCR data and cell behavior measurements. [file peerj-14-20538-s001.zip › Supplementary files 1/result 5/mRNA_lasso-cox.pdf]

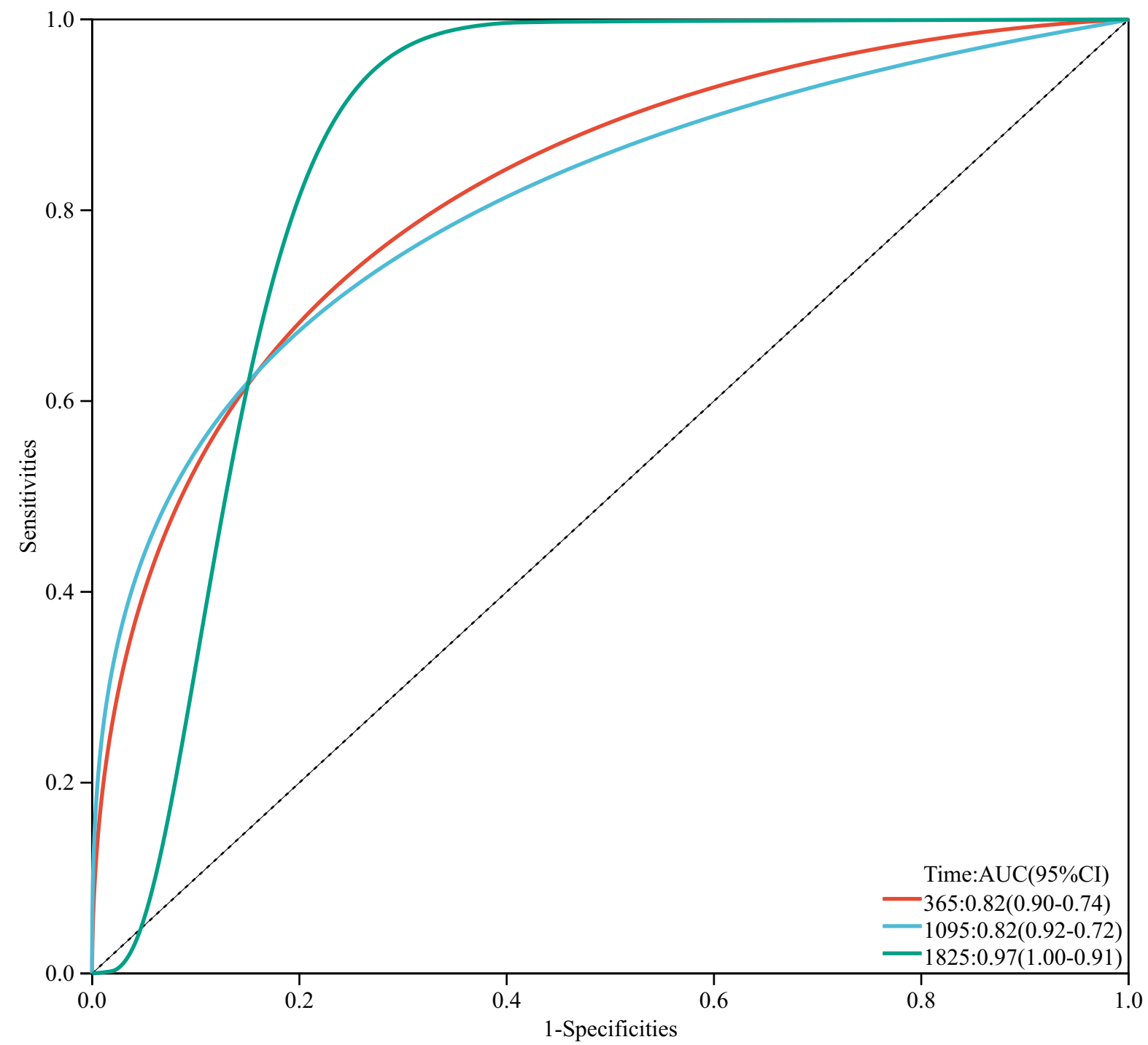

Supplement: Supplemental Information 1 — All the raw data, result images and running codes in this paper, including qRT-PCR data and cell behavior measurements. [file peerj-14-20538-s001.zip › Supplementary files 1/result 5/ROC-miRNA.pdf]

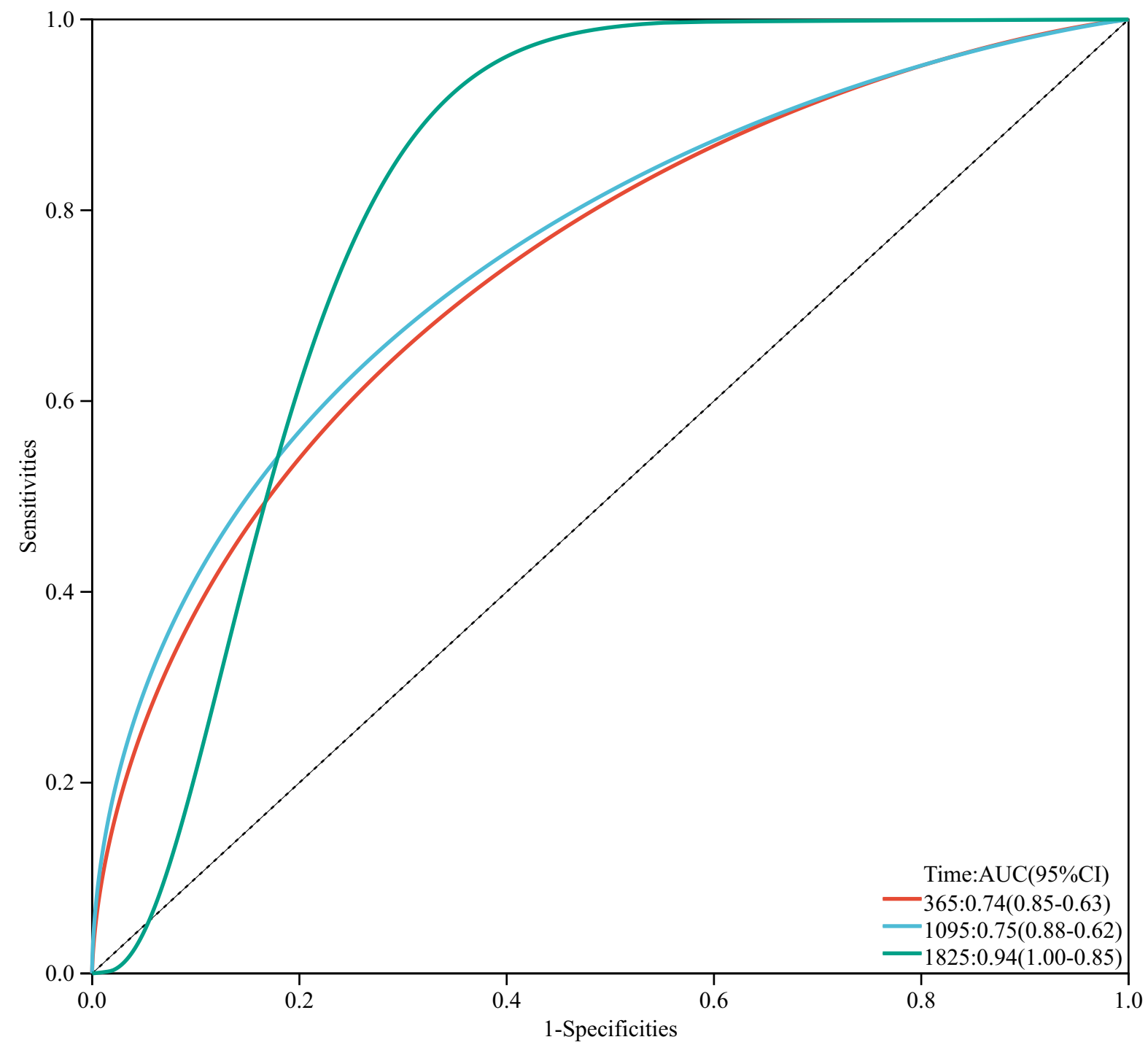

Supplement: Supplemental Information 1 — All the raw data, result images and running codes in this paper, including qRT-PCR data and cell behavior measurements. [file peerj-14-20538-s001.zip › Supplementary files 1/result 5/ROC-mRNA.pdf]

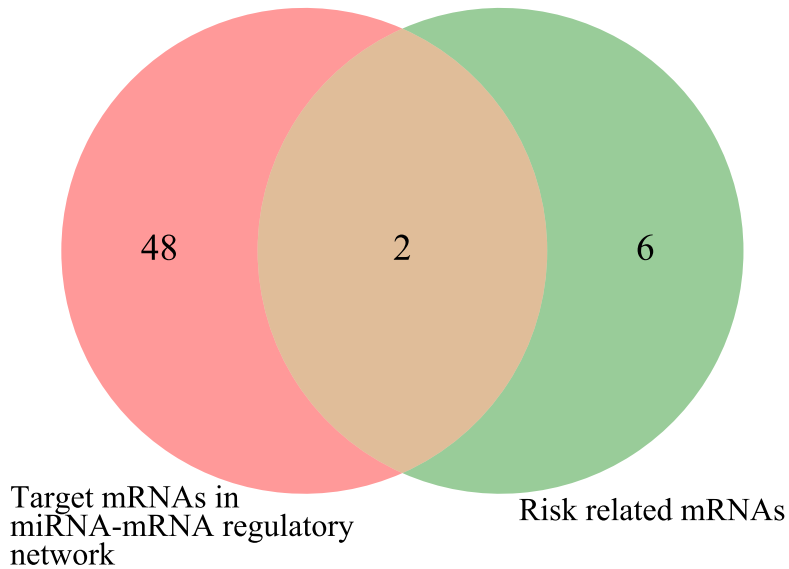

Supplement: Supplemental Information 1 — All the raw data, result images and running codes in this paper, including qRT-PCR data and cell behavior measurements. [file peerj-14-20538-s001.zip › Supplementary files 1/result 5/wenen.pdf]

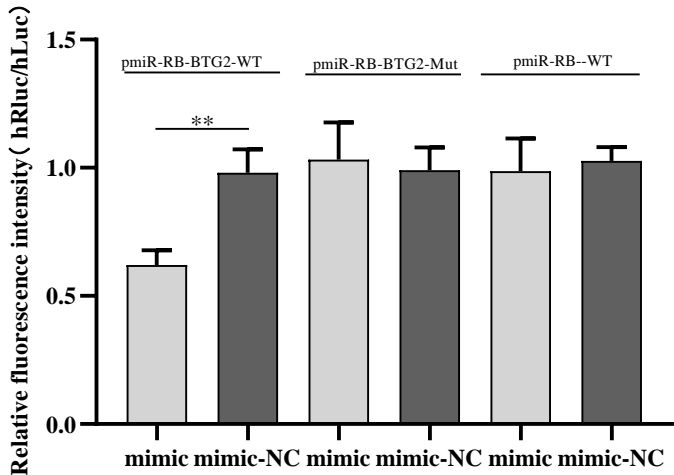

Supplement: Supplemental Information 1 — All the raw data, result images and running codes in this paper, including qRT-PCR data and cell behavior measurements. [file peerj-14-20538-s001.zip › Supplementary files 1/result 6/Dual-Luciferase.pdf]

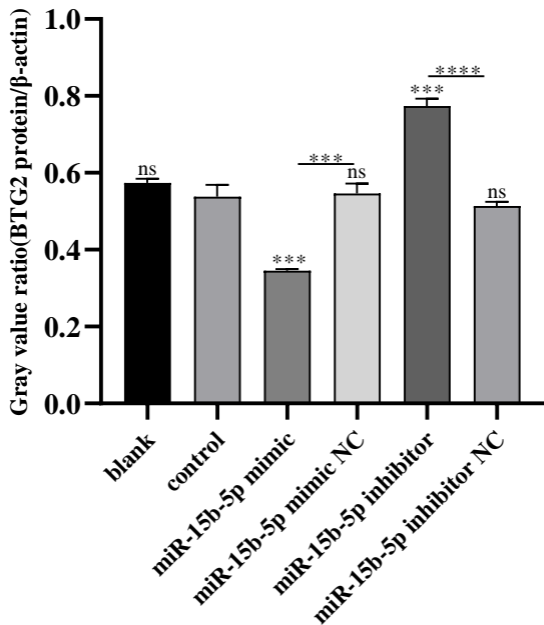

Supplement: Supplemental Information 1 — All the raw data, result images and running codes in this paper, including qRT-PCR data and cell behavior measurements. [file peerj-14-20538-s001.zip › Supplementary files 1/result 6/Gray.pdf]

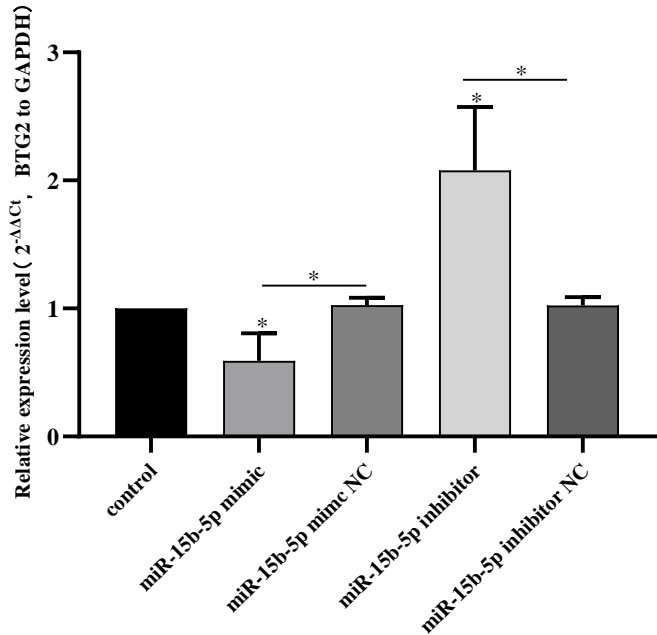

Supplement: Supplemental Information 1 — All the raw data, result images and running codes in this paper, including qRT-PCR data and cell behavior measurements. [file peerj-14-20538-s001.zip › Supplementary files 1/result 6/miR-15b-5p to BTG2 effction/miRNA-mRNA effection.pdf]

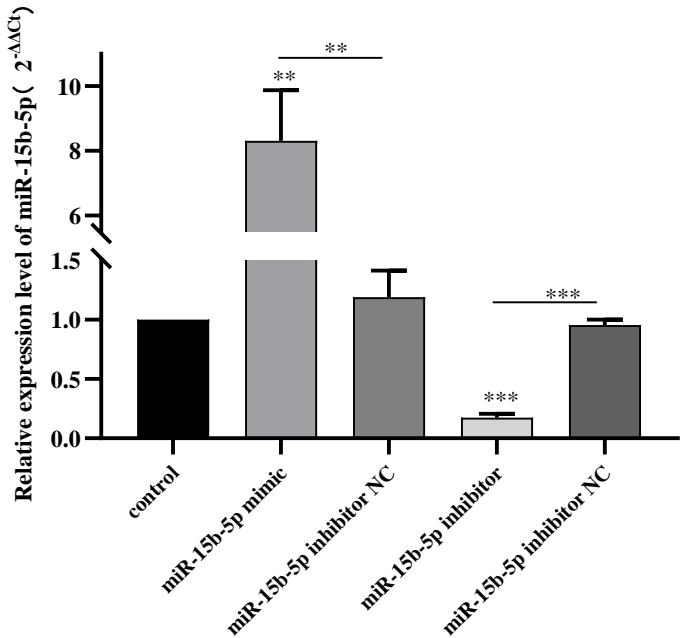

Supplement: Supplemental Information 1 — All the raw data, result images and running codes in this paper, including qRT-PCR data and cell behavior measurements. [file peerj-14-20538-s001.zip › Supplementary files 1/result 6/miRNA traslation effction.pdf]

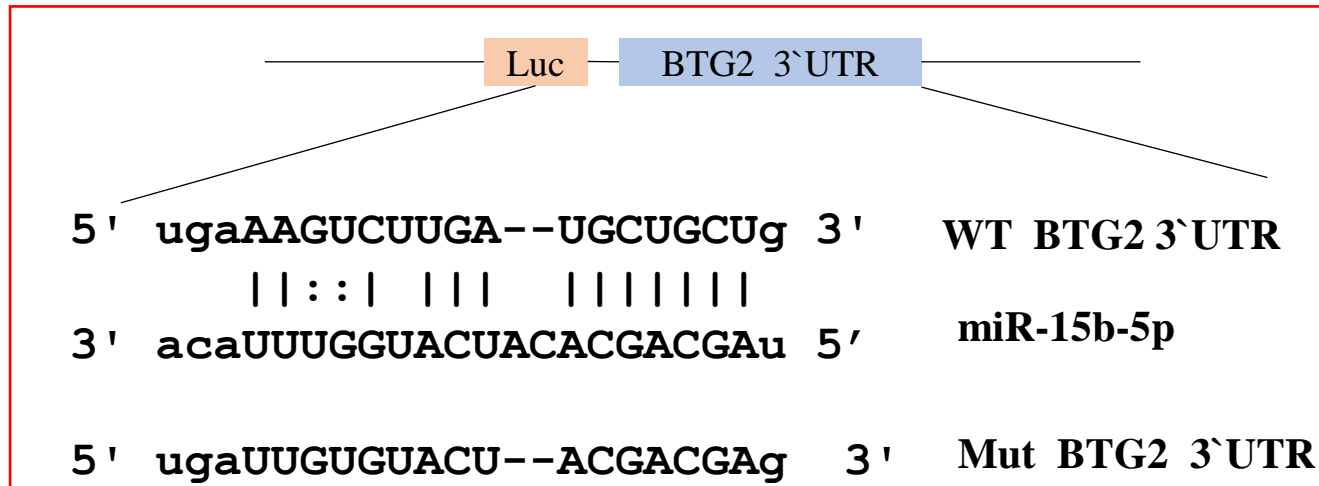

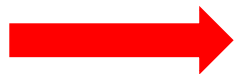

Supplement: Supplemental Information 1 — All the raw data, result images and running codes in this paper, including qRT-PCR data and cell behavior measurements. [file peerj-14-20538-s001.zip › Supplementary files 1/result 6/wound9.pdf]

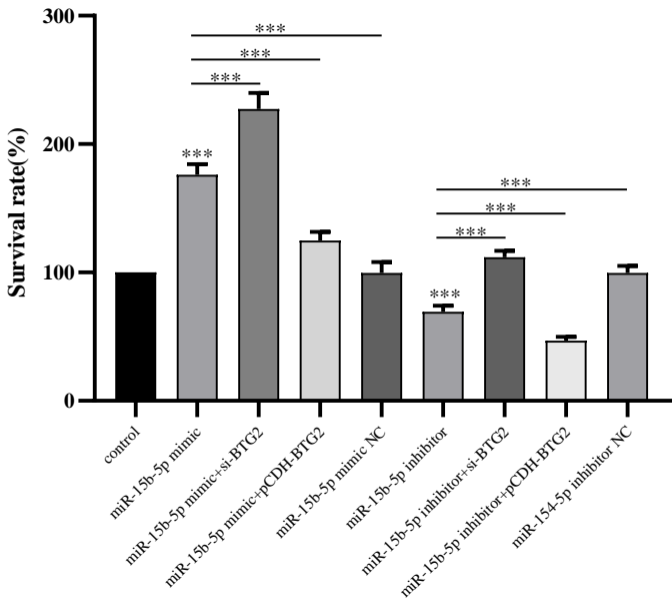

Supplement: Supplemental Information 1 — All the raw data, result images and running codes in this paper, including qRT-PCR data and cell behavior measurements. [file peerj-14-20538-s001.zip › Supplementary files 1/result 7/CCK8.pdf]

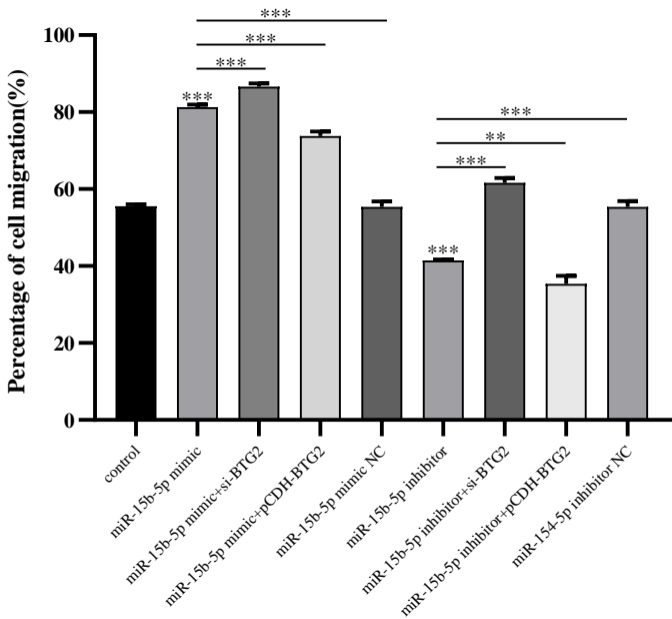

Supplement: Supplemental Information 1 — All the raw data, result images and running codes in this paper, including qRT-PCR data and cell behavior measurements. [file peerj-14-20538-s001.zip › Supplementary files 1/result 7/qianyi.pdf]

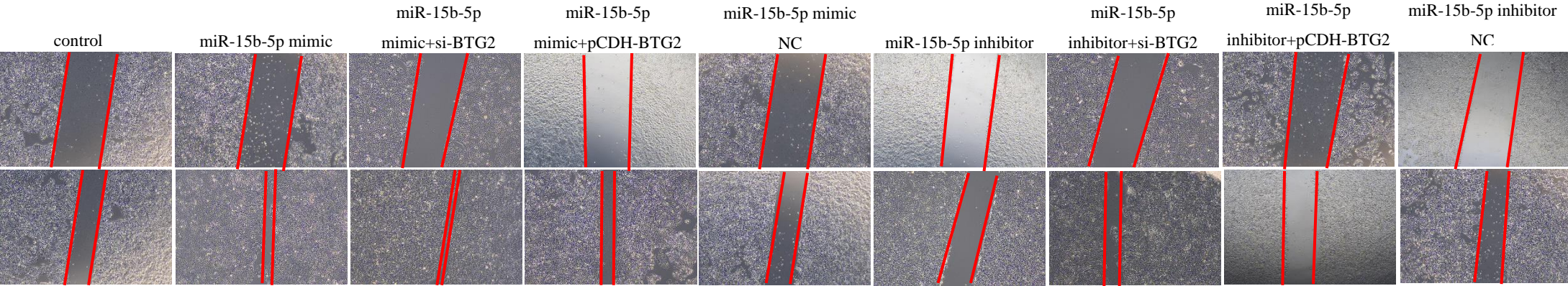

Supplement: Supplemental Information 1 — All the raw data, result images and running codes in this paper, including qRT-PCR data and cell behavior measurements. [file peerj-14-20538-s001.zip › Supplementary files 1/result 7/qianyi2.pdf]

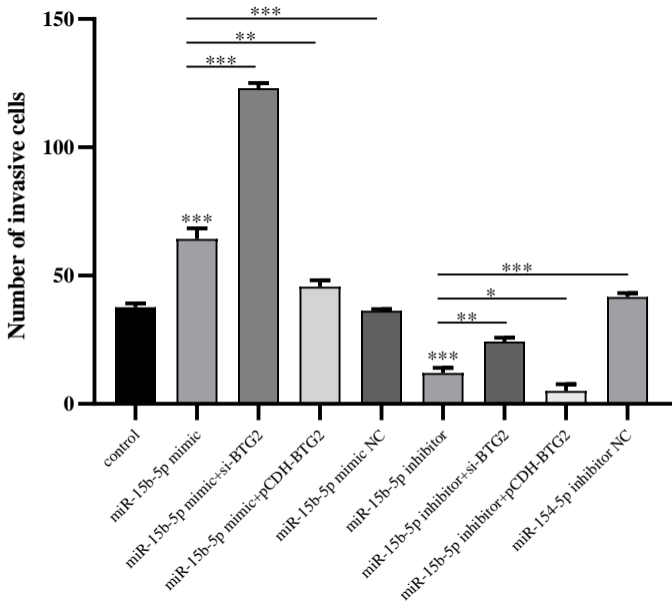

Supplement: Supplemental Information 1 — All the raw data, result images and running codes in this paper, including qRT-PCR data and cell behavior measurements. [file peerj-14-20538-s001.zip › Supplementary files 1/result 7/qinxi.pdf]

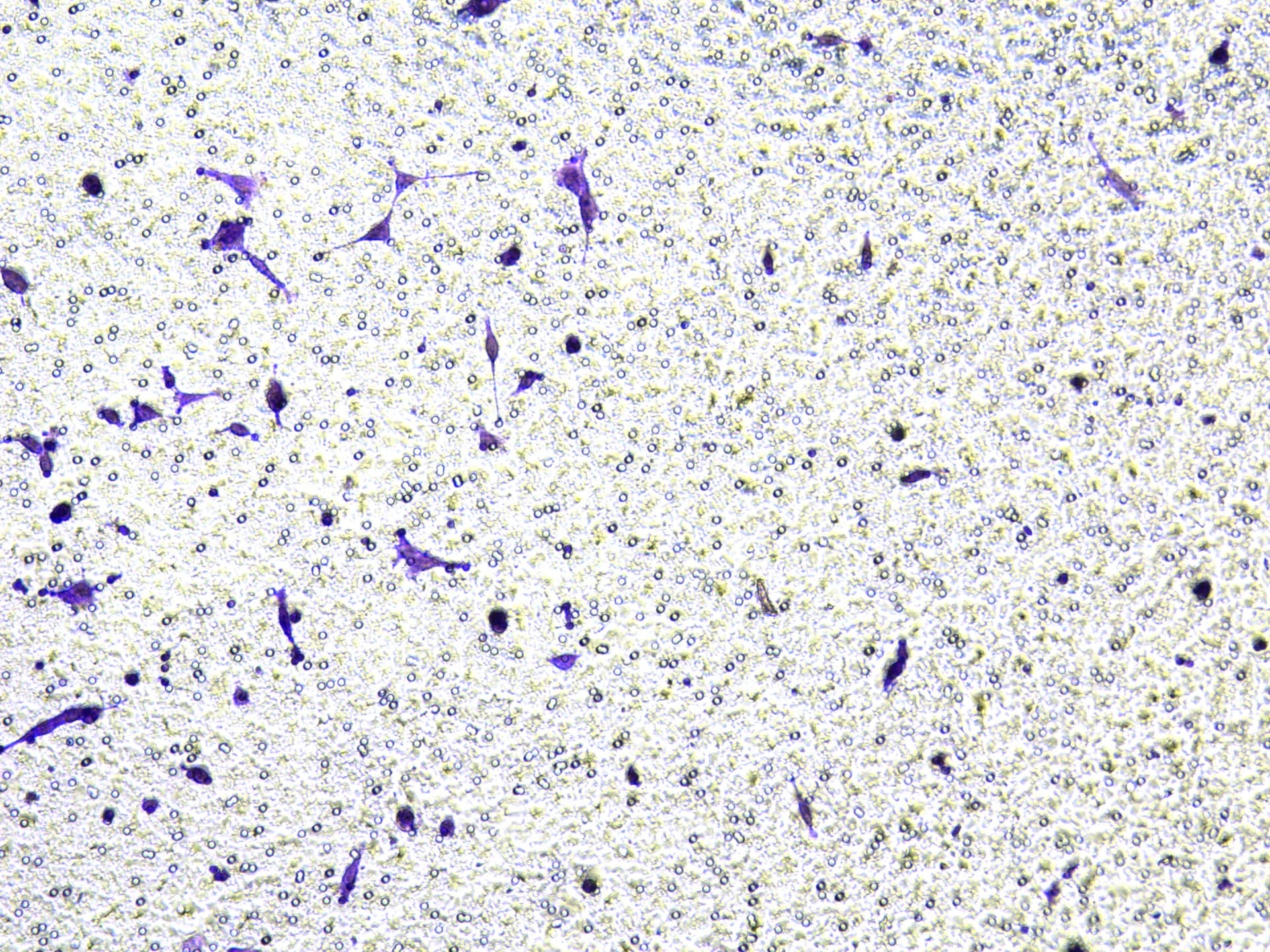

Supplement: Supplemental Information 1 — All the raw data, result images and running codes in this paper, including qRT-PCR data and cell behavior measurements. [file peerj-14-20538-s001.zip › Supplementary files 1/result 7/transwell/control .jpg]

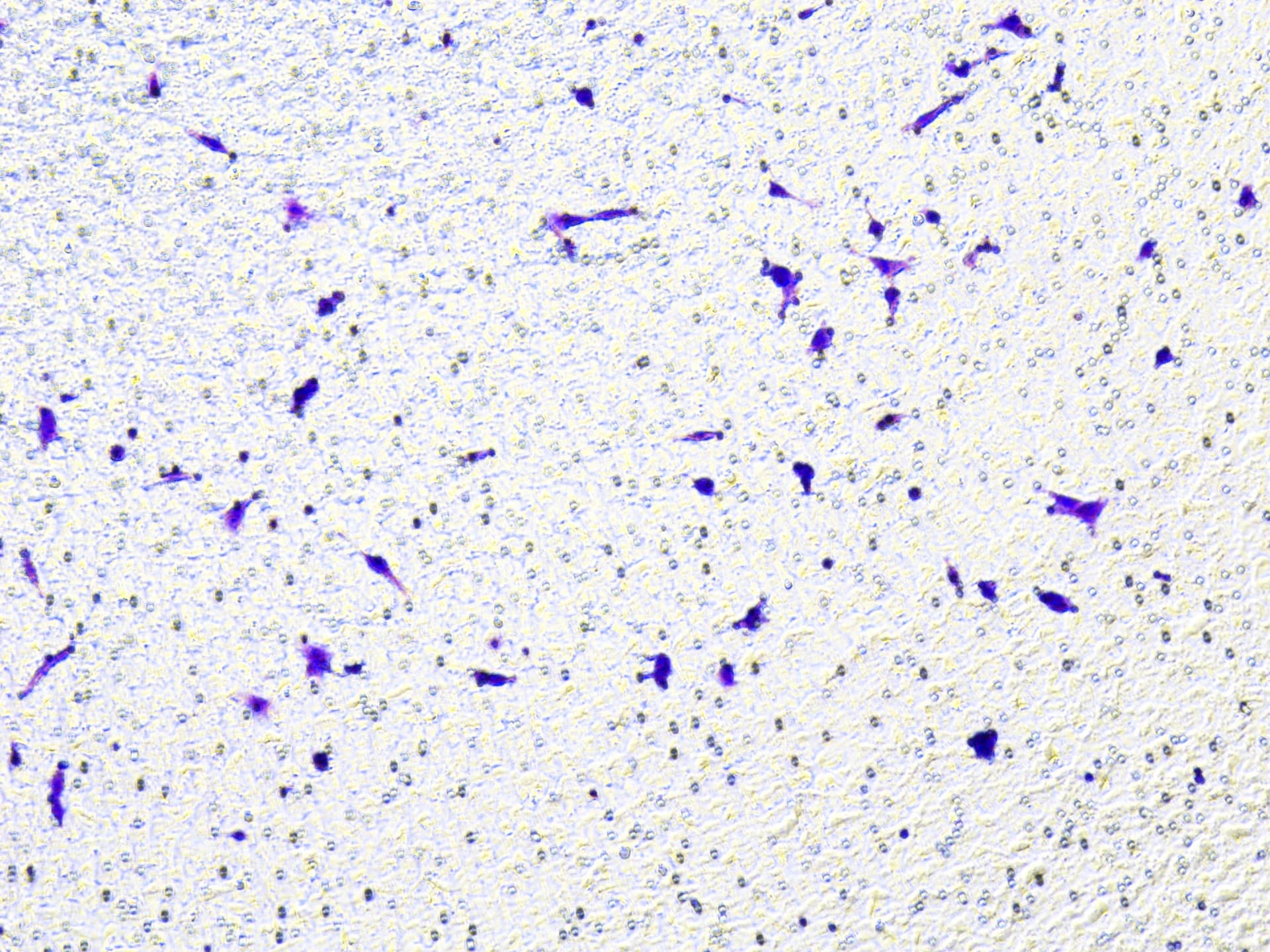

Supplement: Supplemental Information 1 — All the raw data, result images and running codes in this paper, including qRT-PCR data and cell behavior measurements. [file peerj-14-20538-s001.zip › Supplementary files 1/result 7/transwell/inhibitor NC.jpg]

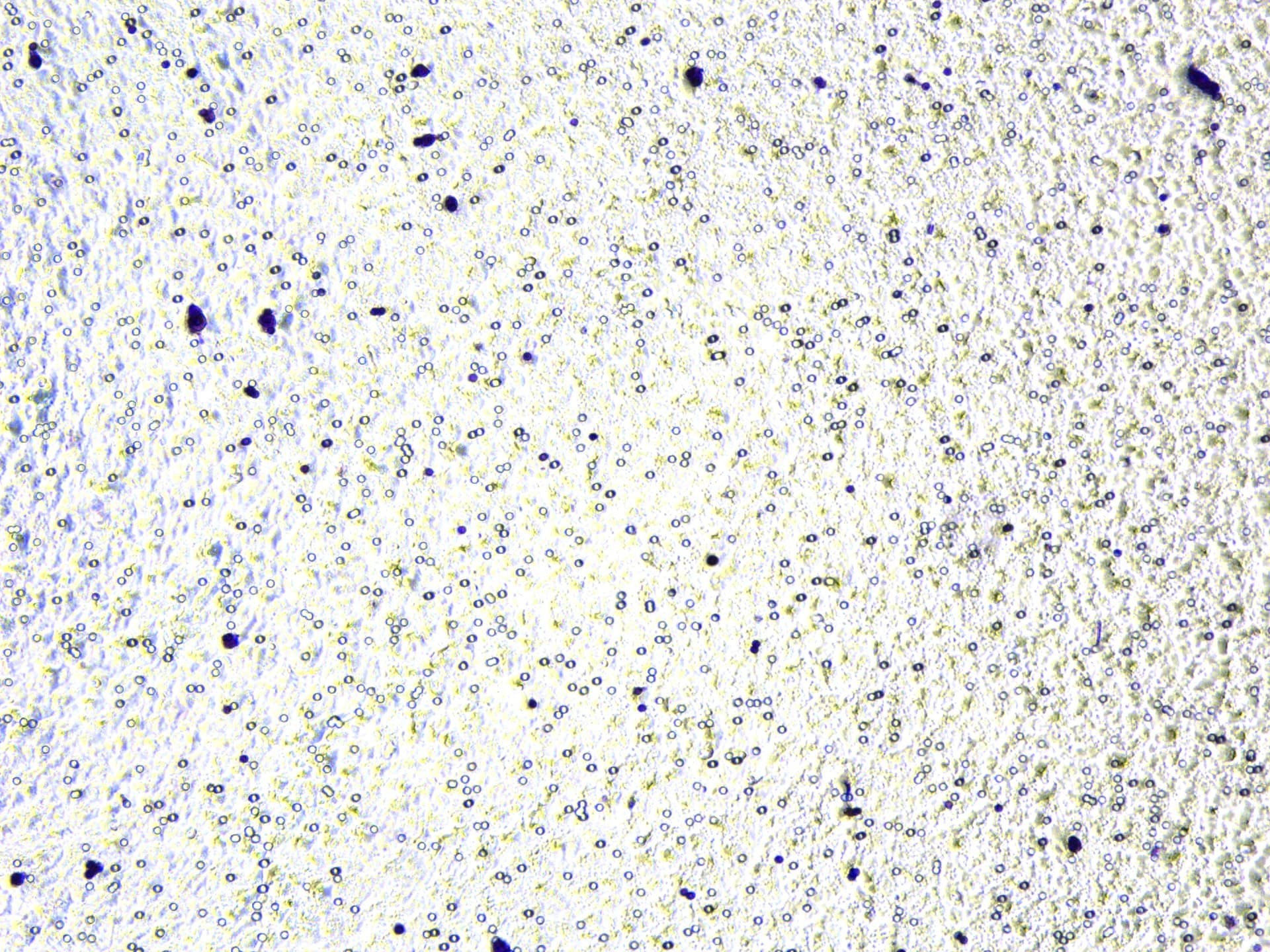

Supplement: Supplemental Information 1 — All the raw data, result images and running codes in this paper, including qRT-PCR data and cell behavior measurements. [file peerj-14-20538-s001.zip › Supplementary files 1/result 7/transwell/inhibitor+pCDH.jpg]

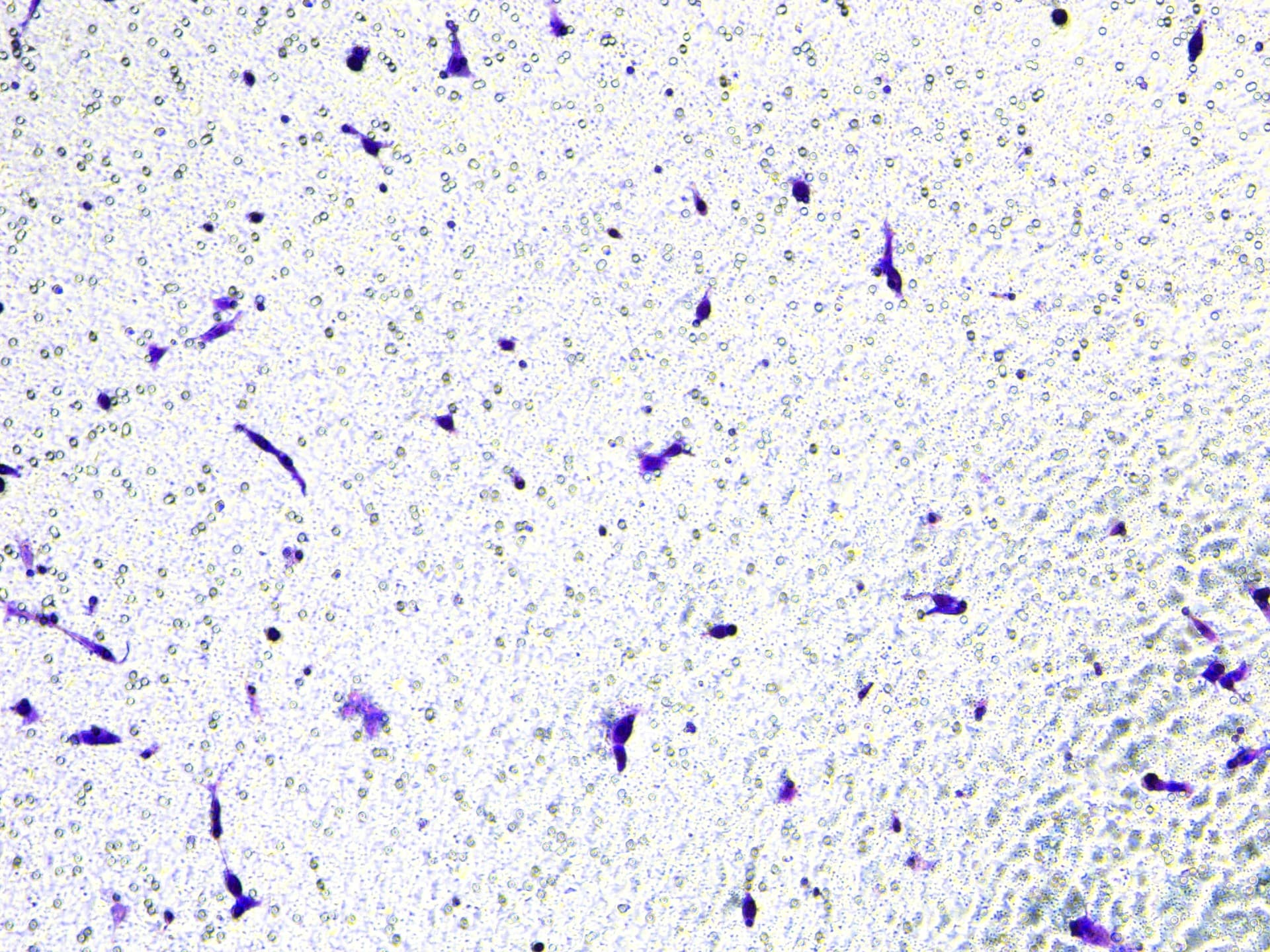

Supplement: Supplemental Information 1 — All the raw data, result images and running codes in this paper, including qRT-PCR data and cell behavior measurements. [file peerj-14-20538-s001.zip › Supplementary files 1/result 7/transwell/inhibitor+si.jpg]

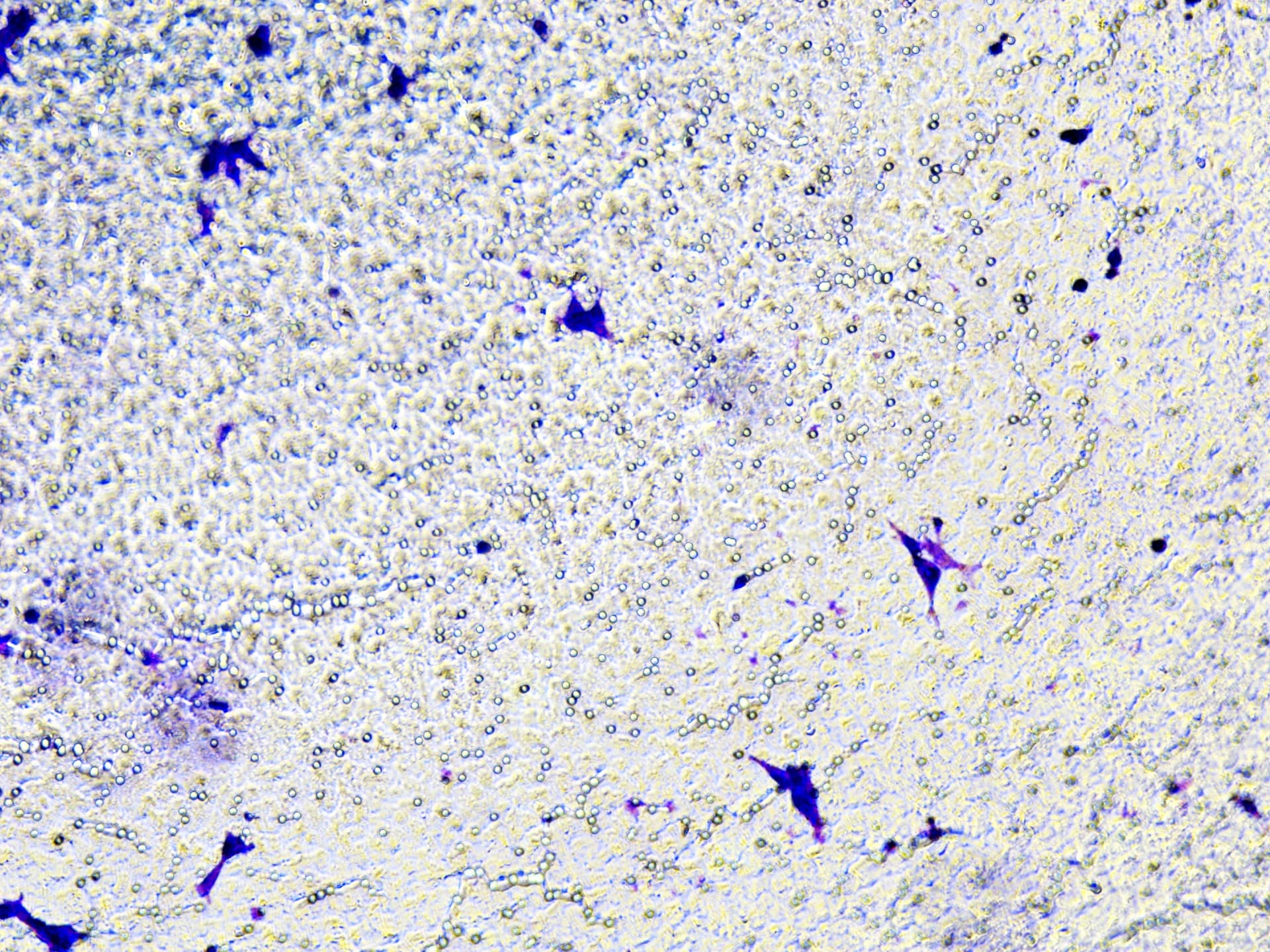

Supplement: Supplemental Information 1 — All the raw data, result images and running codes in this paper, including qRT-PCR data and cell behavior measurements. [file peerj-14-20538-s001.zip › Supplementary files 1/result 7/transwell/inhibitor.jpg]

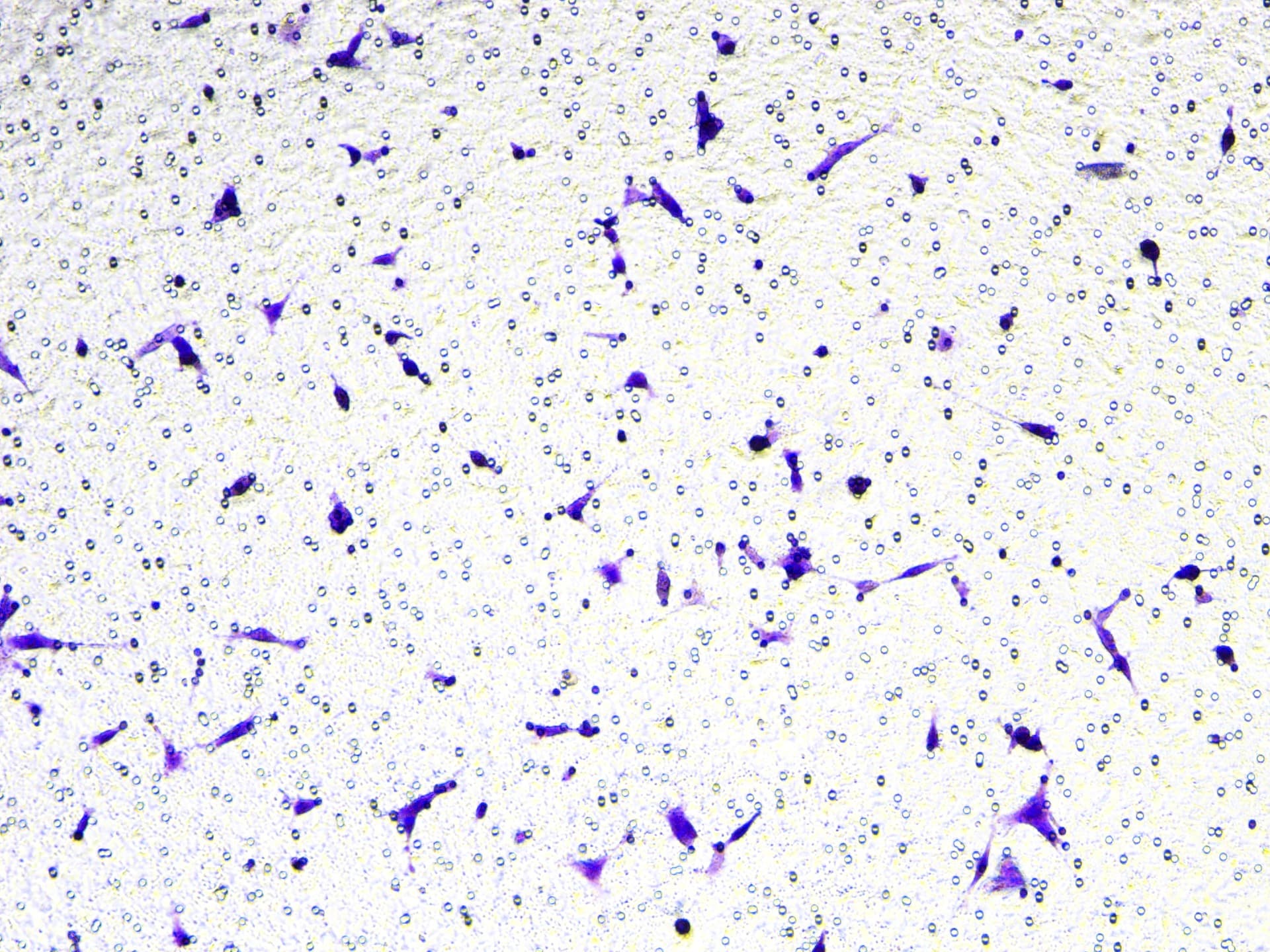

Supplement: Supplemental Information 1 — All the raw data, result images and running codes in this paper, including qRT-PCR data and cell behavior measurements. [file peerj-14-20538-s001.zip › Supplementary files 1/result 7/transwell/mimic NC.jpg]

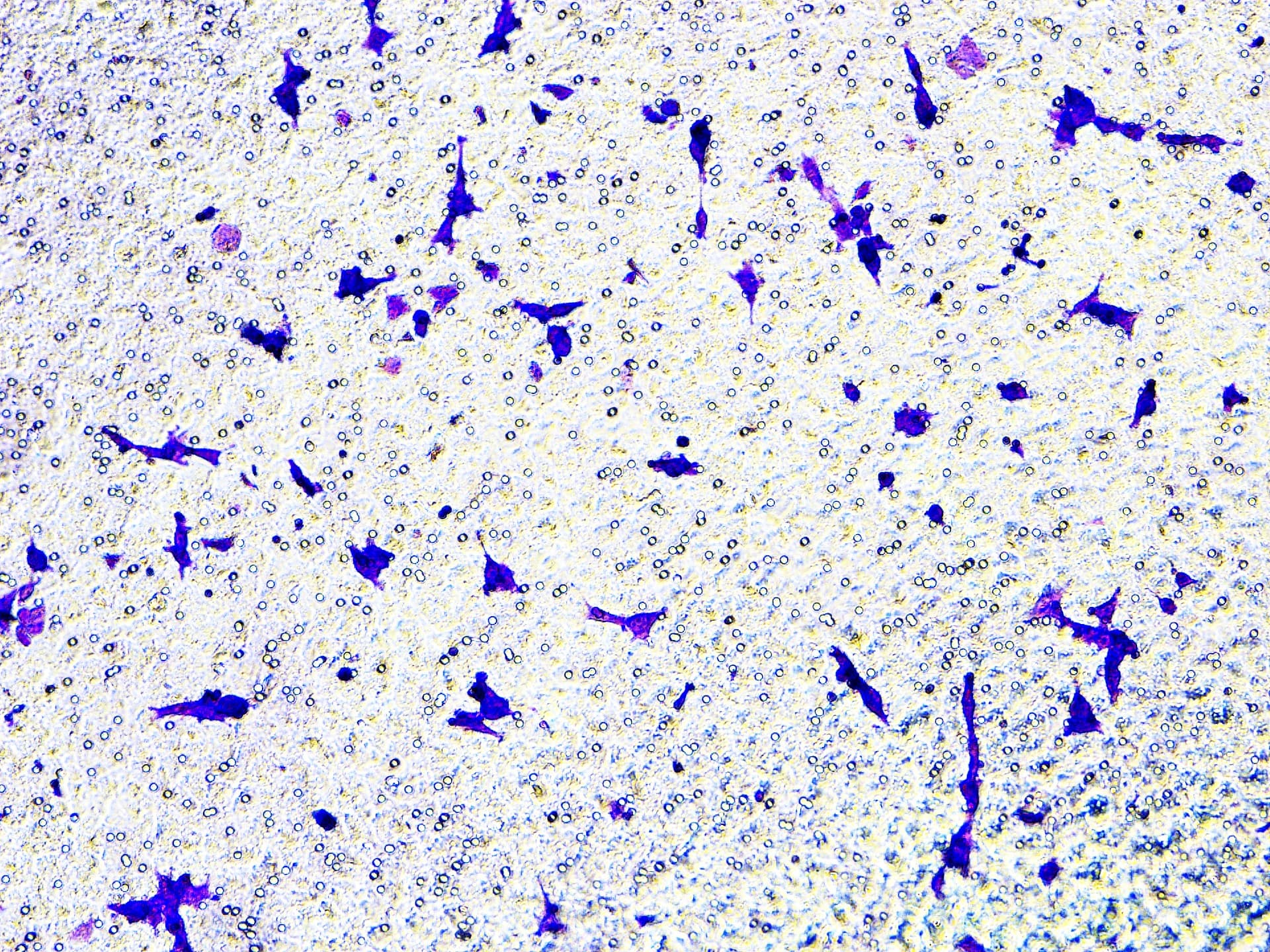

Supplement: Supplemental Information 1 — All the raw data, result images and running codes in this paper, including qRT-PCR data and cell behavior measurements. [file peerj-14-20538-s001.zip › Supplementary files 1/result 7/transwell/mimic+pCDH.jpg]

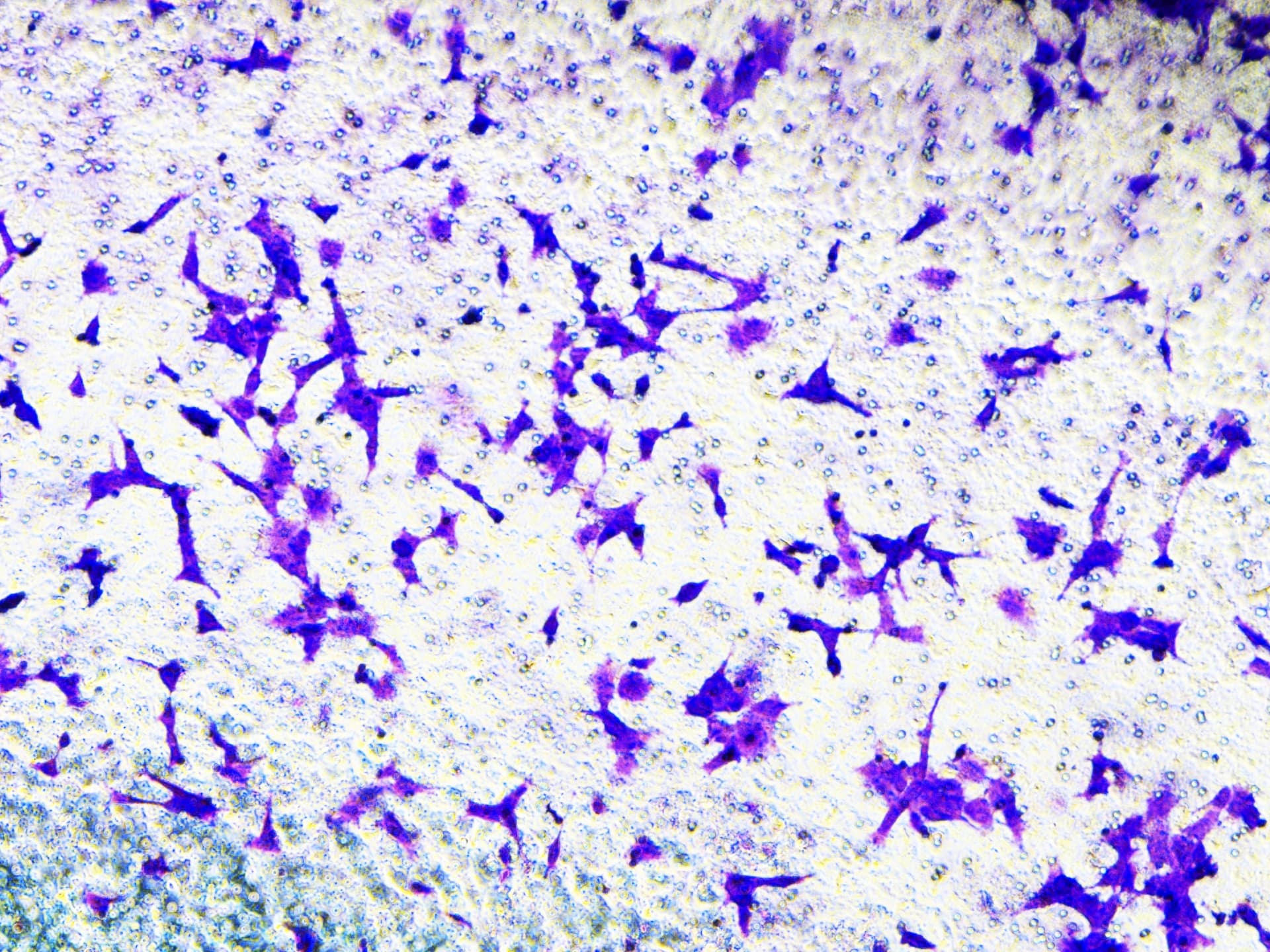

Supplement: Supplemental Information 1 — All the raw data, result images and running codes in this paper, including qRT-PCR data and cell behavior measurements. [file peerj-14-20538-s001.zip › Supplementary files 1/result 7/transwell/mimic+si .jpg]

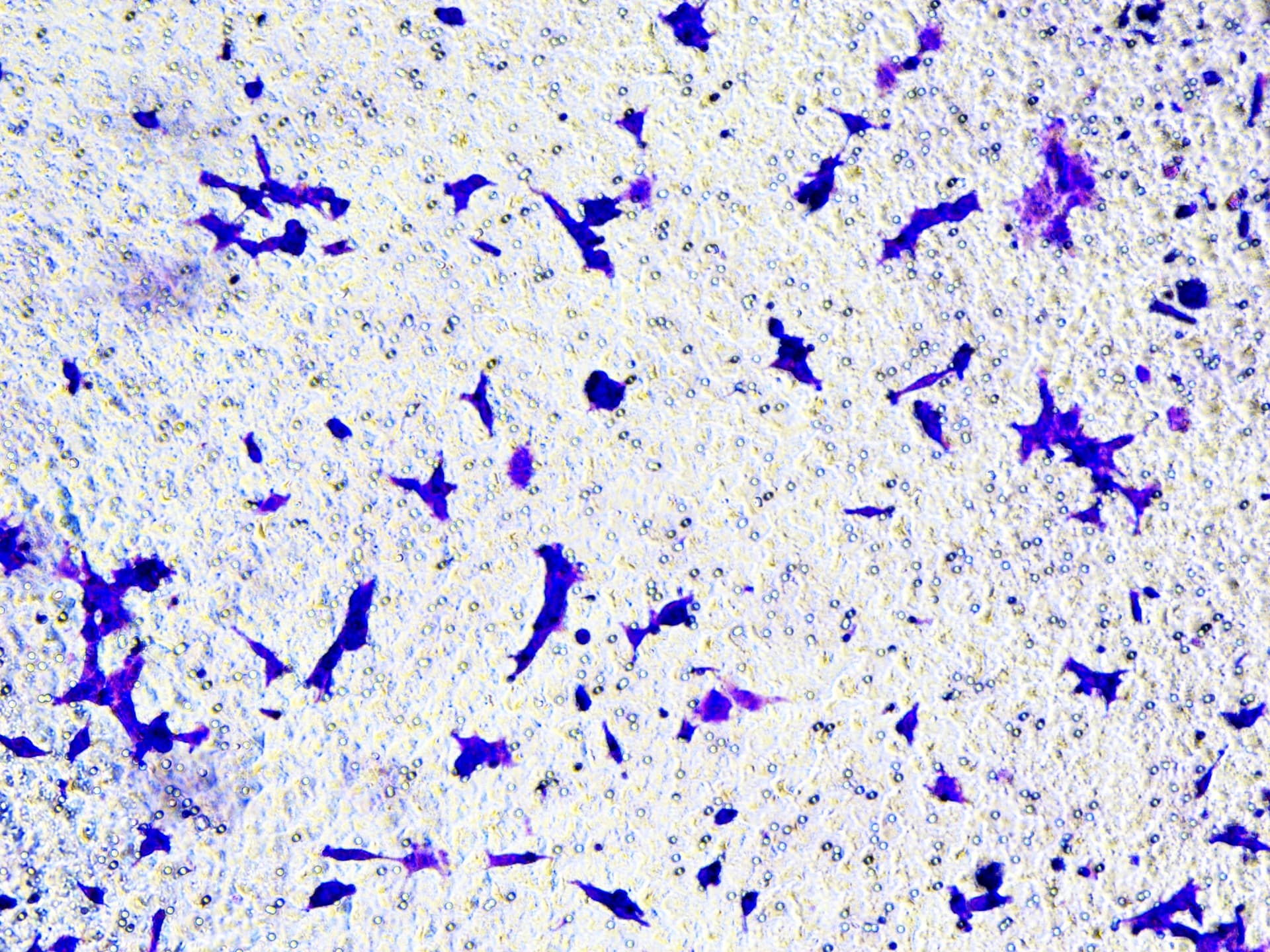

Supplement: Supplemental Information 1 — All the raw data, result images and running codes in this paper, including qRT-PCR data and cell behavior measurements. [file peerj-14-20538-s001.zip › Supplementary files 1/result 7/transwell/mimic.jpg]

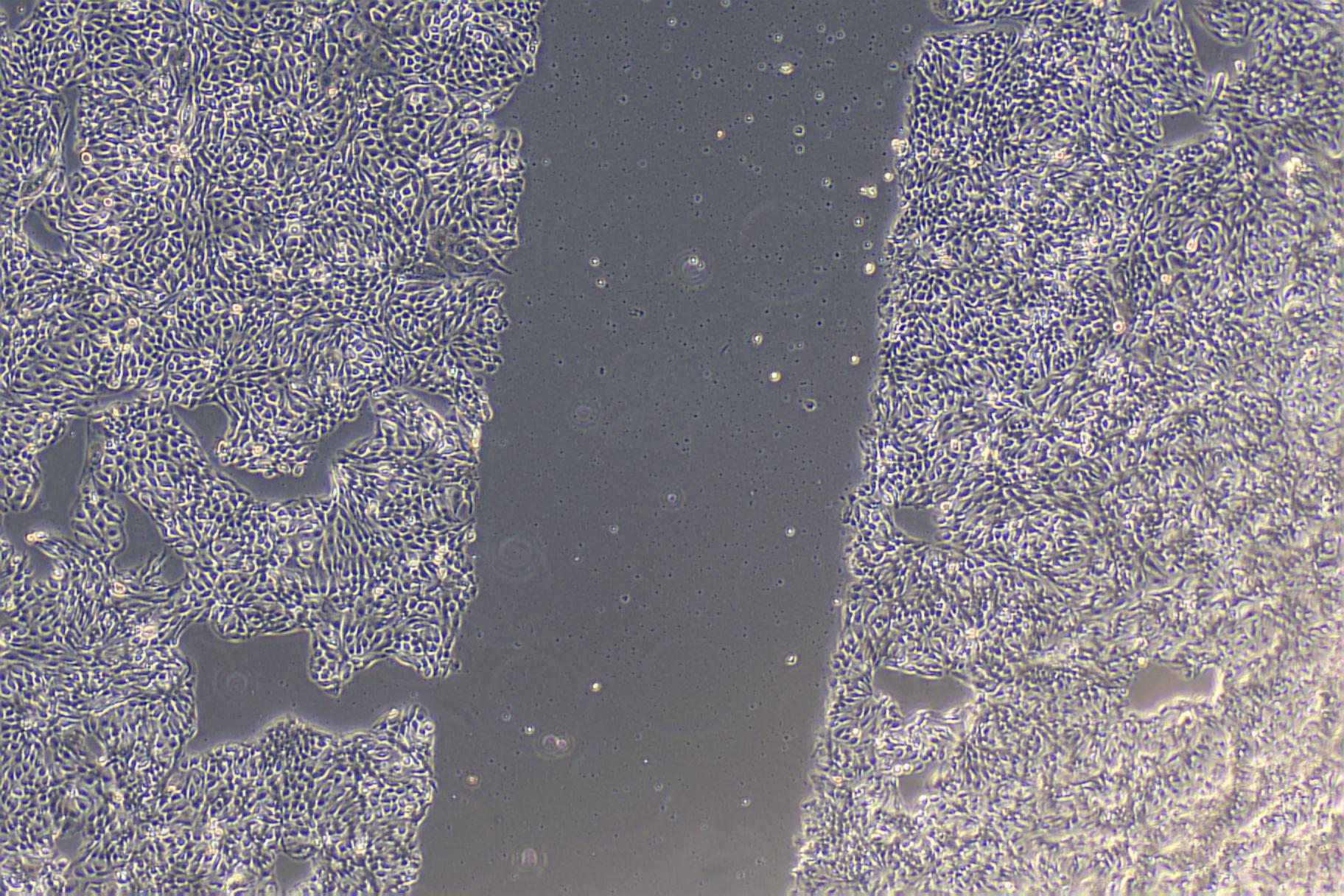

Supplement: Supplemental Information 1 — All the raw data, result images and running codes in this paper, including qRT-PCR data and cell behavior measurements. [file peerj-14-20538-s001.zip › Supplementary files 1/result 7/wound-healing/0h control.jpg]

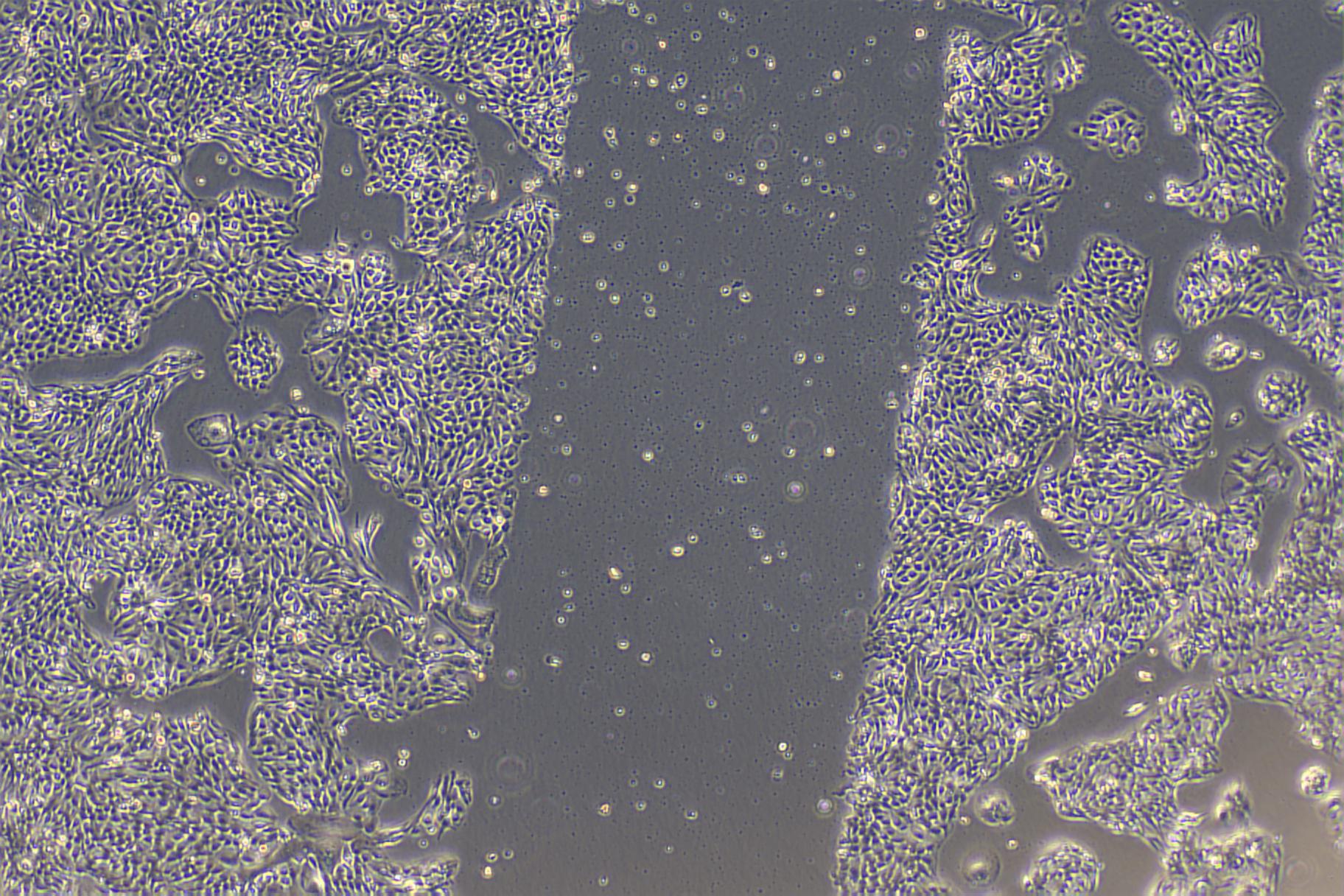

Supplement: Supplemental Information 1 — All the raw data, result images and running codes in this paper, including qRT-PCR data and cell behavior measurements. [file peerj-14-20538-s001.zip › Supplementary files 1/result 7/wound-healing/0h inhibitor NC.jpg]

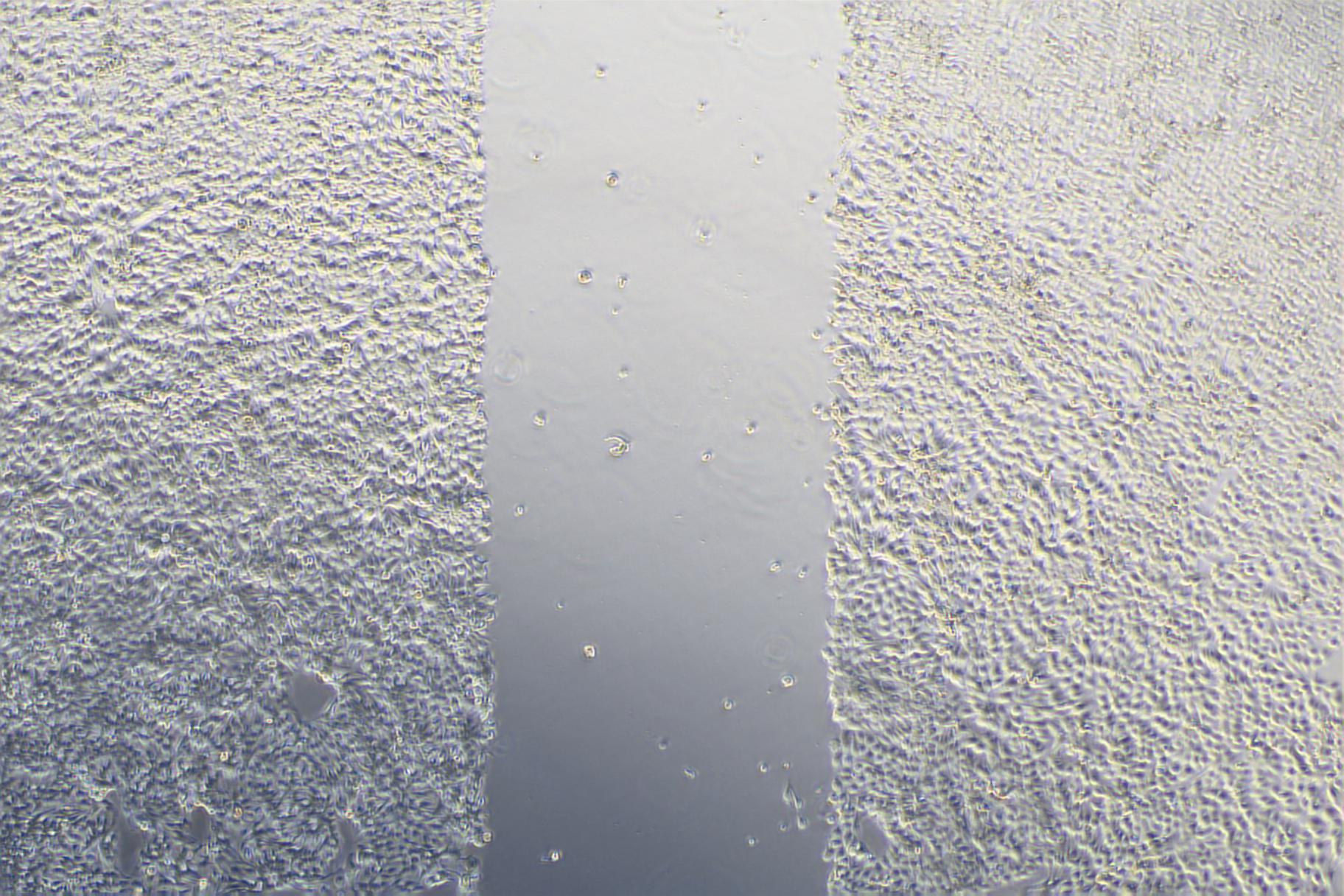

Supplement: Supplemental Information 1 — All the raw data, result images and running codes in this paper, including qRT-PCR data and cell behavior measurements. [file peerj-14-20538-s001.zip › Supplementary files 1/result 7/wound-healing/0h inhibitor+pCDH.jpg]

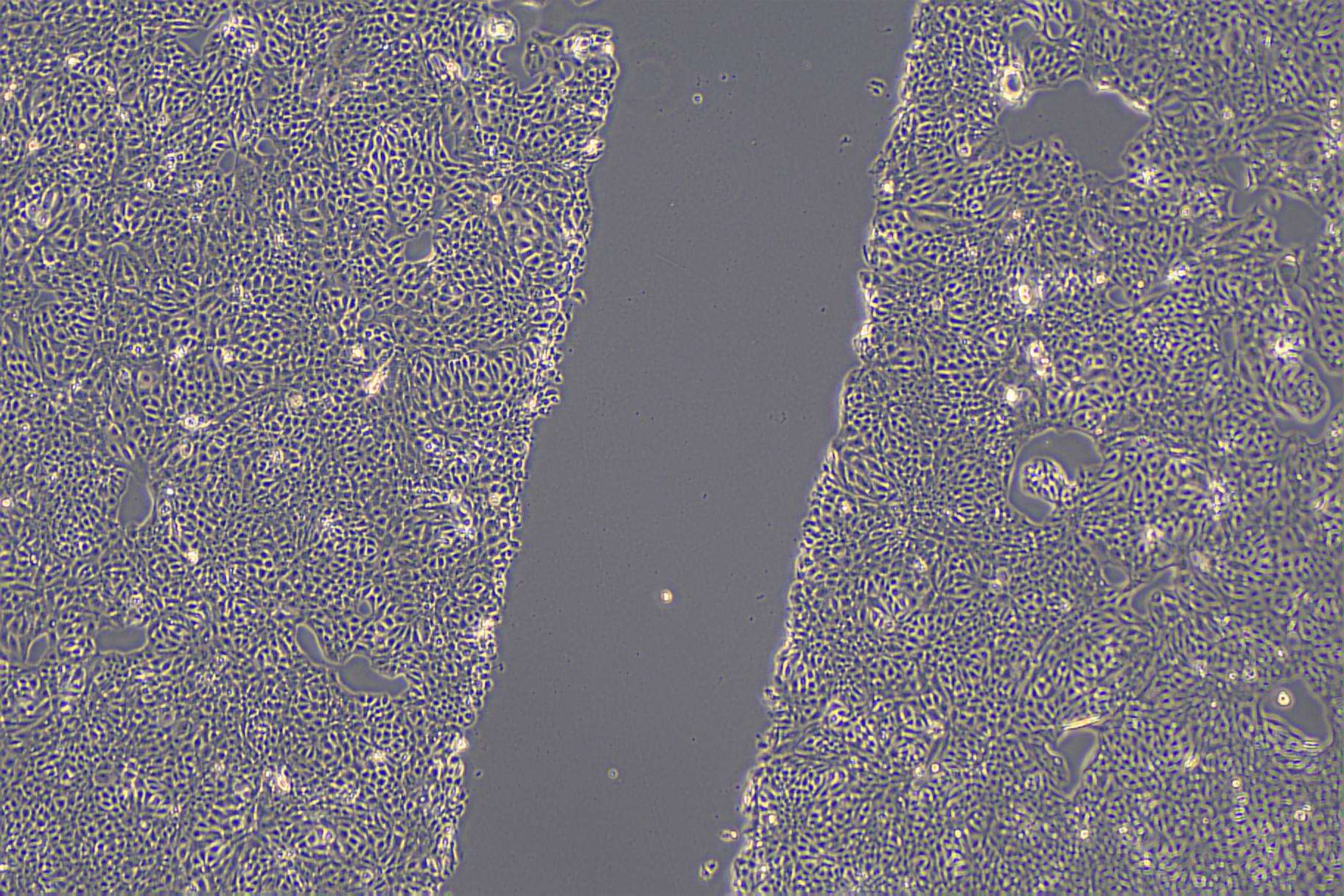

Supplement: Supplemental Information 1 — All the raw data, result images and running codes in this paper, including qRT-PCR data and cell behavior measurements. [file peerj-14-20538-s001.zip › Supplementary files 1/result 7/wound-healing/0h inhibitor+si.jpg]

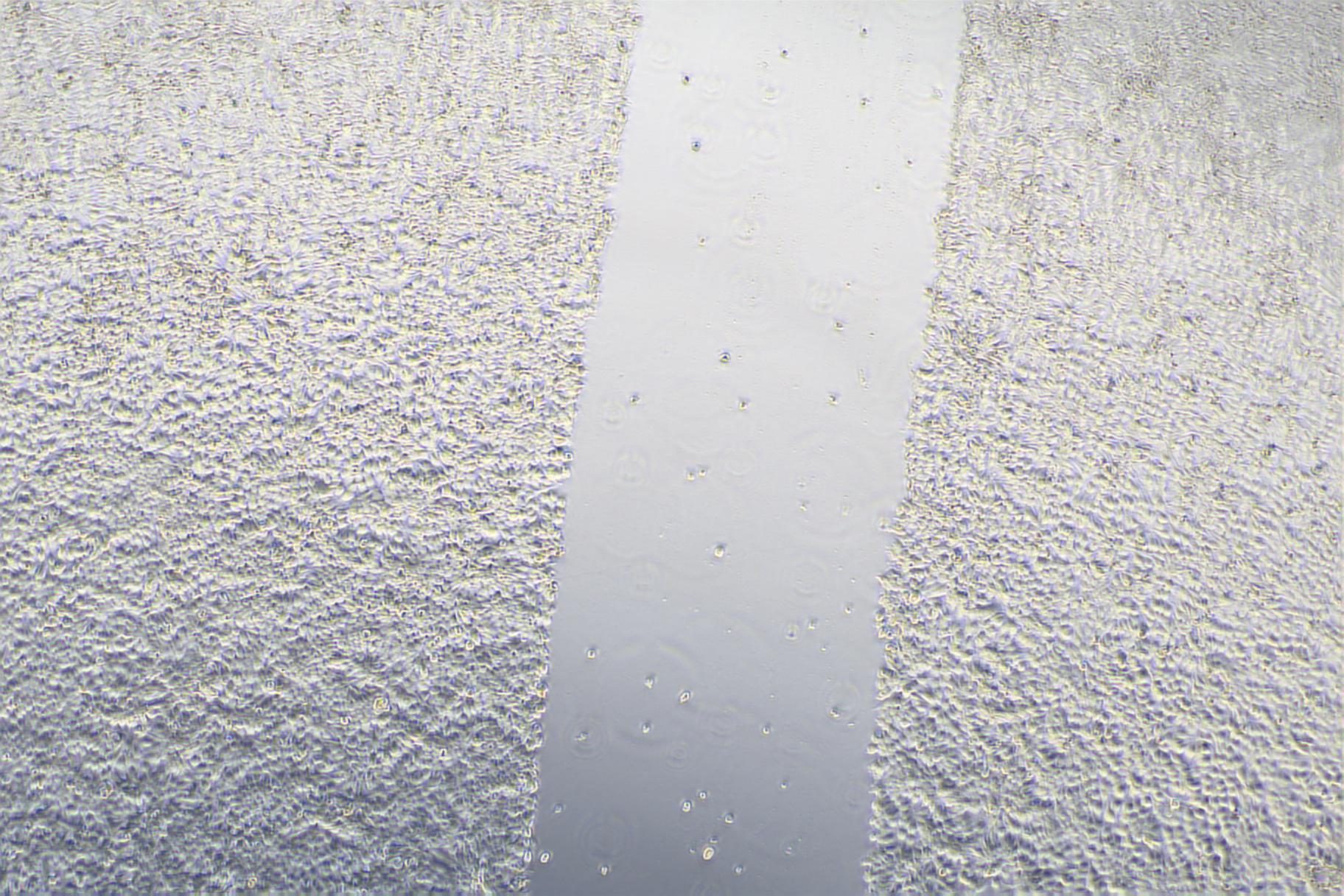

Supplement: Supplemental Information 1 — All the raw data, result images and running codes in this paper, including qRT-PCR data and cell behavior measurements. [file peerj-14-20538-s001.zip › Supplementary files 1/result 7/wound-healing/0h inhibitor.jpg]

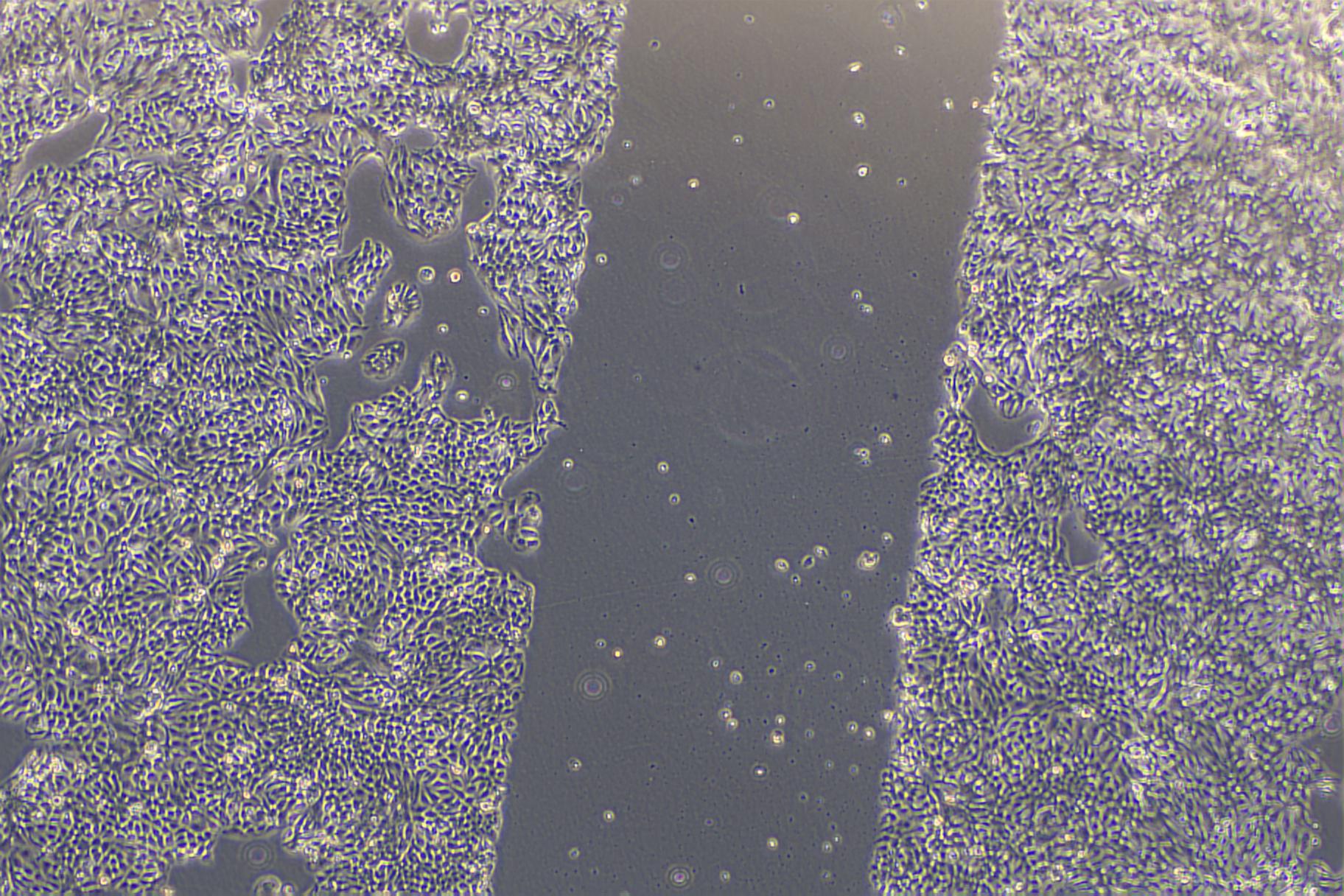

Supplement: Supplemental Information 1 — All the raw data, result images and running codes in this paper, including qRT-PCR data and cell behavior measurements. [file peerj-14-20538-s001.zip › Supplementary files 1/result 7/wound-healing/0h mimic NC.jpg]

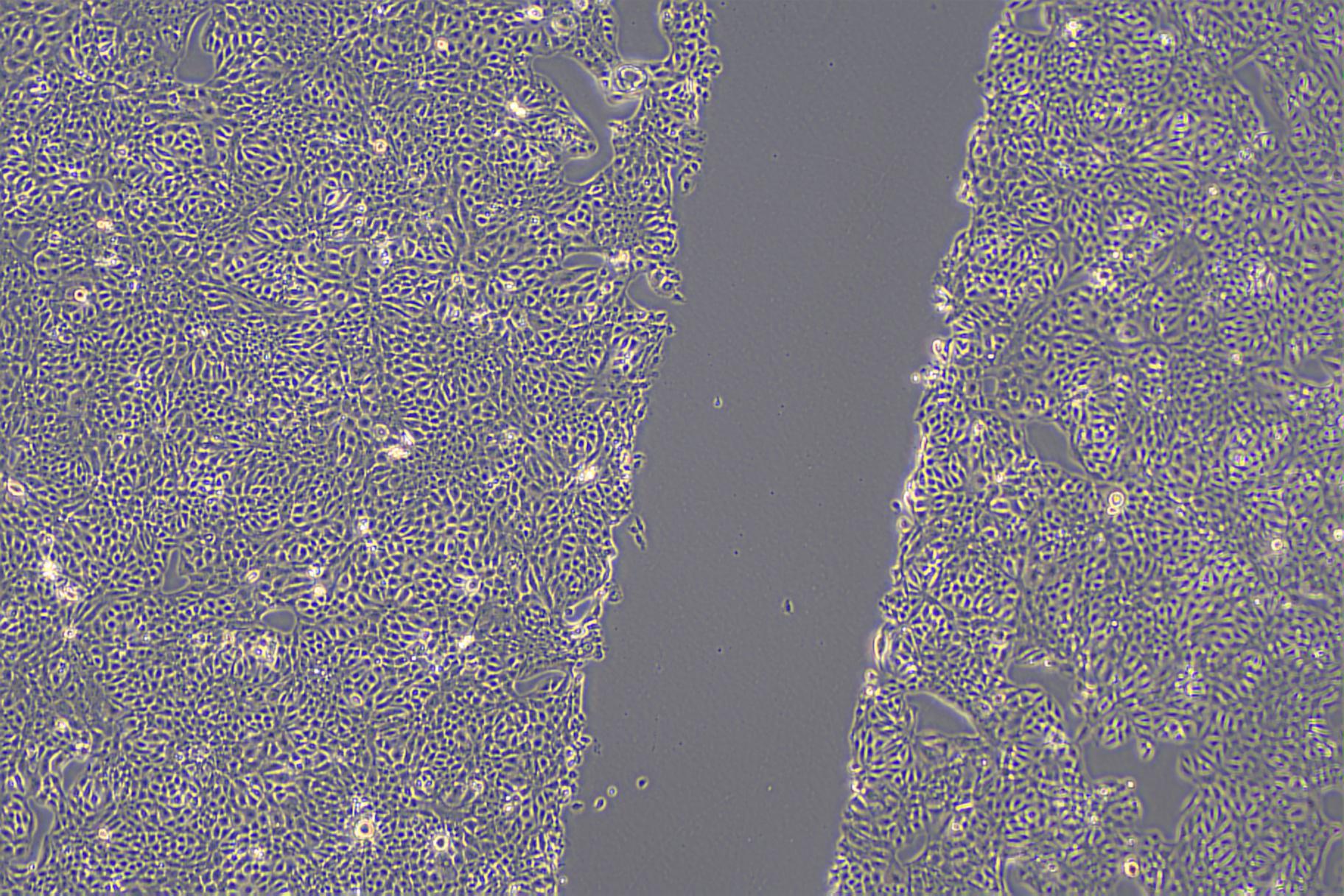

Supplement: Supplemental Information 1 — All the raw data, result images and running codes in this paper, including qRT-PCR data and cell behavior measurements. [file peerj-14-20538-s001.zip › Supplementary files 1/result 7/wound-healing/0h mimic+si.jpg]

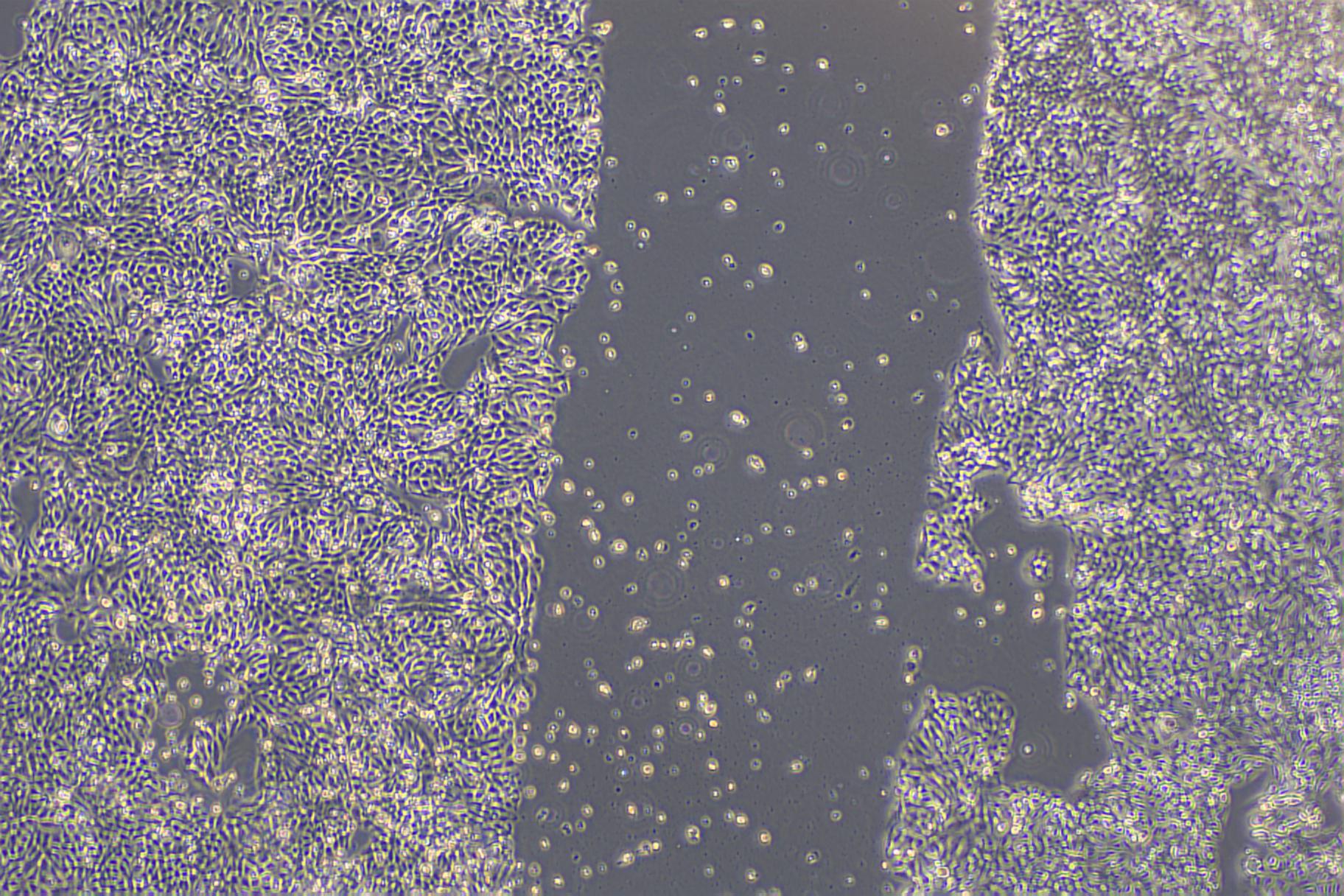

Supplement: Supplemental Information 1 — All the raw data, result images and running codes in this paper, including qRT-PCR data and cell behavior measurements. [file peerj-14-20538-s001.zip › Supplementary files 1/result 7/wound-healing/0h mimic.jpg]

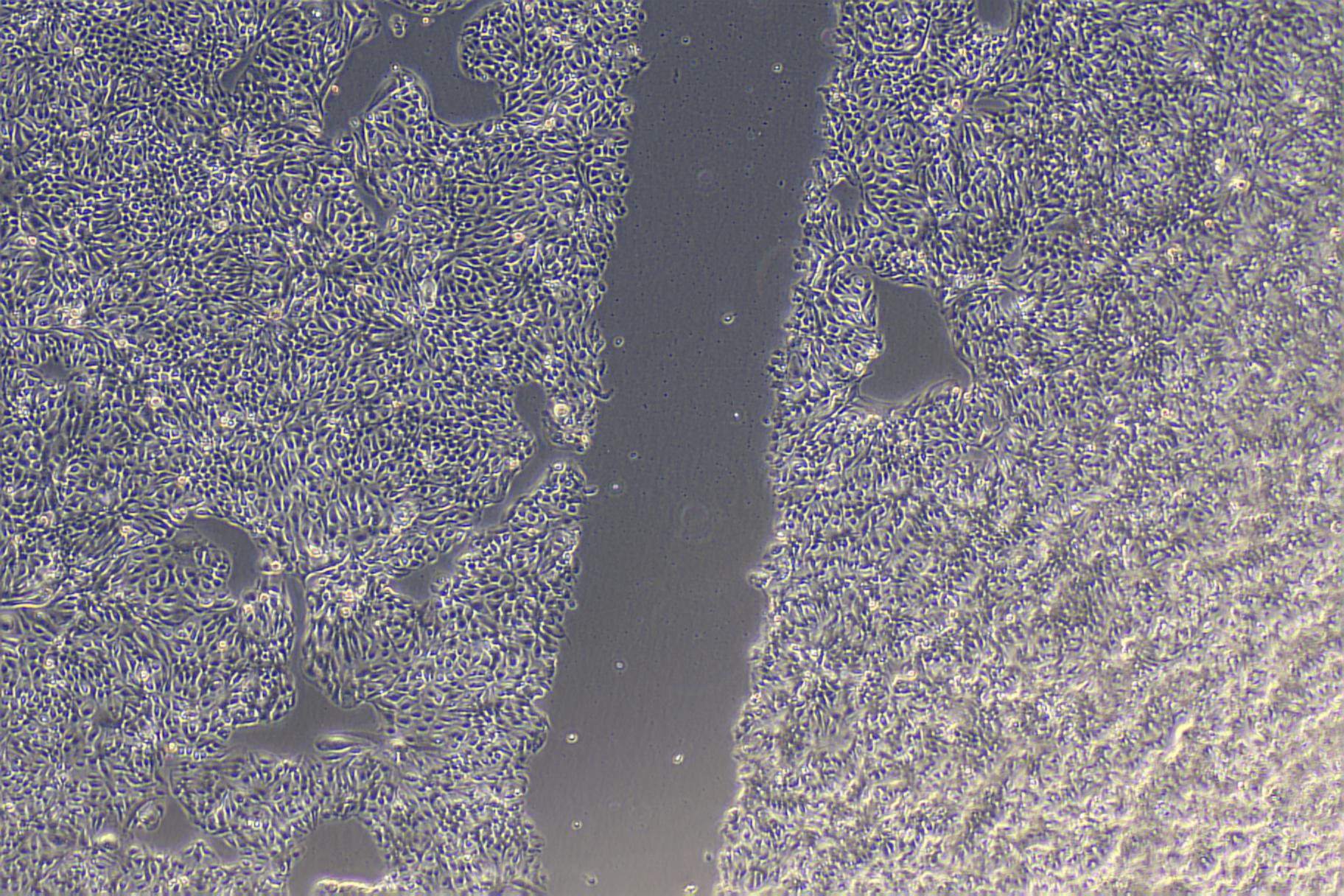

Supplement: Supplemental Information 1 — All the raw data, result images and running codes in this paper, including qRT-PCR data and cell behavior measurements. [file peerj-14-20538-s001.zip › Supplementary files 1/result 7/wound-healing/48h control.jpg]

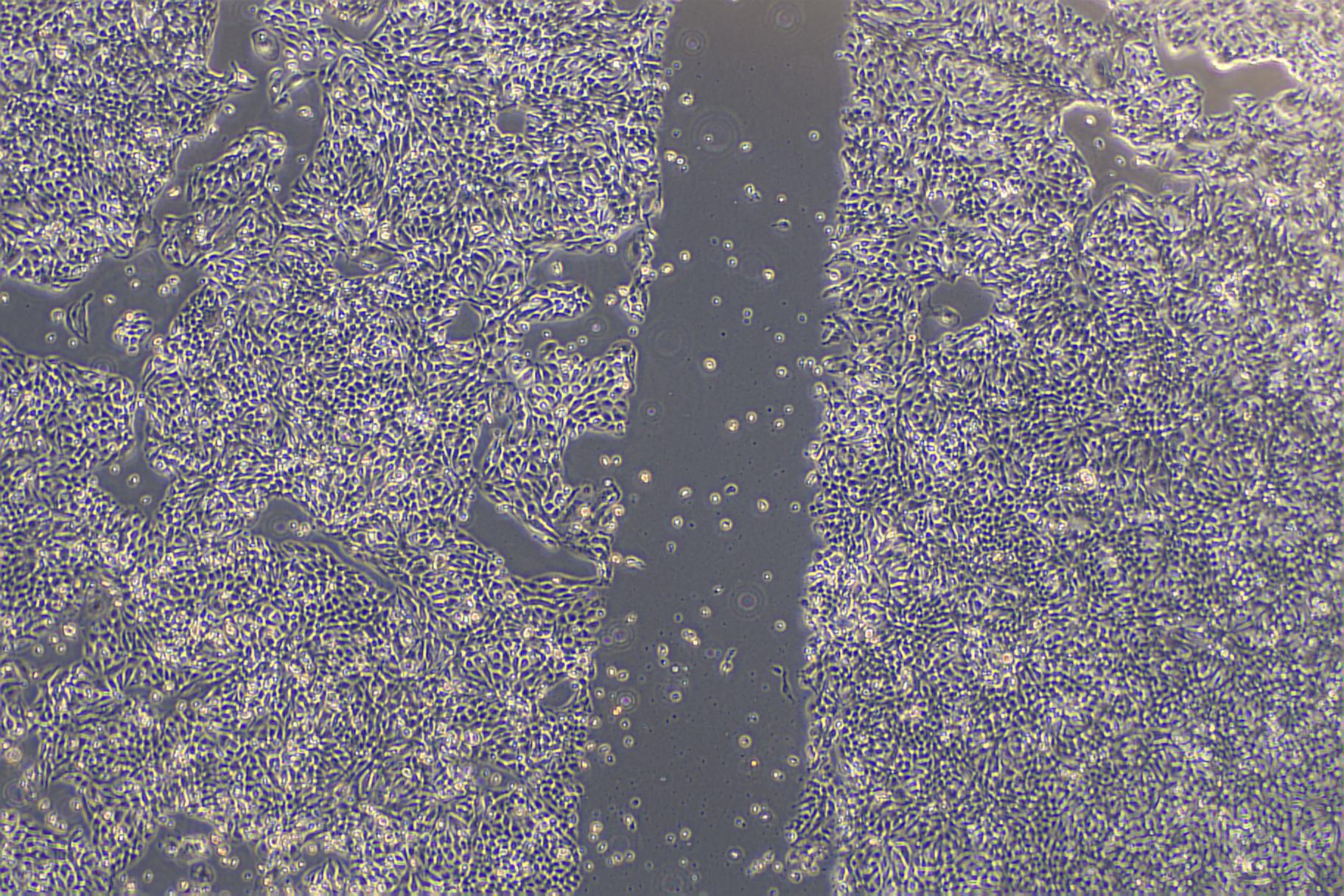

Supplement: Supplemental Information 1 — All the raw data, result images and running codes in this paper, including qRT-PCR data and cell behavior measurements. [file peerj-14-20538-s001.zip › Supplementary files 1/result 7/wound-healing/48h inhibitor NC.jpg]

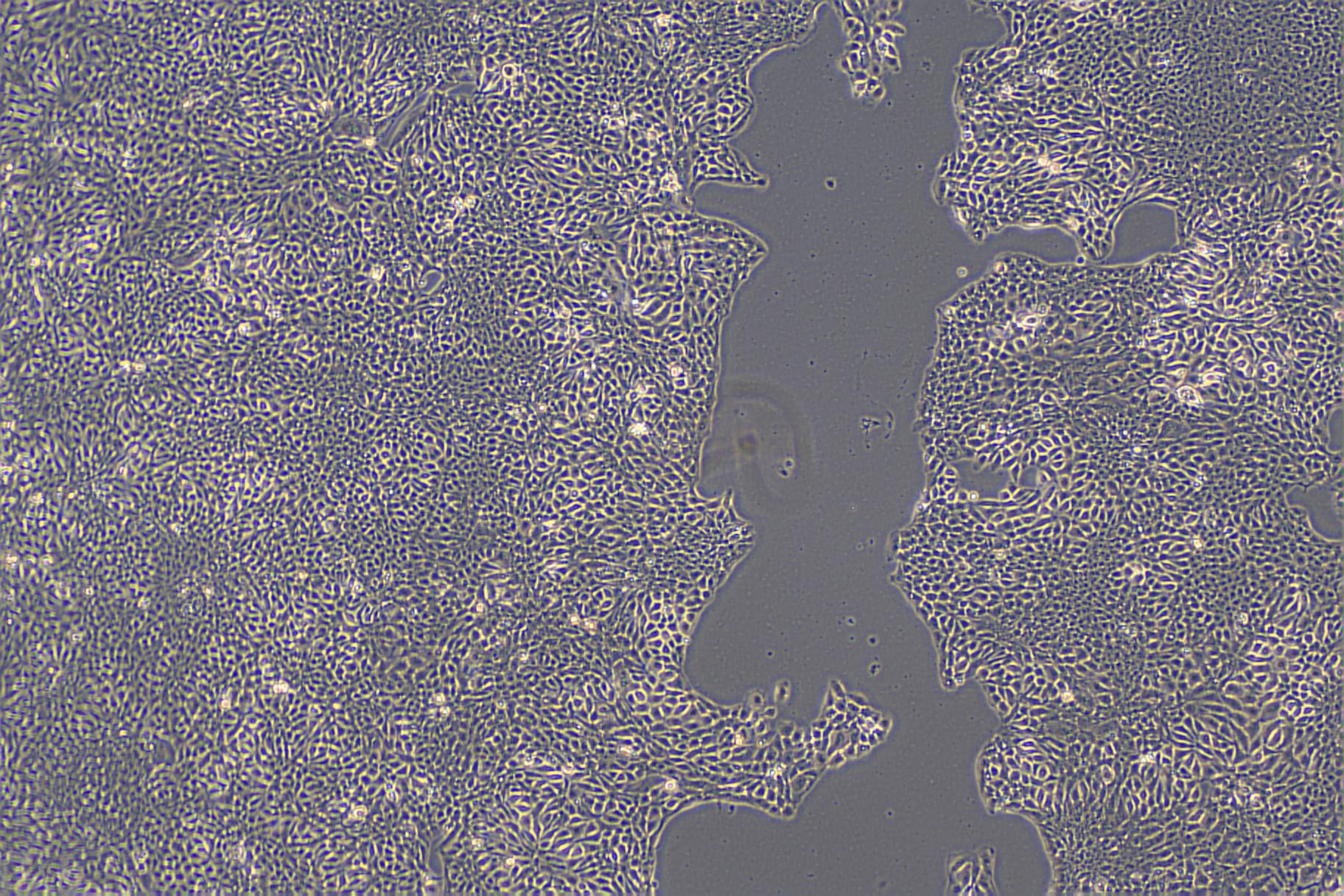

Supplement: Supplemental Information 1 — All the raw data, result images and running codes in this paper, including qRT-PCR data and cell behavior measurements. [file peerj-14-20538-s001.zip › Supplementary files 1/result 7/wound-healing/48h inhibitor+pCDH.jpg]

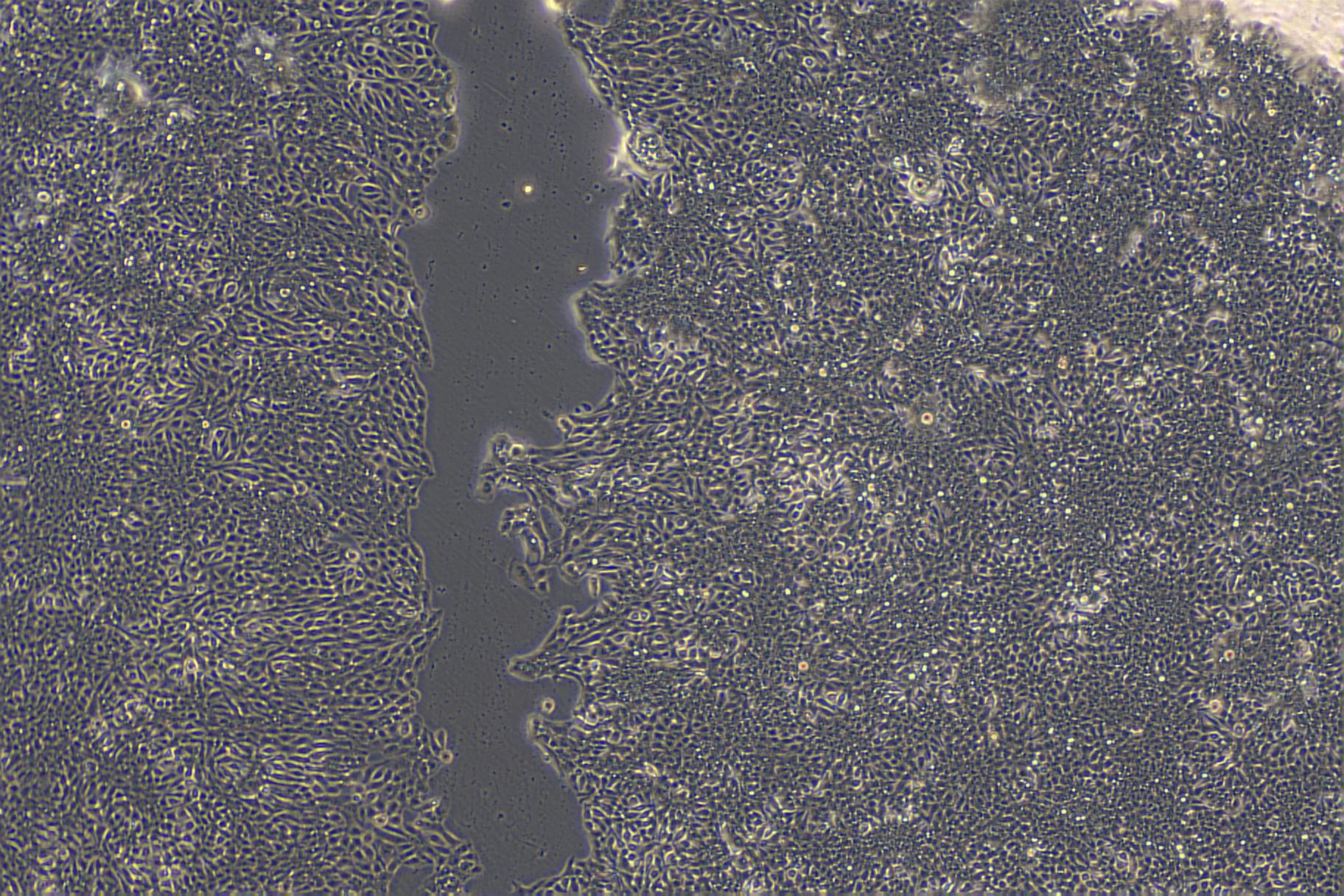

Supplement: Supplemental Information 1 — All the raw data, result images and running codes in this paper, including qRT-PCR data and cell behavior measurements. [file peerj-14-20538-s001.zip › Supplementary files 1/result 7/wound-healing/48h inhibitor+si.jpg]

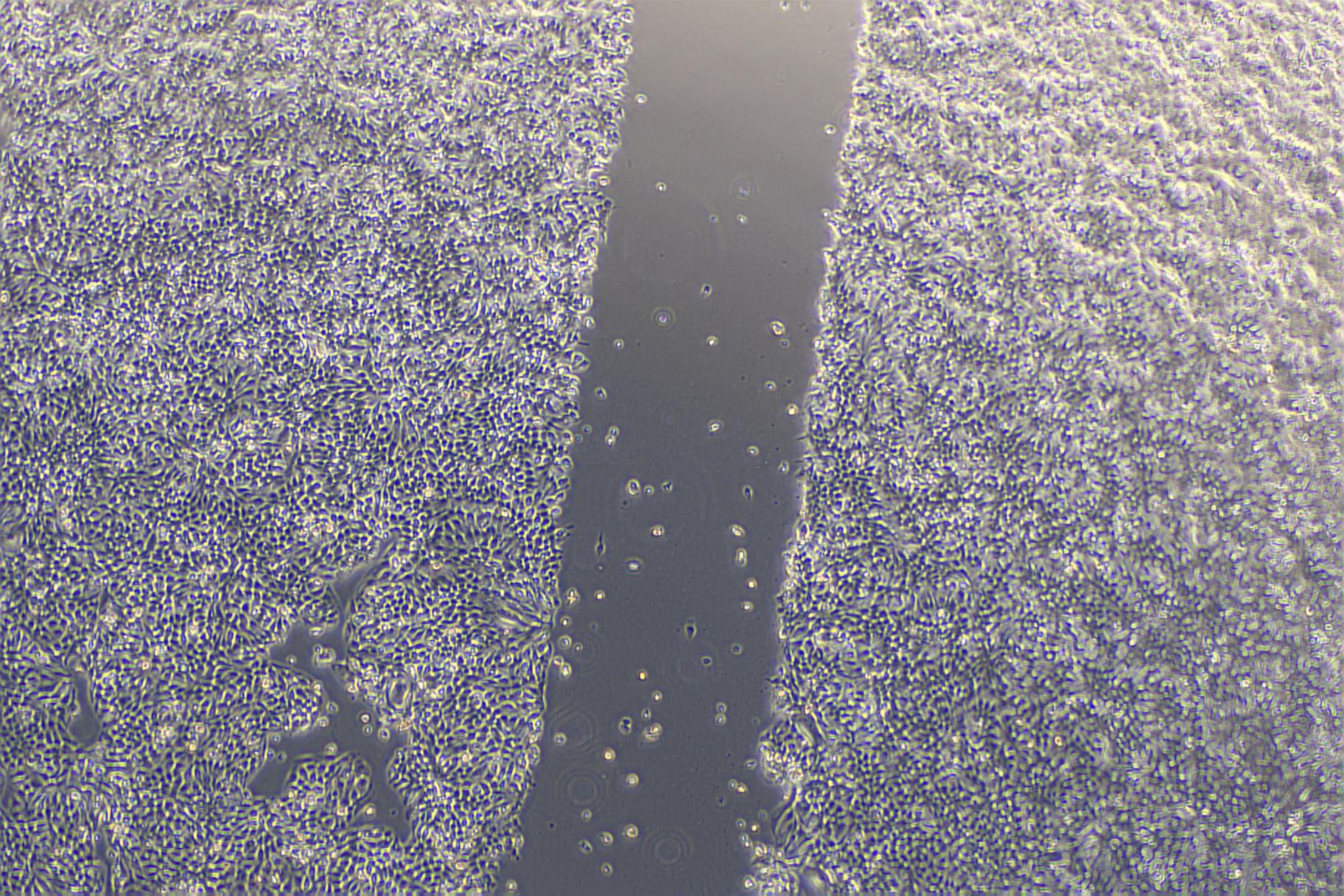

Supplement: Supplemental Information 1 — All the raw data, result images and running codes in this paper, including qRT-PCR data and cell behavior measurements. [file peerj-14-20538-s001.zip › Supplementary files 1/result 7/wound-healing/48h mimic NC.jpg]

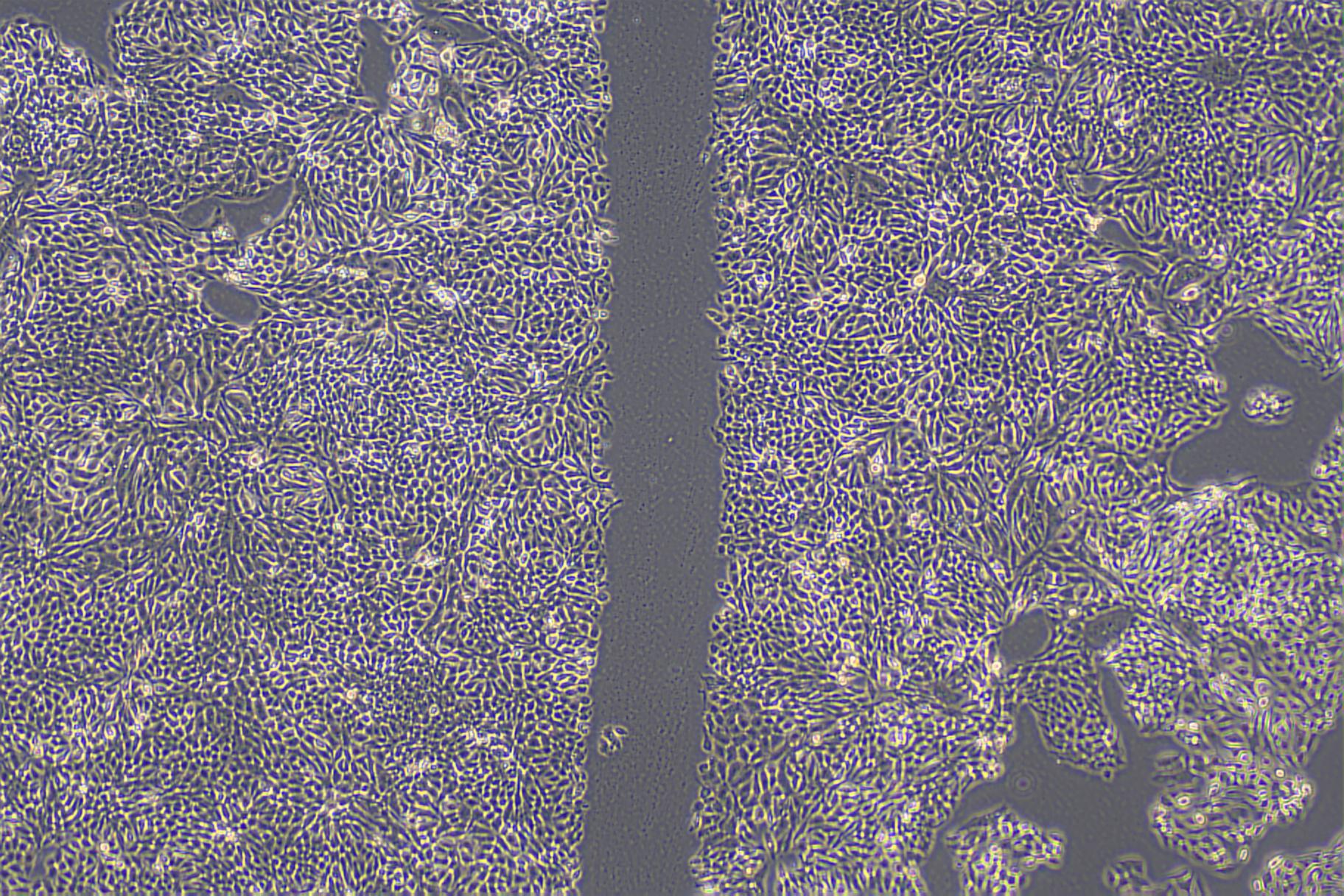

Supplement: Supplemental Information 1 — All the raw data, result images and running codes in this paper, including qRT-PCR data and cell behavior measurements. [file peerj-14-20538-s001.zip › Supplementary files 1/result 7/wound-healing/48h mimic+pCDH.jpg]

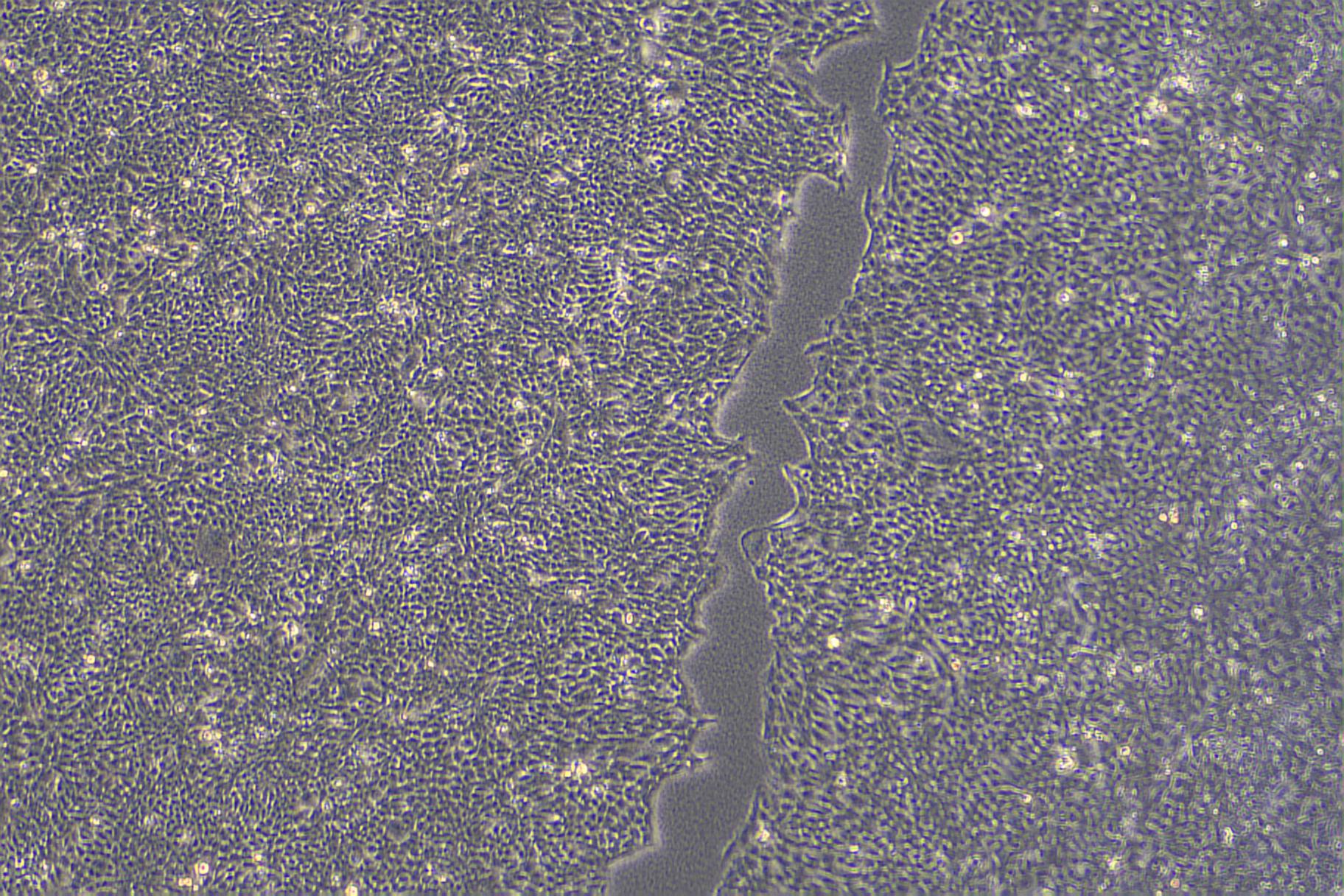

Supplement: Supplemental Information 1 — All the raw data, result images and running codes in this paper, including qRT-PCR data and cell behavior measurements. [file peerj-14-20538-s001.zip › Supplementary files 1/result 7/wound-healing/48h mimic+si.jpg]

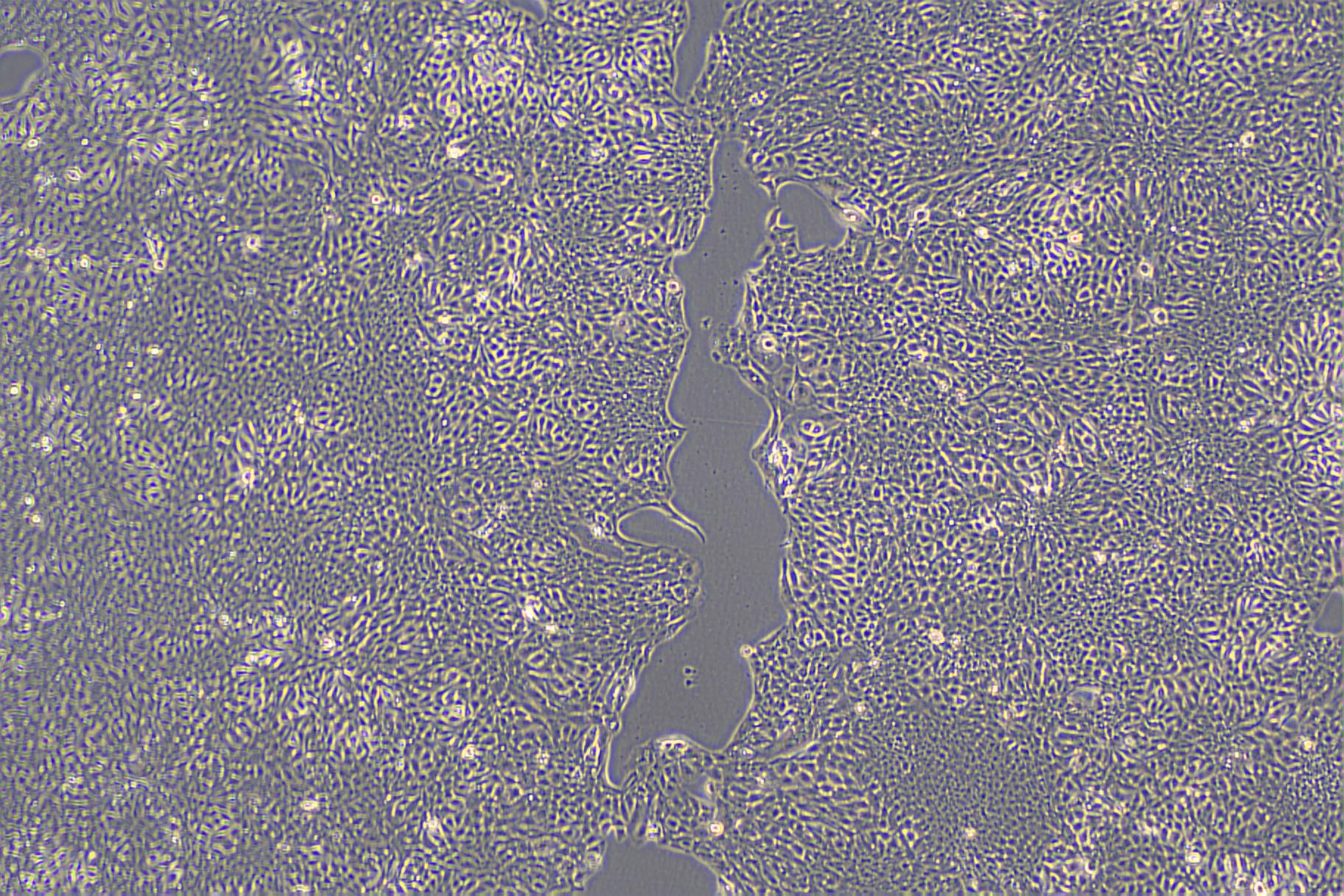

Supplement: Supplemental Information 1 — All the raw data, result images and running codes in this paper, including qRT-PCR data and cell behavior measurements. [file peerj-14-20538-s001.zip › Supplementary files 1/result 7/wound-healing/48h mimic.jpg]

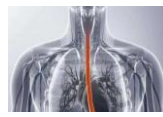

TCGA-ESCA

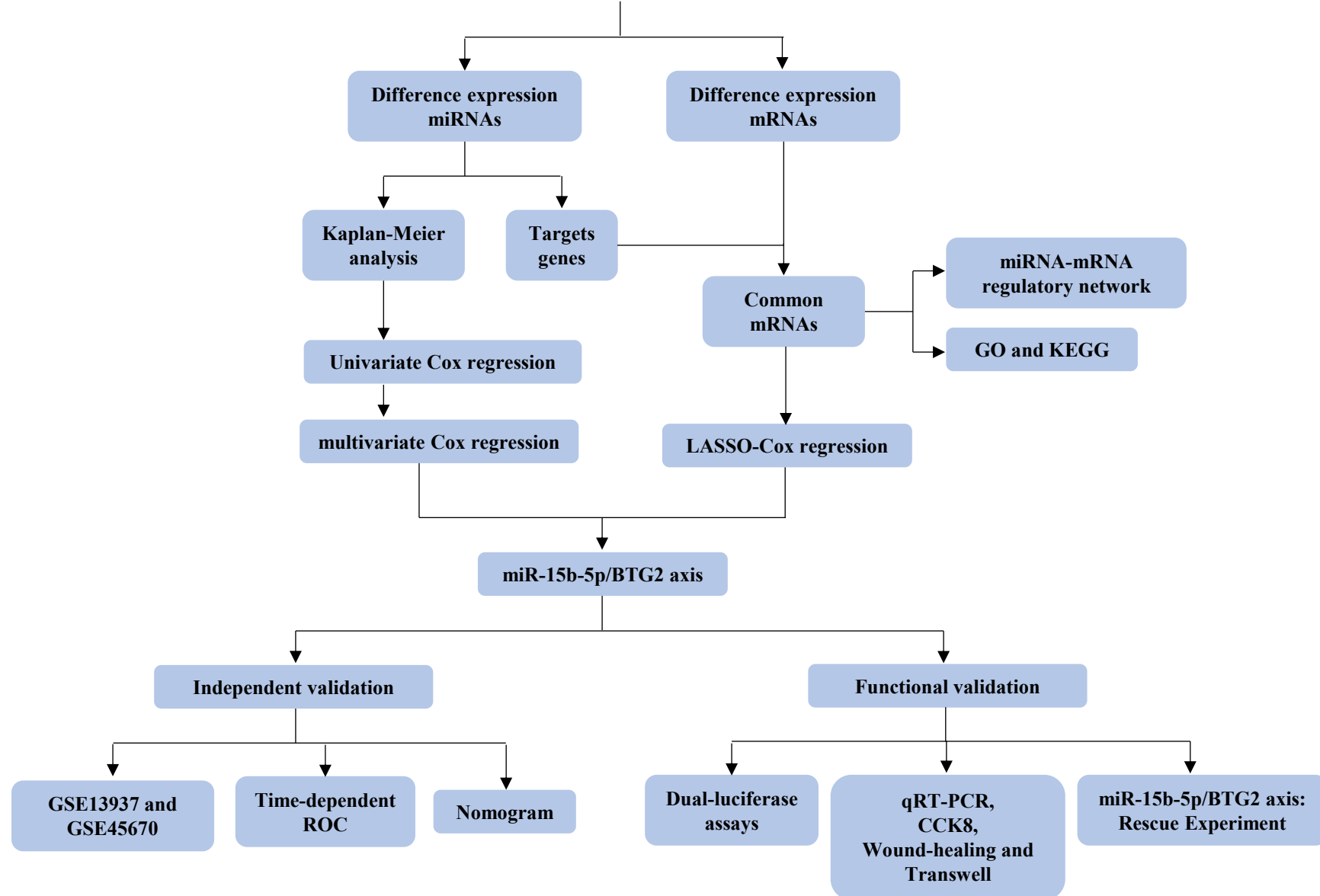

Supplement: Supplemental Information 3 [file peerj-14-20538-s003.pdf]

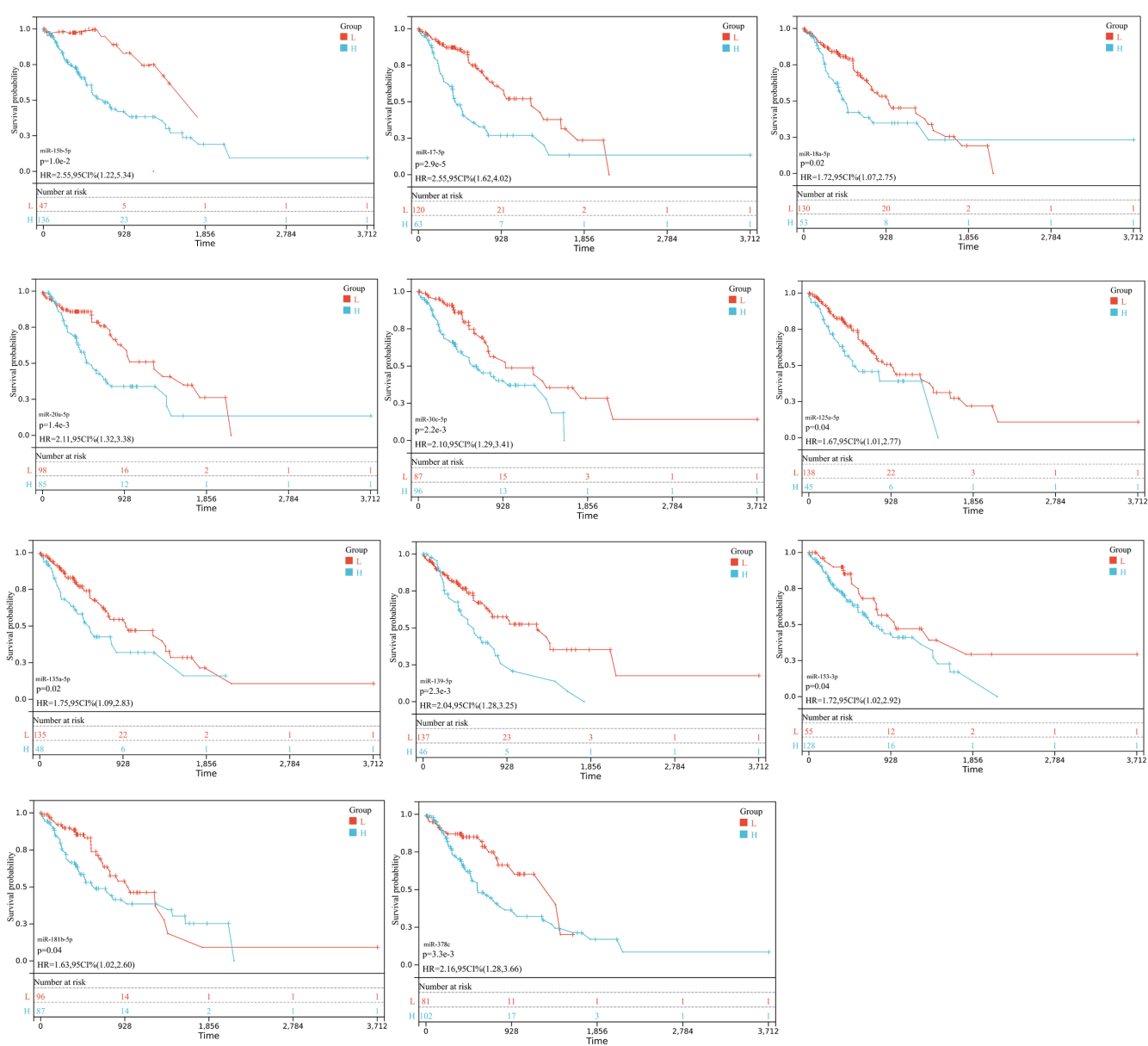

Supplement: Supplemental Information 4 [file peerj-14-20538-s004.pdf]

**A**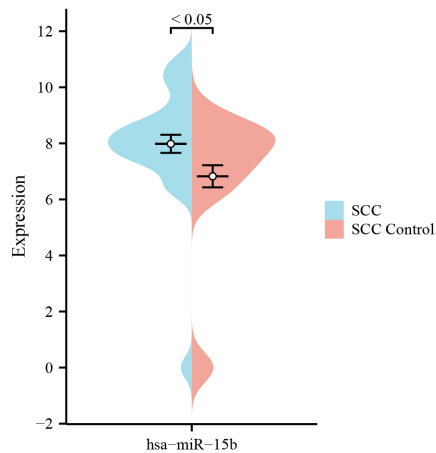**B**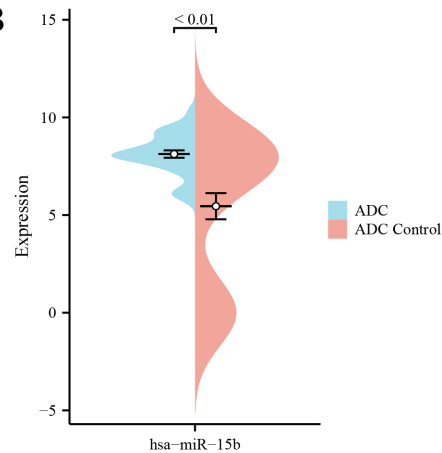**C**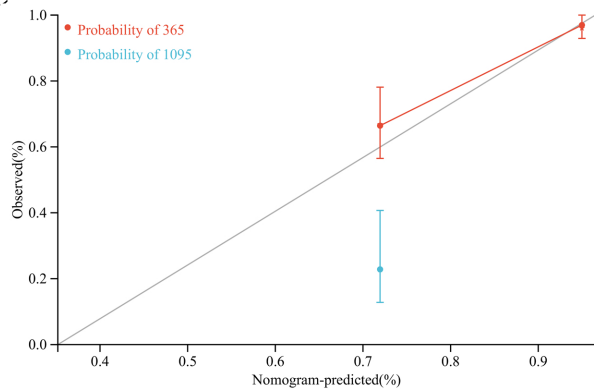**D**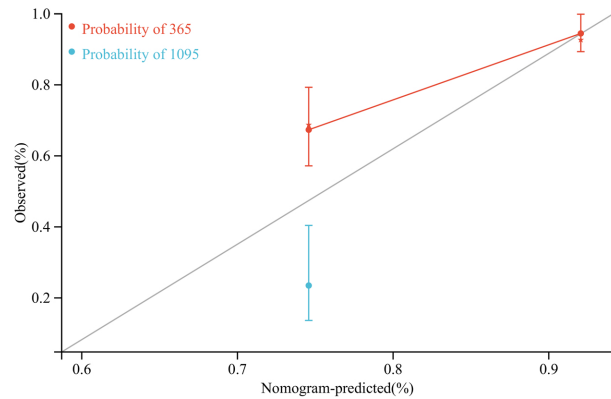

Supplement: Supplemental Information 6 — (B) miR-15b-5p is significantly upregulated in EAC samples from the GSE13937 dataset. (C) Calibration curve for the miR-15b-5p nomogram, illustrating the concordance between predicted and actual 1-, 3- and 5-year overall survival. (D) Calibration curve for the BTG2 nomogram, demonstrating the model’s predictive accuracy. [file peerj-14-20538-s006.pdf]
