# Supplementary material for: Integrative analysis for identification of key miRNA-mRNA regulatory axes in esophageal cancer and preliminary validation of the regulatory role of miR-15b-5p/BTG2 therein
Source: PeerJ. 2026 Jan 28;14:e20538. doi: 10.7717/peerj.20538 (PMC12860276; doi:10.7717/peerj.20538)
Supplement: Supplemental Information 2 [file peerj-14-20538-s002.doc]

**正在进行ID转换：**ID conversion in progress.

**共匹配到了276个基因ID,输出到文件：**A total of 276 gene IDs were matched and output to file.

**开始进行GO、KEGG富集分析：**Started GO and KEGG enrichment analysis.

**富集结果统计如下：**Enrichment results are summarized as follows.

**所有绘图完成：**All plots have been generated.

**获得矩阵：**Matrix obtained.

**行：**Rows

**列：**Columns

**开始进行多因素生存回归：**Started multivariate survival regression.

**多因素生存回归成功：**Multivariate survival regression successful.

**开始进行nomogram计算：**Started nomogram calculation.

**nomogram计算完成：**Nomogram calculation completed.

**获得nomogram数据：**Nomogram data obtained.

**保存nomogram数据：**Nomogram data saved.

**保存风险得分数据：**Risk score data saved.

**开始计算校准曲线：**Started calculating calibration curve.

**获得校准曲线数据：**Calibration curve data obtained.
